# A Multiplex RT-PCR Method for the Detection of Reptarenavirus Infection

**DOI:** 10.3390/v15122313

**Published:** 2023-11-25

**Authors:** Francesca Baggio, Udo Hetzel, Barbara Prähauser, Eva Dervas, Eleni Michalopoulou, Tanja Thiele, Anja Kipar, Jussi Hepojoki

**Affiliations:** 1 The BIBD Group and Institute of Veterinary Pathology, Vetsuisse Faculty, University of Zurich, 8057 Zurich, Switzerland; udo.hetzel@uzh.ch (U.H.); barbara.praehauser@uzh.ch (B.P.); eva.dervas@uzh.ch (E.D.); michalopoulou.e@unic.ac.cy (E.M.); tanja.thiele@uzh.ch (T.T.); anja.kipar@uzh.ch (A.K.); jussi.hepojoki@helsinki.fi (J.H.); 2 Center for Clinical Studies, Vetsuisse Faculty, University of Zurich, 8057 Zurich, Switzerland; 3 Department of Virology, Medicum, Faculty of Medicine, University of Helsinki, 00290 Helsinki, Finland

**Keywords:** reptarenaviruses, Boid Inclusion Body Disease (BIBD), multiplex RT-PCR, virus carriers, viral spread, snake collections, genetic diversity

## Abstract

Reptarenaviruses cause Boid Inclusion Body Disease (BIBD), a fatal disease of boid snakes with an economic and ecological impact, as it affects both captive and wild constrictor snakes. The clinical picture of BIBD is highly variable but often only limited. Intracytoplasmic inclusion bodies (IB), which develop in most cell types including blood cells, are the pathognomonic hallmark of BIBD; their detection represents the diagnostic gold standard of the disease. However, IBs are not consistently present in clinically healthy reptarenavirus carriers, which can, if undetected, lead to and maintain the spread of the disease within and between snake populations. Sensitive viral detection tools are required for screening and control purposes; however, the genetic diversity of reptarenaviruses hampers the reverse transcription (RT) PCR-based diagnostics. Here, we describe a multiplex RT-PCR approach for the molecular diagnosis of reptarenavirus infection in blood samples. The method allows the detection of a wide range of reptarenaviruses with the detection limit reaching 40 copies per microliter of blood. Using 245 blood samples with a reference RT-PCR result, we show that the technique performs as well as the segment-specific RT-PCRs in our earlier studies. It can identify virus carriers and serve to limit reptarenavirus spreading in captive snake collections.

## 1. Introduction

Boid Inclusion Body Disease (BIBD) is an untreatable and ultimately lethal disease known since the 1970s [1,2]. BIBD generally affects snakes of the families *Boidae* and *Pythonidae* in captivity, in both private and zoological collections [2,3,4,5,6,7,8,9]. However, we have recently confirmed that the disease also affects free-ranging indigenous boa constrictors, and using archival samples, we found strong evidence that the disease has been present in Costa Rica since at least the 1980s [10]. BIBD has a negative economic impact on snake collections, due to quarantine requirements and deaths, but also represents a potential ecological burden through compromising conservation efforts [3,10]. Pathomorphologically, BIBD is diagnosed based on the detection of pathognomonic electron-dense intracytoplasmic inclusion bodies (IBs) [1,11], which develop in most cell types of affected snakes, including red and white blood cells [3]. The latter allows for the ante mortem diagnosis of BIBD in routinely stained blood smears of individual snakes and for the BIBD screening of snake collections; it represents the current gold standard for BIBD diagnosis [2,3,7,9]. 

About a decade ago, three independent research groups, including ours, coincidentally identified novel arenaviruses as the potential etiological agent of BIBD [3,12,13]. The International Committee on Virus Taxonomy (ICTV) classified the novel viruses as the genus *Reptarenavirus* in the family *Arenaviridae* after it established the genus *Mammarenavirus* for the formerly known arenaviruses [14]. Currently, the family *Arenaviridae* comprises five recognized genera, *Mammarenavirus*, *Reptarenavirus*, *Hartmanivirus*, *Innmovirus* and *Antennavirus* (https://ictv.global/report/chapter/arenaviridae/arenaviridae, accessed on 1 May 2019, updated on September 2020 and on August 2023; [15]). Arenaviruses are enveloped viruses with a single-stranded bisegmented RNA genome of ambisense coding strategy, with the exception of antennaviruses and innmoviruses that have a trisegmented genome (https://ictv.global/report/chapter/arenaviridae/arenaviridae (accessed on 16 November 2023) and [15]). Large (L) and small (S) segments of the reptarenavirus genome encode the zinc finger matrix protein (ZP) and the RNA-dependent RNA polymerase (RdRp), and the nucleoprotein (NP) and the glycoprotein precursor (GPC), respectively (https://ictv.global/report/chapter/arenaviridae/arenaviridae (accessed on 16 November 2023) and [15]). The NP is the main component of the IBs in reptarenavirus-infected cells [3,12,16]. An immunohistological approach using specific antibodies against NP helps to visualize, in particular, small and/or few IBs in tissue specimens, assisting in the reliable diagnosis of BIBD when surgical biopsies have been taken, e.g., from the liver [2,17] or in post mortem examinations [3,5,18]. 

When first described, BIBD was reported in boa constrictors and pythons showing mostly fatal central nervous system (CNS) signs [1,2,8,10,19,20]. The disease is often associated with bacterial, fungal or protozoal infections [3,8,10,18], suggesting their secondary nature and underlying BIBD-associated immunosuppression [2,11]. Recent screening studies indicate that boa constrictors with a reptarenavirus infection can display only subtle clinical signs or remain clinically healthy for years, with or without overt IB formation [4,5,7,8,9,19,20]. Since reptarenavirus transmission can occur both horizontally, through common infection routes like saliva, skin or feces [9,19,21], or vertically [6,9], clinically healthy virus carriers represent an important uncontrolled risk factor for the spread of reptarenaviruses within and between collections. 

Reptarenavirus-infected snakes often carry a variety of genetically diverse S and L segments, with L segments often outnumbering the S segments [6,7,9,18,21,22]. Interestingly, an in silico study revealed that in individual infections, L segments are more genetically diverse than S segments [23]. The fact that a multitude of genetically variable reptarenavirus segments are found in snakes with BIBD [6,7,9,18,21,22] complicates the development of reverse transcription-PCR (RT-PCR)-based molecular diagnostic tools for the reliable detection of reptarenavirus infection. The use of multiple pairs of specifically designed primers for each S or L segment RT-PCR employed in our earlier studies [6,7] does not represent an economically viable diagnostic approach. We have failed to establish a “pan-reptarenavirus” RT-PCR approach using degenerate primers and were not successful in establishing an RT-PCR of sufficient sensitivity with the primers suggested by Stenglein and coauthors [21]. Instead, in a recent study, we used metatranscriptomics to identify the reptarenaviruses present in a large single-breeding collection of 183 boa constrictors through sequencing a subset of the animals. The obtained sequences then served to design tools for the RT-PCR-based screening of the entire collection [9]. Although we likely identified all infected animals, the approach failed in detecting all L segments in the collection. These earlier studies and the fact that variation in the S segment (both in terms of nucleotide sequence and abundance) appears lower than in the L segment [6,7,9,18,21] led us to attempt establishing a multiplex RT-PCR approach for the detection of S segment reptarenavirus genotypes. We designed and optimized the primer mix to achieve high sensitivity for the detection of those reptarenavirus species/segments that we have most commonly found in the European breeding collections and those identified by Stenglein and colleagues in the US [6,7,9,21]. We evaluated the performance of the multiplex RT-PCR by testing archival boa constrictor blood samples previously tested as positive or negative for a reptarenavirus infection by segment-specific RT-PCRs and qRT-PCRs, and compared the obtained results with those of our initial studies with specific primers. 

## 2. Materials and Methods

### 2.1. Animals and Samples

The study was conducted on 344 full blood samples from boa constrictors. The samples had been collected either ante or post mortem for diagnostic screening for BIBD and reptarenavirus infection upon the owners’ request. Blood samples had been taken from live animals by cardiocentesis/caudal tail vein venipuncture either by the veterinarian attending the colony [7,9] or as part of a project that offered snake owners the screening of their colony. The latter was performed under the approved animal experiments with the cantonal numbers ZH195/16 and ZH136/20 (national numbers 27229 and 32723) following the Swiss regulations regarding animal experimentation. Post mortem blood samples were collected from snakes that were euthanized upon the owners’ request after being diagnosed with BIBD through cytological examination of blood smears [3,9,10] or with the aim to exclude any disease, in particular BIBD, and to obtain information on the general health status in their facility. These animals had been submitted by the owners for euthanasia and a full diagnostic post mortem examination; here, the blood was collected from the animal after deep anesthesia and decapitation. Blood samples were stored in EDTA tubes at −80 °C until analyzed.

Most blood samples used in this study were from animals described in our former work, including BIBD-positive snakes where next-generation sequencing (NGS) and de novo assembly or reverse transcription (RT)-PCR-based approaches had served to identify the reptarenavirus L and S segments carried [7,9,10]. For one animal (W4, [10]), for which a blood sample was not available, pancreas and brain biopsies were used instead.

### 2.2. Ethics Statement

Several specimens of this work originate from snakes of private collections that were submitted by their owners to the Institute of Veterinary Pathology, Vetsuisse Faculty, University of Zurich, Switzerland, for a diagnostic post mortem examination. These snakes were euthanized according to the ASPA, Animals (Scientific Procedures) Act 1986, schedule 1 (appropriate methods of humane killing, http://www.legislation.gov.uk/ukpga/1986/14/schedule/1 (accessed on 16 November 2023), latest revised version on 5 November 2015) procedure. The owners gave full informed consent for the diagnostic post mortem analyses and the use of material from the necropsied animals for research purposes. Both euthanasia and post mortem examinations did not require ethical permission because they are routine veterinary procedures and the animals were suspected to carry BIBD. 

### 2.3. Cell Culture and Viruses

I/1Ki cells derived from *Boa constrictor* kidney [3] were maintained at 30 °C and 5% CO_2_ in Minimum Essential media (MEM, Gibco, Thermo Fisher Scientific, Waltham, MA, USA) supplemented with 10% fetal bovine serum (FBS, Biochrom, Cambridge, UK), 10% tryptose phosphate broth (TPB, BD Difco, Franklin Lakes, NJ, USA), 6 mM Hepes (Biochrom, Cambridge, UK), 2 mM L-alanyl-L-glutamine (Biochrom, Cambridge, UK) and 50 µg/mL Gentamicin (Gibco, Thermo Fisher Scientific, Waltham, MA, USA), as described [24]. 

For viral stock preparation, a I/1Ki cell monolayer at 80% approximate confluency in 75 cm^2^ flasks was inoculated with each one of the following reptarenavirus single isolates at a multiplicity of infection (MOI) of approximately 0.1 to 10: Aurora borealis virus 1 (ABV-1, GenBank accession no. S segment KR870010 and L segment KR870021) [22,25], University of Helsinki virus 1 (UHV-1, S segment KR870011 and L segment KR870020) [22,25], University of Helsinki virus 2 (UHV-2, S segment KR870016 and L segment KR870030) [22,25,26], University of Giessen virus 1 (UGV-1, S segment KR870012 and L segment KR870022) [22,24,25,26] and University of Giessen virus 2 (UGV-2, S segment KR870015 and L segment KR870029) [22]. For each virus isolate, viral supernatant was collected at three-day intervals between 3 and 15 days post inoculation (dpi), and the collections were pooled together, aliquoted and stored at −80 °C.

In addition, a subset of blood samples of the Thiele et al. study [9], stored at −80 °C and previously frozen and thawed at least three times, was individually mixed (using approximately 200 µL blood per sample) with cell culture media of I/1Ki cells that were seeded on a single well of a 6-well plate at approximately 70–80% confluency. The following blood samples were used for inoculation: two samples from BIBD-positive animals with high reptarenavirus RNA level (for nos. 21 and 56, blood containing approximately 3.8 × 10^8^ and 1.7 × 10^10^ UGV/S6 (University of Giessen virus or S6, according to the nomenclature suggested by Stenglein et al. [21]) S segment RNA copies, respectively, based on the previous study [9], were used for inoculation), one sample from a BIBD-negative reptarenavirus carrier with high reptarenavirus RNA level (no. 109, used at approximately 7.4 × 10^9^ UGV/S6 S segment RNA copies/well), five samples from BIBD-negative reptarenavirus carriers with low reptarenavirus RNA level (nos. 22, 36, 143, 175 and 183, with approximately 2.3 × 10^4^, 2.2 × 10^3^, 2.1 × 10^4^, 1.1 × 10^4^ and 1.8 × 10^5^ UGV/S6 S segment RNA copies/well, respectively) and two BIBD- and reptarenavirus-negative samples (nos. 52 and 155). Cell culture supernatants and cells were collected at 6 and 18 dpi for viral RNA isolation and the preparation and processing of cell pellets for immunocytochemistry. 

### 2.4. Synthesis of Control RNAs

The following synthetic gene sequences were ordered in a pUC57 vector (Gene Universal, Newark, DE, USA) under the T7 promoter (TAATACGACTCACTATAG) and with a PmeI (GTTTAAAC) restriction site at the 3′ end: tavallinen suomalainen mies virus 2 (TSMV-2) S segment (472 nucleotides (nt), 770 to 1241 of GenBank accession no. NC_039007), S5-like (472 nt, 836 to 1307 of GenBank accession no. MH483080, 840 to 1311 of MH483063 and MH483067, 793 to 1264 of KX527581 and 811 to 1282 of KX527579), S2-like (472 nt, 862 to 1333 of GenBank accession no. MH483056), S7- and S10-like (472 nt, 781 to 1252 of GenBank accession nos. MH483088 and MH503957), UGV/S6 from Costa Rica (UGV/S6-CR) S segment (472 nt, 890 to 1361 of GenBank accession nos. MW09147 and OM456554, 874 to 1345 of OM456548, 886 to 1357 of OM456549, 873 to 1344 of OM456552 and OM456561 and 889 to 1360 of OM456556).

Plasmid linearization and purification steps, in vitro transcription of the control RNAs from the T7 promoter and the subsequent RNA purification were performed as described [25] and the RNAs were stored at −80 °C. The RNA concentrations were measured with the Invitrogen Qubit^TM^ 4 Fluorometer using the Qubit^TM^ RNA Broad Range (BR) Assay kit (20–1000 ng range, Invitrogen, Thermo Fisher Scientific, Waltham, MA, USA). An online calculation tool (http://endmemo.com/bio/dnacopynum.php, accessed on 16 December 2021) served to convert the values for the RNA concentrations to the number of copies of the control S segments.

### 2.5. RNA Extraction 

RNA was extracted from full blood samples as well as the brain and pancreas tissue samples of animal W4 [10], as previously described [9,10], with an initial TRIzol reagent-based extraction (Thermo Fisher Scientific, Waltham, MA, USA) followed by column-based RNA purification with the RNeasy Mini kit (Qiagen, Venlo, The Netherlands). During the RNA extraction, a subset of blood samples was subjected to an additional step of on-column DNase I (Qiagen, Venlo, The Netherlands) digestion, following the manufacturer’s instructions for the RNeasy Mini kit (Qiagen, Venlo, The Netherlands). RNA sample concentrations were determined as described in Section 2.4. For samples where the instrument indicated an RNA concentration too low for proper measurement, the RNA quantification was repeated using the Qubit^TM^ RNA High Sensitivity (HS) Assay kit (5–100 ng range, Invitrogen, Thermo Fisher Scientific, Waltham, MA, USA). The RNA concentrations of the samples varied between 2.3 and 600 ng/µL.

Cell culture supernatants for viral RNA extraction were collected from I/1Ki cells inoculated either with each one of the single isolates, ABV-1, UGV-1, UGV-2, UHV-1 and UHV-2, or incubated with snake blood samples from a previous study (see Section 2.3) [9]. RNA was isolated with the QIAamp Viral RNA Mini Kit (Qiagen, Venlo, The Netherlands), following the manufacturer’s instructions and inclusion of carrier RNA. The final elution volume (80 µL) was obtained by incubating and spinning the columns twice with 40 µL nuclease-free water. 

### 2.6. Quantitative Reverse Transcription PCR (qRT-PCR)

For each of the purified RNAs from ABV-1, UGV-1, UGV-2, UHV-1 and UHV-2 single isolates, the number of S segments per ml of cell culture supernatant was determined by qRT-PCR analyses that employed the primers (Microsynth AG, Balgach, Switzerland) and probes (Metabion International AG, Planegg, Germany) recently described [25]. The probes were labelled with 6-FAM [carboxyfluorescein] at the 5′ and TAMRA [6-carboxytetramethylrhodamine] at the 3′ end. The in vitro transcribed RNAs [25] served for generating standard curves (range 10^9^–10^2^ copies/reaction, 10-fold dilution series) to allow conversion of cycle threshold values to reptarenavirus S segment numbers per ml of cell culture supernatant. The 10 µL reaction mixes contained: 2.5 μL TaqMan Fast Virus 1-step master mix (Applied Biosystems, Thermo Fisher Scientific, Waltham, MA, USA), 2.5 μL RNA template, either 0.9 μM (for UGV-1, UGV-2, UHV-1 and UHV-2 S segments) or 0.5 μM (for ABV-1 S segment) final concentrations of forward and reverse primer and 0.25 μM final concentration of probe. The qRT-PCR reactions were run in duplicates in MicroAmp™ Fast Optical 96-well reaction plates (Applied Biosystems, Thermo Fisher Scientific, Waltham, MA, USA) on a 7500 Fast real-time PCR system and software v2.0 (Applied Biosystems, Thermo Fisher Scientific, Waltham, MA, USA) with the following cycling conditions: (1) 50 °C for 5 min; (2) 95 °C for 20 s; (3) 95 °C for 3 s; and (4) 60 °C for 30 s (steps 3 and 4 were repeated 42 times).

### 2.7. cDNA Synthesis and Multiplex RT-PCR

Snake tissue-derived RNA, single-reptarenavirus RNA genomes from cell culture supernatants (ABV-1, UGV-1, UGV-2, UHV-1 and UHV-2) and control RNAs (TSMV-2, S5-like, S2-like, S7- and S10-like, UGV/S6-CR) were subjected to cDNA synthesis with random hexamer primers using the RevertAid RT kit (Thermo Fisher Scientific, Waltham, MA, USA), following the manufacturer’s instructions. Briefly, the RNA samples were initially mixed with random hexamer primers and nuclease-free water, incubated at 65 °C for 5 min, and immediately placed on ice. Afterwards, the other components of the kit were added and the final reactions incubated at 25 °C for 5 min, followed by incubation at 42 °C for 60 min and heating at 70 °C for 5 min. For RNA concentrations >10 ng/µL, 5 µL and for RNA concentrations ≤10 ng/µL, 11 µL of each RNA sample was used. Reverse transcription no template control (RT NTC), containing every reagent for the reverse transcription reaction except for the RNA template, and reverse transcriptase minus (RT−) negative controls, containing every reagent for the reverse transcription reaction except the RT enzyme, were included. 

The primers for the “multiplex RT-PCR primer mix” given in Table 1 were designed based on the reptarenavirus S segment sequences available in GenBank (https://www.ncbi.nlm.nih.gov/genbank/ (accessed on 16 November 2023), National Center for Biotechnology Information (NCBI), Bethesda, MD, USA) using the Unipro UGENE 36 software (http://ugene.net/ (accessed on 16 November 2023)) and MUSCLE alignment tool. The forward and reverse primers were designed with the aim to obtain amplicon lengths of approximately 140 base pairs (bp) and a melting temperature (T_m_) of approximately 60 °C when using the Phusion DNA polymerase enzyme (Table 1). The calculation of the primer T_m_ relative to the Phusion DNA polymerase and primer concentration was provided by the T_m_ Calculator (https://www.thermofisher.com/ch/en/home/brands/thermo-scientific/molecular-biology/molecular-biology-learning-center/molecular-biology-resource-library/thermo-scientific-web-tools/tm-calculator.html (accessed on 16 November 2023), Thermo Fisher Scientific, Waltham, MA, USA). Multiple primer Analyzer (https://www.thermofisher.com/ch/en/home/brands/thermo-scientific/molecular-biology/molecular-biology-learning-center/molecular-biology-resource-library/thermo-scientific-web-tools/multiple-primer-analyzer.html (accessed on 16 November 2023), Thermo Fisher Scientific, Waltham, MA, USA) served for checking self- and cross-dimer formation of the primers. The primer sequences and further details on their relative position with respect to the reptarenavirus S segments that were used as a reference for primer design and to the reptarenavirus sequences with one, two or three mismatches, determined using BLAST analyses (https://blast.ncbi.nlm.nih.gov/Blast.cgi (accessed on 16 November 2023), National Library of Medicine, NCBI, Bethesda, MD, USA), are provided in Table 1 and Table S1, respectively.

The multiplex RT-PCR reactions were prepared in a 20 µL total volume with the following components: 10 µL 2× Phusion Flash High-Fidelity PCR Master Mix (Thermo Fisher Scientific, Waltham, MA, USA), 1 µL cDNA samples from snake blood or tissue samples, and 1.6 µL multiplex RT-PCR primer mix with a final concentration of 0.4 µM for forward (F) primers 1–5 and reverse (R) primers 1–6 and of 0.2 µM for F6 and R7. For each one of the viral stocks (ABV-1, UGV-1, UGV-2, UHV-1 and UHV-2) or in vitro-synthesized RNAs (TSMV-2, S5-like, S2-like, S7- and S10-like and UGV/S6-CR), both used to establish the multiplex RT-PCR protocol detection limit, 2 µL cDNAs of each dilution of a 10-fold series (ranging between 2 × 10^5^ to 2 S segment copies per reaction) were used for each 20 µL total volume multiplex RT-PCR reaction. 

Reactions were run on a Biometra trio thermocycler (Analytik Jena, Jena, Germany) with a three-step protocol with the following cycling conditions: (1) initial denaturation at 98 °C for 10 s; (2) denaturation at 98 °C for 1 s; (3) annealing at 58 °C for 2 s; (4) extension at 72 °C for 3 s; (5) final extension at 72 °C for 1 min. Steps 2–4 were repeated 38 times. The reptarenavirus S segment amplicons were separated by standard agarose gel electrophoresis (Tris-acetate-EDTA [TAE] buffer, 2.3% agarose, 1× GelRed nucleic acid gel stain (Biotium, Fremont, CA, USA)), using 0.25 µg/lane GeneRuler 100 bp plus DNA ladder (Thermo Fisher Scientific, Waltham, MA, USA); 10 µL of each amplification product were loaded per lane. Agarose gel analyses and image acquisition were performed using the UVP BioDoc-It Imaging System UV Transilluminator (Analytik Jena, Jena, Germany). To further confirm the presence of reptarenavirus sequences, Sanger sequencing (Microsynth AG, Balgach, Switzerland) was occasionally performed, using single primers of the multiplex RT-PCR primer mix.

### 2.8. Immunocytochemistry

For the preparation of cell pellets from the I/1 Ki cells incubated with snake blood samples from our previous study ([9], see Section 2.3), cells at 6 and 18 dpi were trypsinized (0.25% Trypsin-EDTA 1×, Gibco, Thermo Fisher Scientific, Waltham, MA, USA) for 5 min at room temperature, collected and spun at 1000× *g* for 3 min. The cell pellets were fixed with 4% paraformaldehyde (pH 7.4) for 24 h and subsequently routinely paraffin wax embedded. Sections (3–5 µm) were prepared and stained for reptarenavirus NP as previously described [10].

### 2.9. Statistical Analyses

Test agreement was examined using Cohen’s kappa (κ). Test sensitivity and specificity was calculated against the detection of inclusion bodies in blood or tissue samples. Stata 13 (StataCorpLP, College Station, TX, USA) was used for the analysis.

## 3. Results

### 3.1. Primer Cocktail and Optimization of the Cycling Conditions for the Multiplex RT-PCR Method

The “Multiplex RT-PCR primer mix” comprises six forward primers (F1–6) and seven reverse primers (R1–7), designed to target conserved regions in the reptarenavirus S segments, more specifically, an approximately 140 bp stretch within the open reading frame (ORF) of glycoprotein 2 (GP2). Table 1 shows the reptarenavirus S segments matching the primer sequences completely, together with those that align with the primer with one, two or three mismatches. Details on the exact position of the primers for each of the reptarenavirus S segment sequences that they are predicted to bind, including the information of S segments with single, double or triple mismatches to each primer, are provided in Table S1.

For the multiplex RT-PCR, we started by testing the Phusion Flash High-Fidelity PCR Master Mix (Thermo Fisher Scientific, Waltham, MA, USA) based on the described amplification protocol [6]. The optimization of the cycling conditions was done with a mix including primers F1–5 and R1–5 (0.5 µM or 0.25 µM each) listed in Table 1. During optimization, we varied annealing temperatures (between 58 °C and 63 °C), annealing times (between 1 and 5 s), extension times (between 2 and 7 s) and cycle numbers (38 vs. 42 cycles), with the aim to improve the intensity of specific bands and to reduce the background in agarose gel electrophoresis. 

Examples of results obtained using the same samples with different cycling conditions are provided in Figure S1. The optimized cycling conditions were: 1. initial denaturation at 98 °C for 10 s; 2. denaturation at 98 °C for 1 s; 3. annealing at 58 °C for 2 s (formerly 60 °C for 5 s); and 4. extension at 72 °C for 3 s (formerly 7 s); with steps 2 to 4 repeated 38 times until final extension at 72 °C for 1 min. 

### 3.2. Limit of Detection of the Multiplex RT-PCR Protocol for Specific Reptarenavirus S Segments and Optimization of Primer Concentrations

After setting up the cycling conditions for the multiplex RT-PCR primer mix, we aimed to improve the sensitivity of detection by altering the primer mix concentration. We first defined the detection limit for selected reptarenavirus S segments using virus isolates (ABV-1, UGV-1, UGV-2, UHV-1 and UHV-2) and in vitro-transcribed RNAs (TMSV-2, S2-like, S5-like, S7- and S10-like and UGV/S6-CR S segments). We determined the number of S segments per ml of each virus isolate using qRT-PCR and calculated the synthetic RNA copy numbers based on the RNA concentration. To define the detection limit, 10-fold serial dilutions of the cDNAs of each of the above S segment controls were amplified via multiplex RT-PCR at different primer mix concentrations, and the products were examined through agarose gel electrophoresis. The initial primer mix (F1–5 and R1–5) at 0.25 µM each produced a positive result with a very high input RNA amount for UHV-1 (10^4^–10^5^ copies/lane), S5-like (10^6^–10^7^ copies/lane) and UGV/S6-CR S segments (10^5^–10^6^ copies/lane) (Table S2). To improve the sensitivity, we tested the effect of increased concentrations of the primers with the highest homology for these S segments (F4 and R5 for UHV-1, F5 and R3 for S5-like, and F4 and R3 for UGV/S6-CR), or of increased concentrations of all primers. While both alterations substantially improved the UHV-1 S segment detection (10^2^–10^3^ copies/lane), the changes did not markedly improve the detection of S5-like and UGV/S6-CR S segments (Table S2). 

In parallel, we tested the multiplex RT-PCR protocol with different concentrations of the F1–5 and R1–5 primer mix on cDNAs produced from blood samples from an earlier study [9]. In addition, we tested UGV-1- and mock-infected cell culture lysates and UGV-1-infected cell supernatants as well as BIBD-negative snake tissue lysates (Table S3; Figure S2a–i). We varied the primer concentrations between 0.25 and 0.8 µM and tested concentrations up to 1 µM for primers F4, F5, R3 and R5. The blood samples known to contain high RNA levels produced a strong reaction in the multiplex RT-PCR irrespective of the primer concentrations, whereas samples containing low RNA levels required primer concentrations of 0.4 µM or higher (Table S3; Figure S2a–i). Blood and liver samples previously analyzed as reptarenavirus- and BIBD-negative consistently produced negative results; however, incrementing the primer concentrations above 0.5 µM increased the non-specific background (Table S3; Figure S2a–i). The cell culture samples produced clear results with all primer concentrations tested (Table S3; Figure S2a–i). In addition, the combination of snake samples (blood or liver) with known amounts (500 and 50,000 copies of S segments per lane) of the UGV-1 stock showed clear detection up to 500 UGV-1 S copies with the primers’ concentration starting from 0.4 µM (Table S3, Figure S2a–i). 

**Figure 1:**
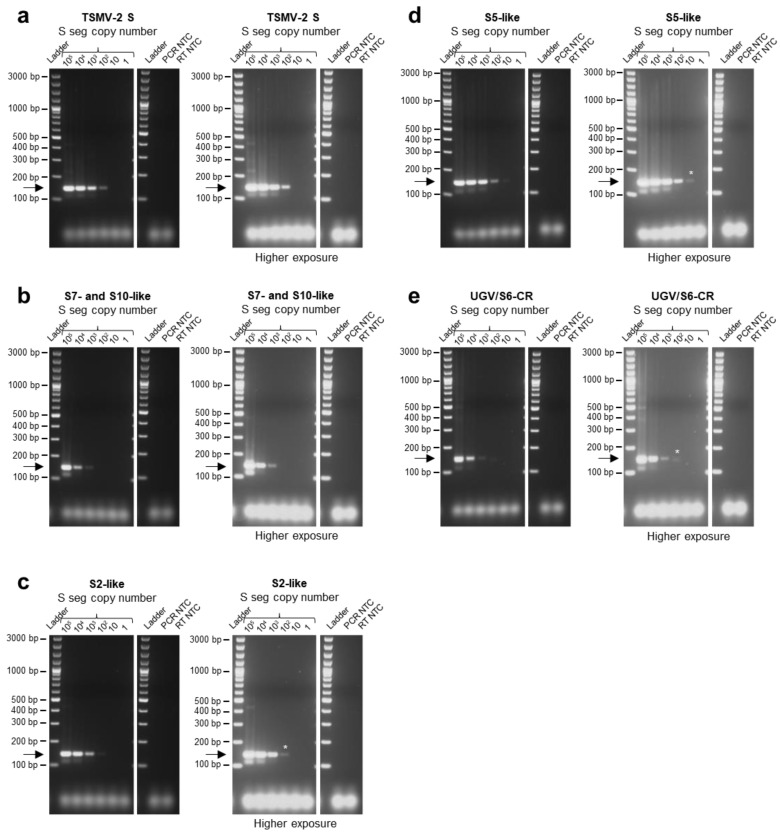
Determination of multiplex RT-PCR detection limits for specific reptarenavirus sequences using in vitro transcribed reptarenavirus S segment synthetic genes. The multiplex RT-PCR was performed on 10-fold dilution series of known copy numbers (range 2 × 10^5^–2 copies/reaction and 10^5^–1 copies/lane) of cDNA sequences obtained from in vitro transcription from a synthetic target portion of (**a**) Tavallinen suomalainen mies virus 2 (TSMV-2) S segment, (**b**) S7- and S10-like, (**c**) S2-like, (**d**) S5-like and (**e**) Costa Rica University of Giessen (UGV)/S6 S segment (UGV/S6-CR), expressed from the T7 promoter in a pUC57 vector. PCR products were separated by agarose gel electrophoresis and the bands visualized under UV light by GelRed nucleic acid staining (Biotium, Fremont, CA, USA) pre-cast to the gels. Specific amplicons are at approximately 140 bp and are indicated by an arrow. In each panel the images on the right represent the gels imaged at higher exposure times than on the left. Bands that became visible after higher exposure times are indicated by an *. For each reptarenavirus S segment, the detection limit of the multiplex RT-PCR method was determined by the highest dilution that yielded a visible specific band. Ladder: GeneRuler 100 bp plus DNA ladder (Thermo Fisher Scientific, Waltham, MA, USA); RT: reverse transcription, NTC: no template control, seg: segment.

**Figure 2:**
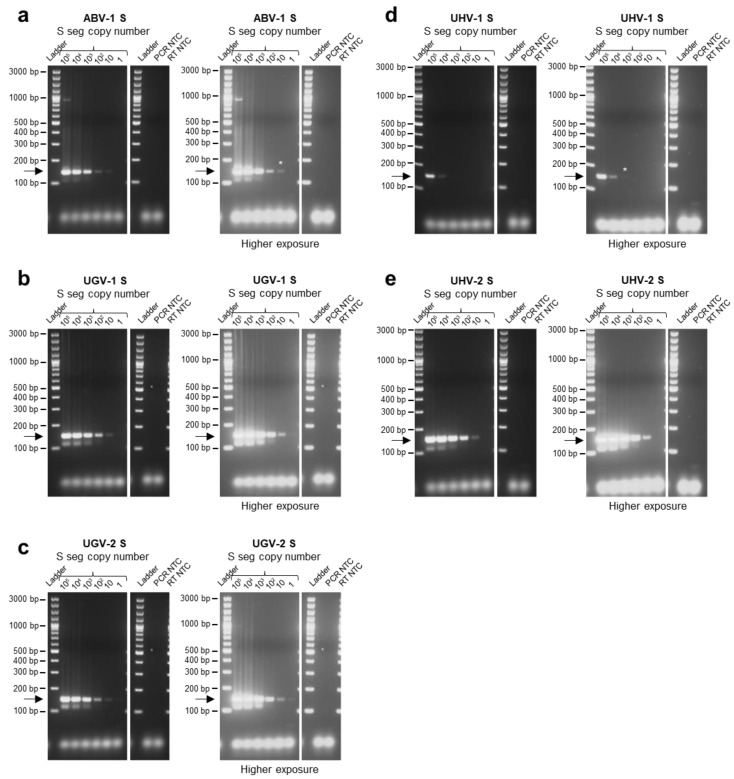
Determination of multiplex RT-PCR detection limit for specific reptarenavirus sequences using genomic RNA isolated from reptarenaviruses. cDNAs were synthesized from RNA genomes extracted from single reptarenavirus isolates collected in supernatants of the I/1Ki cell line inoculated with either Aurora borealis virus 1 (ABV-1), University of Giessen virus 1 (UGV-1), UGV-2, University of Helsinki virus 1 (UHV-1) or UHV-2. For each of the single isolates, the number of S segments per ml of cell culture supernatant was quantified through qRT-PCR analyses. The multiplex RT-PCR was performed on 10-fold dilution series of cDNA of (**a**) ABV-1, (**b**) UGV-1, (**c**) UGV-2, (**d**) UHV-1 and (**e**) UHV-2 with known S segment copy numbers (range 2 × 10^5^–2 copies/reaction and 10^5^–1 copies/lane). The PCR products were separated by agarose gel electrophoresis and the bands visualized under UV light by GelRed nucleic acid staining (Biotium, Fremont, CA, USA) pre-cast to the gels. Specific amplicons are at approximately 140 bp and are indicated by an arrow. In each panel, the images on the right represent the gels imaged at higher exposure times than on the left. Bands that became visible after higher exposure times are indicated by an *. For each reptarenavirus S segment, the detection limit of the multiplex RT-PCR method was determined by the highest dilution that yielded a visible specific band. Ladder: GeneRuler 100 bp plus DNA ladder (Thermo Fisher Scientific, Waltham, MA, USA), RT: reverse transcription, NTC: no template control, seg: segment.

Afterwards, with the aim to increase the sensitivity for S5-like and UGV/S6-CR S segments, we included specific primers for S5-like (R6) and UGV/S6-CR (F6 and R7) segments to the primer mix, which indeed helped to improve their detection limits: 10–10^2^ copies/lane for S5-like and 10^2^–10^3^ for UGV/S6-CR S segment. Increasing the concentration of all primers also improved the detection of S7- and S10-like, S2-like and ABV-1 S segments from 10^4^–10^5^ segments/lane for S7- and S10-like and 10^3^–10^4^ segments/lane for S2-like and ABV-1 S to 10^3^ for S7- and S10-like, 10^2^ for S2-like and 10 for the ABV-1 S segment (Table S2). After further optimization steps, we chose to use primers F1–5 and R1–6 at 0.4 µM and primers F6 and R7 at a 0.2 µM concentration (Figure 1 and Figure 2; Table S2). After protocol optimization, the detection limits of the multiplex RT-PCR for the tested S segments were: 10^2^ copies/lane for TMSV-2, 10^3^ for S7- and S10-like, 10^2^ for S2-like, 10–10^2^ for S5-like, 10^2^–10^3^ for UGV/S6-CR (Figure 1a–e; Table S2), 10 for ABV-1, UGV-1 and UGV-2, 10^3^–10^4^ for UHV-1 and 10 for UHV-2 (Figure 2a–e; Table S2). The corresponding detection limits for reptarenavirus S segment copies per µL of blood would hence be: 4000–40,000 for UHV-1, 4000 for S7- and S10-like, 400–4000 for UGV/S6-CR, 400 for TMSV-2 and S2-like, 40–400 for S5-like and 40 for ABV-1, UGV-1, UGV-2 and UHV-2 S segments. Importantly, no primer dimers, such as bands at approximately 50 bp, were observed with the final conditions of the protocol (Figure 1a–e and Figure 2a–e).

### 3.3. Validation of the Multiplex RT-PCR Protocol by Testing Its Reliability on Samples from Different Snake Collections

After setting up the conditions for the multiplex RT-PCR, we evaluated the test performance with archival blood samples collected from two snake collections for our earlier studies [7,9]. For both collections, we had already identified not only the snakes affected by BIBD (through the detection of IBs in blood cells in stained blood smears), but also the BIBD-free reptarenavirus carriers [7,9]. In both studies, we had used NGS to identify the reptarenavirus segments present in each collection. The samples of the first, smaller study included UGV-2, S5-like and TMSV-2 S segment-positive individuals [7], whereas the second larger study (183 snakes) confirmed infection based on the detection of the UGV/S6 S segment, the only S segment detected in this collection and for which we and others found strong evidence that it was prevalent in all snakes with BIBD in Europe and the USA [6,7,9,10,19,21,22]. 

The first study panel included 70 samples from one colony, comprising 34 BIBD-positive and 36 BIBD-negative snakes [7]. Of these, only four BIBD-negative animals appeared to be reptarenavirus S segment-free. Of the 66 reptarenavirus-infected animals, 30 carried three S segments, 32 carried two S segments and four carried one S segment (Table S4) [7]. For the present study, 64 of the 70 original blood samples were available for RNA extraction (Table S4). The multiplex RT-PCR produced a positive result for all the 34 samples collected from the BIBD-positive snakes (of which 23 had been shown to harbor three S segments, nine to carry two S segments and two to harbor one S segment; Table S4) [7]. For the 30 blood samples available from the BIBD-negative snakes, 26 had earlier been identified as reptarenavirus S segment-positive. The multiplex RT-PCR produced a strong positive result for 14 samples (of which three had been shown to carry three and eleven to carry two S segments), whereas weaker positive bands were obtained for the remaining 12 samples (of these, three had been identified to carry three, eight to carry two and one to carry a single S segment). The four samples previously identified as reptarenavirus-negative in single reptarenavirus RT-PCRs [7] produced a negative result also in the multiplex RT-PCR (Table S4). 

**Figure 3:**
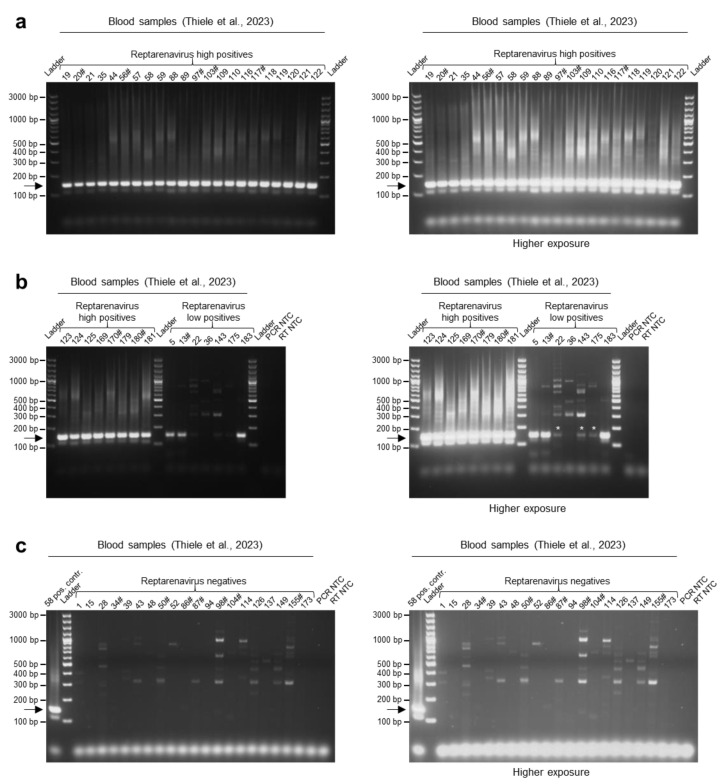
Testing the multiplex RT-PCR method on a snake collection with confirmed BIBD cases and reptarenavirus carriers [9]. The multiplex RT-PCR was applied to 181 of the originally 183 previously tested samples. (**a**) Results of the multiplex RT-PCR from blood samples of already known BIBD-positive and/or reptarenavirus-positive cases with high RNA levels (Thiele et al., 2023 [9]). (**b**) Results of the multiplex RT-PCR from blood samples of already known BIBD-positive and/or reptarenavirus-positive cases, either with high (>100,000 UGV/S6 S segment copies per ng of RNA; >1,000,000 UGV/S6 S segment copies/µL blood) or low (<500 UGV/S6 S segment copies per ng of RNA; <1,000 UGV/S6 S segment copies/µL blood) RNA levels, as determined by qRT-PCR for UGV/S6 S segment [9]. Note that sample no. 36, that here resulted as negative, gave a positive result for reptarenaviruses in a repeat PCR run with cDNA synthesized earlier. (**c**) Results of multiplex RT-PCR from blood samples of a portion of the BIBD- and reptarenavirus-negative animals, shown as examples. The multiplex RT-PCR results on the complete collection are reported in Table S5. The PCR products were separated by agarose gel electrophoresis and the bands visualized under UV light by GelRed nucleic acid staining (Biotium, Fremont, CA, USA) pre-cast to the gels. Specific amplicons are at approximately 140 bp and are indicated by an arrow. In each panel, the images on the right represent the gels imaged at higher exposure times than on the left. Bands that became visible after higher exposure times are indicated by an *. Ladder: GeneRuler 100 bp plus DNA ladder (Thermo Fisher Scientific, Waltham, MA, USA); RT: reverse transcription; NTC: no template control. #: samples analyzed via next-generation sequencing (NGS) and de novo assembly [9].

The second panel of samples originated from a larger breeding colony that comprised blood samples from 183 boa constrictors: 28 BIBD-positive and 155 BIBD-negative animals [9]. Of the 28 BIBD-positive samples, 27 contained high levels of UGV/S6 S segment RNA (>100,000 S segment copies per ng of RNA; >1,000,000 S segment copies/µL blood), while the remaining animal had a lower UGV/S6 S segment RNA level (<500 S segment copies per ng of RNA; < 1,000 S segment copies/µL blood) (Table S5). Of the BIBD-negative animals, nine harbored UGV/S6 S, three with high and six with low UGV/S6 S segment RNA loads (Table S5) [9]. For the present study, 181 of the 183 samples were available. All samples with a high UGV/S6 S segment RNA level, i.e., the 27 BIBD-positives and the 3 BIBD-negatives, gave clear positive bands with the multiplex RT-PCR approach (Figure 3a,b; Table S5). Of the seven samples with a low amount of UGV S segment RNA, one (no. 36) produced a negative result in the multiplex RT-PCR, which is likely due to the freezing/thawing-induced degradation of the low amount of RNA in this sample (as determined previously) (Figure 3b; Table S5). We subsequently re-tested this sample by multiplex RT-PCR, using cDNA synthesized earlier from the same RNA sample; this yielded a positive result, further supporting our interpretation. All samples formerly tested negative for UGV/S6 S segment produced a negative result in the multiplex RT-PCR (Table S5; examples from a subset of samples in Figure 3c). In conclusion, the multiplex RT-PCR results of both sample cohorts were in agreement with the results of the initial studies [7,9]. 

To further validate the multiplex RT-PCR results, we used some of the reptarenavirus S segment-positive and -negative blood samples of the second study [9] to inoculate I/1Ki cells known to be highly permissive for reptarenaviruses [3,10,24,27]. We selected the blood samples of two BIBD-positive animals (nos. 21 and 56, the latter had the highest UGV/S6 S segment load per ng of RNA), one BIBD-negative reptarenavirus carrier with high (no. 109) and five with low UGV/S6 S segment levels (nos. 22, 36, 143, 175 and 183), as well as two BIBD- and reptarenavirus-negative samples (nos. 52 and 155) for the isolation attempt. We collected supernatants from the I/1Ki cell cultures inoculated with each of the blood samples at 6 dpi and at 18 dpi, extracted RNA from the supernatants and subjected it to multiplex RT-PCR. In parallel, we collected the cells at the same time points and pelleted and processed them for immunocytochemistry to detect reptarenavirus NP expression. The multiplex RT-PCR yielded a positive result only for supernatants from cells inoculated with blood samples that had a high UGV S segment RNA level (nos. 21, 56 and 109) (Figure S3a,b), the same applied to viral NP expression in cell pellets prepared from the cultures (Figure S3c). The tested blood samples had been frozen and thawed several times prior to inoculation, which has likely substantially reduced their infectivity. Seeing a positive result in the multiplex RT-PCR for the blood samples from which the virus could not be isolated anymore indicates its robustness for the detection of reptarenavirus infection in snake blood samples that were stored sub-optimally or for prolonged time periods.

**Figure 4:**
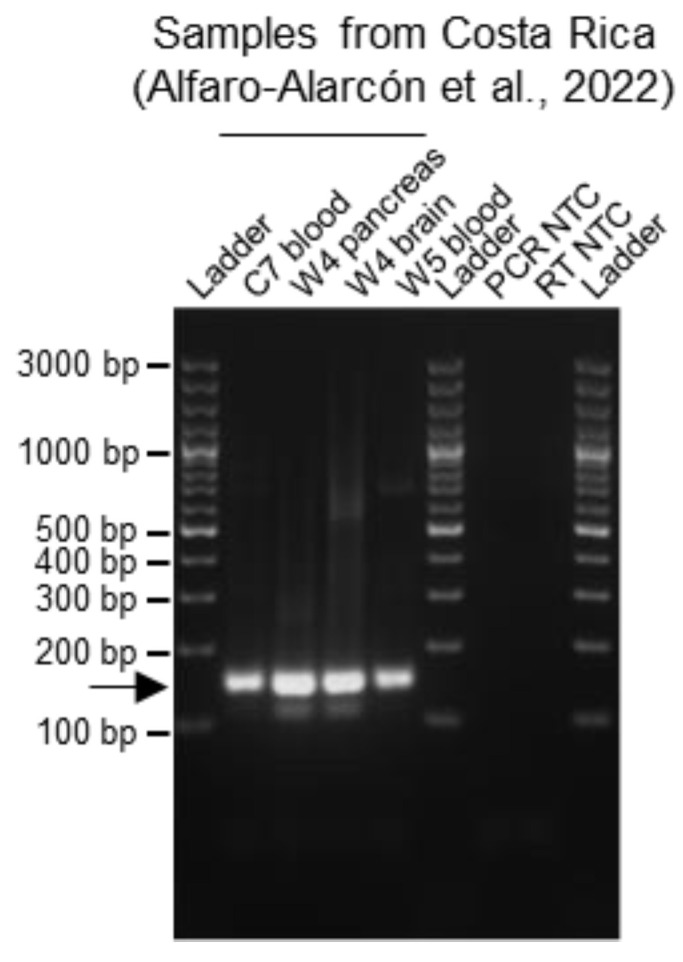
Testing the multiplex RT-PCR method on BIBD-positive indigenous snakes from Costa Rica. The multiplex RT-PCR method was performed on cDNA obtained from one captive (C7: blood) and two wild (W4: pancreas and brain tissue; W5: blood) snakes from Costa Rica which had been morphologically (histology and immunohistology for viral NP) diagnosed with BIBD and whose reptarenaviromes had been characterized via NGS (Alfaro-Alarcón et al., 2022 [10]). The RNA concentrations of the samples were as follows: 2.3 ng/µL for C7; 311 and 3.1 ng/µL for W4 pancreas and brain, respectively; and 22.4 ng/µL for W5. The multiplex RT-PCR yielded a positive result for all tested samples. PCR products were separated by agarose gel electrophoresis and the bands visualized under UV light by GelRed nucleic acid staining (Biotium, Fremont, CA, USA) pre-cast to the gel. Specific amplicons are at approximately 140 bp and are indicated by an arrow. Ladder: GeneRuler 100 bp plus DNA ladder (Thermo Fisher Scientific, Waltham, MA, USA); RT: reverse transcription; NTC: no template control.

Given that we optimized the multiplex RT-PCR detection for UGV/S6-CR S sequences (Figure 1e; Table 1 and Table S2), we additionally tested the reliability of the multiplex RT-PCR approach on previously characterized samples from confirmed indigenous BIBD-positive snakes from Costa Rica [10]. In fact, although the S segment found in these animals represents the same species as the UGV/S6 S found in snakes in both Europe and the US [21,22], it is a novel variant and the sequence variability in the region chosen for primer design was such that the primers designed for UGV/S6 S would not allow the detection of UGV/S6-CR S with sufficient sensitivity. Therefore, we added primers specific for UGV/S6-CR S to the multiplex RT-PCR primer cocktail. Specifically, we analyzed one captive (C7) and two wild (W4 and W5) boid snakes with reptarenavirus infection and BIBD confirmed by combined histology, immunohistology (viral NP), transmission electron microscopy and NGS examinations [10]. For all three tested individuals, the multiplex RT-PCR detected the reptarenavirus infection, either in the blood samples (C7 and W5) or, as blood was not available, in brain and pancreas tissue samples (W4) (Figure 4). 

In a final step, we wanted to challenge the multiplex RT-PCR on random diagnostic samples and tested 97 blood samples from boa constrictors of different ages from a total of 11 different privately owned snake colonies with previous confirmed BIBD cases (Table S6). The multiplex RT-PCR screening confirmed a reptarenavirus infection in all 24 boas found positive for BIBD and detected three additional reptarenavirus carriers (Table S6). 

A statistical analysis was undertaken on all the material included in this study (347 snakes). This identified almost perfect agreement between the results of the multiplex RT-PCR and those of the qRT-PCR for UGV/S6 (Cohen’s κ = 0.991, *n* = 245, 95%CI: 0.975–1.000). Table S7a summarizes the agreement between all the tests included in the study (including those from [9]). The agreement between the multiplex RT-PCR and the detection of IB (gold standard for the diagnosis of BIBD) is substantial (Cohen’s κ = 0.740, 95%CI: 0.665–0.814) but not significantly different from the agreement of UGV/S6 qRT-PCR with the detection of IB (Cohen’s κ = 0.6878, 95%CI: 0.524–0.852).

The sensitivity and specificity of the multiplex RT-PCR and the qRT-PCR for UGV/S6 were examined against the detection of IB. Table S7b summarizes the results of these tests. In addition, the two RT-PCR tests were compared with regards to their sensitivity and specificity using the UGV/S6 test as a provisional gold standard, since it predates the multiplex RT-PCR (Table S7c). The proportion of IB-positive animals that tested positive with the multiplex RT-PCR (sensitivity) is 100% (95%CI: 100–100%). The proportion of BIBD-negative animals that tested negative with the multiplex RT-PCR (specificity) is 85% (95%CI: 81.24–88.76%). The sensitivity of the qRT-PCR for UGV/S6 S segment is also 100% (95%CI: 100–100%) and the specificity is 78.53% (95%CI: 73.47–83.59%). The probability of an animal identified as positive with the multiplex RT-PCR also being positive for IBs (positive predictive value/PPV) is 69.05% (95%CI: 64.18–73.91%), when the BIBD prevalence is 25.07% (95%CI: 20.51–29.635). The negative predictive value (NPV) is 100% (95%CI: 100–100%). 

### 3.4. Improvement of Data Quality by DNase Treatment during the RNA Extraction Procedure from Blood Samples

**Figure 5:**
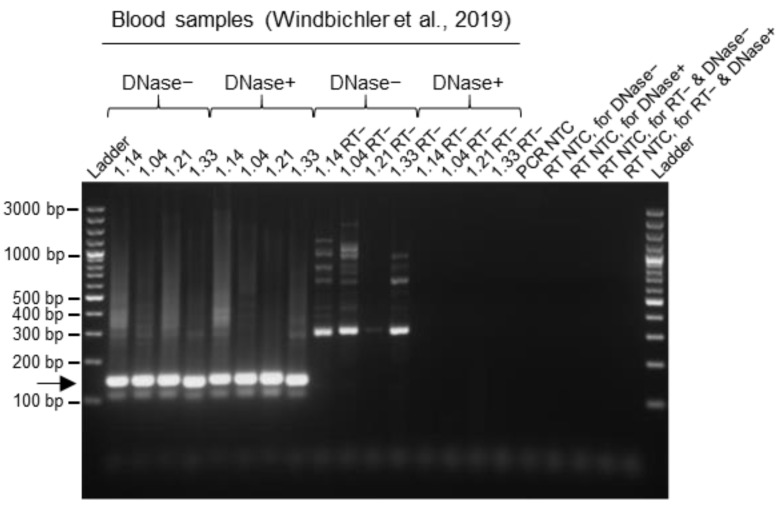
Effect of DNase treatment during RNA extraction from snake blood on the multiplex RT-PCR results. RNA was isolated from four BIBD-positive blood samples from the first study (Windbichler et al., 2019 [7]), with (DNase+) or without (DNase−) a DNase digestion step, taken as examples. For each sample, the subsequent reverse transcription was performed either including the reverse transcriptase enzyme within the reaction or without it, as reverse transcriptase minus (RT−) negative control, to assess for genomic DNA contamination of the RNA sample. The introduction of a DNase digestion step led to a decrease of the non-specific background without affecting the sensitivity of the multiplex RT-PCR. The PCR products were separated by agarose gel electrophoresis and the bands visualized under UV light by GelRed nucleic acid staining (Biotium, Fremont, CA, USA) pre-cast to the gel. Specific amplicons are at approximately 140 bp and are indicated by an arrow. Ladder: GeneRuler 100 bp plus DNA ladder (Thermo Fisher Scientific, Waltham, MA, USA); RT: reverse transcription; NTC: no template control.

The RNA extraction procedure that we used for blood samples in earlier studies [9,10] occasionally yielded a non-specific background signal in the gel electrophoresis following the multiplex RT-PCR amplification, especially with reptarenavirus-negative samples and positive samples with low reptarenavirus RNA levels (Figure 3b,c). Assuming that this non-specific signal originated from genomic DNA, we added a DNase treatment step during the column-based RNA purification. We isolated RNA from four BIBD-positive blood samples from the first study [7] with and without the introduction of a DNase digestion step. The gel electrophoresis of the multiplex RT-PCR products from the cDNAs generated with and without the presence of reverse transcriptase (RT−) showed that the introduction of DNase digestion resulted in a clear reduction (or complete disappearance) of the non-specific products/bands, suggesting that the non-specific signal indeed originated from genomic DNA (Figure 5). Moreover, the intensities of specific bands from cDNA samples obtained from RNA extraction with DNase treatment were comparable to those obtained from the standard extraction approach without the DNase step, indicating that DNase digestion did not compromise the RNA yield (Figure 5). To conclude, we found that DNase digestion during RNA isolation improved the output clarity of the multiplex RT-PCR method. 

## 4. Discussion

Here, we describe a novel multiplex RT-PCR approach for the molecular diagnosis of reptarenavirus infection from blood samples. This approach was taken to overcome the main challenge, i.e., the high genetic variability of the genus *Reptarenavirus*, for a sensitive, reliable diagnostic test [6,7,9,10,18,21,22]. Earlier studies by others and us have attempted to tackle this problem by using degenerate primers [21] or by generating a colony-specific diagnostic tool employing NGS and genome de novo assembly [6,7,9]. The first approach did not work in our study and we found the latter procedure too expensive for routine diagnosis, e.g., the screening of larger snake colonies, and not practicable for individual samples. This motivated us to perform the present study. 

Reptarenaviruses have a bisegmented genome, hence the detection of either S or L segments is sufficient for diagnostic purposes. Since the number of known reptarenavirus L segments is higher than the number of known S segments [6,9,10,18,21,22], it is pragmatic to focus on a tool to detect all S segments. Interestingly, the most conserved region of the reptarenavirus genome lies in the GP2 ORF, which is located in the S segment. We thus designed a set of primers targeting the conserved region of the known S segments and evaluated the performance of the multiplex RT-PCR using samples from our earlier studies [7,9,10]. 

Particular emphasis was laid on the reliable recognition of those reptarenavirus species that had been most prevalent in different captive collections [6,7,9,21,22] and viruses identified in wild and captive indigenous Costa Rican snakes [10], from where UGV/S6 S segments identified in captive snakes in Europe and the US might have originated. We therefore used UGV/S6 [6,7,9,21,22], Costa Rica UGV/S6 [10], TSMV-2, S5/S5-like and ABV-2 S segments [6,7,21] as the references for primer design. The selected primers also provide a 100% match to several additional S segments identified in North American snake collections, namely S7, S8, S9 and S10 [21]. Through the titration of the template amount, we defined the limit of detection of the multiplex RT-PCR to be 10–100 copies/reaction for UGV-1, UGV-2, ABV-1 and -2, TSMV-2, S5-like and UGV/S6-CR S segments, which are identical to some of the multiplex RT-PCR primers. Analyses using samples containing the UHV-1, UHV-2, S2-like and S7- and S10-like S segments, which show mismatches to the primers, indicated that the multiplex RT-PCR produced a positive result with between 10 and 10^3^–10^4^ copies/lanes for the respective S segments, showing, generally, a reduced sensitivity in comparison to the sequences fully matching some of the primer sequences. 

After optimizing the multiplex RT-PCR protocol, we validated the test performance with RNA extracted from diagnostic blood samples collected in defined breeding colonies and analyzed in earlier studies [7,9]. For both sets of samples, the multiplex RT-PCR produced results identical to those of the original studies [7,9]. Only one sample, with known very low RNA levels [9], initially yielded a negative result, but when it was re-tested via multiplex RT-PCR with a formerly synthesized cDNA from the same RNA sample, it resulted as reptarenavirus-positive. This indicated that the little RNA present in the sample had degraded during the freeze–thaw cycles. To conclude, the sensitivity and specificity of the multiplex RT-PCR appears to be relatively close to our qRT-PCR approach. 

Using a subset of blood samples with high UGV/S6 S titers, we could propagate reptarenaviruses in the kidney-derived I/1Ki permanent boid cell line, which is highly permissive to reptarenavirus infection [3,10,24,27]. In fact, we could clearly show via multiplex RT-PCR the specific reptarenavirus detection at both 6 and 18 dpi from cell culture supernatants collected from I/1Ki inoculated with blood samples with high reptarenavirus RNA concentrations. However, our attempts at isolating reptarenaviruses from samples with a low reptarenavirus RNA amount were unsuccessful. We speculate this to be the consequence of virus inactivation due to the repeated freezing and thawing of the blood samples (e.g., due to the release of proteases or aggregation). Further studies utilizing freshly collected or rapidly frozen blood samples would be required to estimate the correlation of the RNA amount and infectivity. 

We further confirmed the validity of the multiplex RT-PCR by applying it to blood and tissue samples from BIBD-affected wild and captive indigenous boas from Costa Rica where we have recently identified a novel UGV/S6 S variant [10]. Moreover, during the optimization of the method, we observed non-specific bands in the gel electrophoresis of the amplicons from some blood samples. These were particularly prominent in some of the negative samples and in samples with a low amount of reptarenavirus RNA. Similar non-specific bands appeared when amplicons from post mortem samples of different tissues, e.g., liver, were examined. The number and intensity of these bands was substantially reduced when a DNase treatment step was added to the RNA extraction protocol, indicating the removal of genomic DNA. We therefore consider this additional step as essential to identify animals with a low reptarenavirus RNA load. 

When we applied the multiplex RT-PCR to random diagnostic blood samples where only the BIBD status was known (the presence of IBs in blood cells in a cytological specimen from a blood smear), we detected reptarenavirus RNA in all BIBD-positive samples and identified three BIBD-free carriers. Given the determined limits of detection of the method, these results suggest that the multiplex-RT-PCR can also be applied to individual samples from variable sources. It could, therefore, also serve for further studies into the pathogenesis of BIBD, e.g., to decipher the processes that might drive IB formation and progression to clinical disease in reptarenavirus-infected snakes. 

The statistical analysis for test sensitivity and specificity has limitations. The presence of IB is the perceived gold standard for clinical diagnosis while both RT-PCR tests detect genomic material. There is no currently established gold standard and though the multiplex RT-PCR is promising, further work is needed to fully evaluate its sensitivity and specificity [28]. 

## 5. Conclusions 

We have developed a multiplex RT-PCR method that simultaneously targets different reptarenavirus S segment genotypes. A comparison with results from earlier studies indicates that the method has a similar sensitivity as virus-specific RT-PCR and qRT-PCR systems. The method has the advantage that it is affordable, cost-effective and can be performed without qPCR instrumentation. Provided appropriate sensitivity can be achieved, it may represent a tool applicable for routine diagnostics and the large-scale screening of snake colonies, as in our hands it allows the reliable identification of reptarenavirus infection also in clinically healthy virus carriers. However, from the validation steps it is evident that reliable results can only be expected from samples of an adequate RNA concentration (≥5 ng/µL) and RNA quality. Before the implementation of the described method as a diagnostic tool, the specificity must be established using proper reptarenavirus-negative and -positive controls. A limitation of the method is that it does not provide any information on the genotype and quantity of the reptarenavirus S segments present in a blood sample. 

## Figures and Tables

**Table 1:** List of primers forming the multiplex RT-PCR primer mix. The length and melting temperature (T_m_) relative to the concentration applied with the enzyme used (Phusion Flash polymerase) are listed for each primer. The reptarenavirus S segments expected to bind each primer are reported and classified according to full alignment with either 100% identity or 1, 2 or 3 mismatches. The mismatches located in the last four nucleotides (nt) at the 3′ end of each primer were not considered. Further details on the position of each primer relative to the listed reptarenavirus target sequences were determined using BLAST (https://blast.ncbi.nlm.nih.gov/Blast.cgi (accessed on 16 November 2023), National Library of Medicine, NCBI, Bethesda, MD, USA) and are available in Table S1.

Primers					Reptarenavirus S Segment Sequences Expected to be Bound by the Primers			
Name	Sequence	Length (nt)	Final Concentration (µM)	T_m_	100% Identity	1 Mismatch	2 Mismatches	3 Mismatches
F1	5′-GTGCAGGCATAACCAATTCAC-3′	21	0.4	62.8	UGV-1-4/S6, S6A, S7, S8, S9, S10	ABV-2, other UGV-1-4/S6, S6B, S7-like, other S9, S10-like, UGV/S6-CR-wild4	UHV-2, other UGV-1-4/S6, S2, S2-like, S4, GGV, NL-3	UHV-3, PAV-1
F2	5′-GTGCAGGCATAACAAATTCAC-3′	21	0.4	61.0	S7-like, S10-like	UGV-1-4/S6, S6A, GGV, S2, S2-like, S4, S7, S8, S9, S10	PAV-1, UHV-2, other UGV-1-4/S6, S6B, ABV-2, NL-3, other S9, UGV/S6-CR	Other UGV-1-4/S6
F3	5′-CAGTGCTGGAATAACTAATTCTC-3′	23	0.4	59.8	S11			
F4	5′-GTGCAGGCATAACCAACTC-3′	19	0.4	61.5	TSMV-2, ArBV-1, ABV-2, UGV-1-4/S6, S9, UGV/S6-CR-wild4	Other ArBV-1, S2, S2-like, S3, S4, GGV, NL-3, ABV-1, other UGV-1-4/S6	PAV-1, UHV-1	
F5	5′-GTGTTGGCATCACAAACTC-3′	19	0.4	59.8	S5-like, S5		PAV-1	S2, S2-like, S4, GGV
F6	5′-GCGCAGGCATTACCAATTC-3′	19	0.2	61.4	UGV/S6-CR		UGV-1-4/S6, S6A, S7, S8, S9, S10	Other UGV1-4/S6, S7-like, S10-like, UHV-2
R1	5′-CATGATCTCTAATTTCTCCTTCTTC-3′	25	0.4	59.9	UGV-1-4/S6	ABV-2, other UGV-1-4/S6, S6A, S7, S8, ArBV-1, UGV/S6-CR-wild4	Other ABV-2, TSMV-2, PAV-1, GGV, other ArBV-1, S2, S3	ABV-1, NL-3, other S2, S2-like, S4
R2	5′-GATCTCTGATTTCACCTTCTTCC-3′	23	0.4	61.4	PAV-1, ABV-2, TSMV-2, GGV, S2	other S2, S2-like, ABV-1, other ABV-2, NL-3, S4		
R3	5′-TCTCTGATCTCACCTTCCTC-3′	20	0.4	60.6	S6B, S9, S10	S11, ArBV-1	Other ArBV-1, S5, S5-like, UGV/S6-CR, UGV-1-4/S6	UHV-2, UHV-3
R4	5′-ACATGATCTCTAATCTCTCCTTC-3′	23	0.4	59.7	UGV-1-4/S6, S6A, S7, S8, UGV/S6-CR-wild4	Other UGV-1-4/S6, S7-like, S10-like	Other UGV-1-4/S6, ABV-1, ABV-2, ArBV-1, S2, S2-like, S4, S11	Other ABV-2, other ArBV-1, GGV, PAV-1, other S2, S3, TMSV-2
R5	5′-ATGACATGATCCCTAATCTCAC-3′	22	0.4	60.1	UHV-2	UHV-3	UHV-1, ABV-1, S11	ArBV-1, ABV-2, GGV
R6	5′-GGTCTCTTATTTCACCTTCCTC-3′	22	0.4	60.8	S5-like, S5		ArBV-1, S6B, S9, S10, S11	S7-like, S10-like
R7	5′-ATGACATGATCTCTGATCTCG-3′	21	0.2	58.7	UGV/S6-CR			

Abbreviations: F: forward; R: reverse; ABV-1: Aurora borealis virus 1; ABV-2: Aurora borealis virus 2; ArBV-1: Aramboia boa virus 1; GGV: Golden Gate virus; PAV-1: Porto Alegre virus 1; NL-3: arenavirus NL isolate 3; TSMV-2: Tavallinen suomalainen mies virus 2; UGV-1-4/S6: University of Giessen virus 1-4/S6; UGV/S6-CR: University of Giessen virus/S6 from Costa Rica; UHV-1: University of Helsinki virus 1; UHV-2: University of Helsinki virus 2; UHV-3: University of Helsinki virus 3.

## Data Availability

Data are contained within the article and supplementary materials.

## Supplementary Materials

The following supporting information can be downloaded at: https://www.mdpi.com/article/10.3390/v15122313/s1. Figure S1: Testing the multiplex RT-PCR protocol with different cycling conditions; Figure S2: Testing the multiplex RT-PCR protocol with different primer concentrations; Figure S3: Checking the multiplex RT-PCR results by transferring the reptarenavirus infection from snake blood samples to a cell culture model; Table S1: List of reptarenavirus S segments expected to be bound by the primers of the multiplex RT-PCR primer mix; Table S2: Limit of detection of the multiplex RT-PCR method for specific reptarenavirus S segment sequences; Table S3: Optimization of primer concentrations of the multiplex RT-PCR, on diagnostic and cell culture material, for reptarenavirus detection; Table S4: Multiplex RT-PCR results are in agreement with the previous detection of reptarenavirus infection in the first whole captive snake collection under analysis; Table S5: Multiplex RT-PCR results are in agreement with the former detection of reptarenavirus infection in a subset of animals belonging to the second entire captive snake collection under analysis; Table S6: Multiplex RT-PCR results on different snake breeding colonies with BIBD-positive cases; Table S7: Agreement between tests and sensitivity and specificity analysis for the multiplex RT-PCR.
