# Supplementary material for: A Multiplex RT-PCR Method for the Detection of Reptarenavirus Infection
Source: Viruses. 2023 Nov 25;15(12):2313. doi: 10.3390/v15122313 (PMC10747477; doi:10.3390/v15122313)
Supplement: Supplementary file 1 [file viruses-15-02313-s001.zip › Supplementary Material/Main and Supplementary Figures.pptx]

## Slide 1
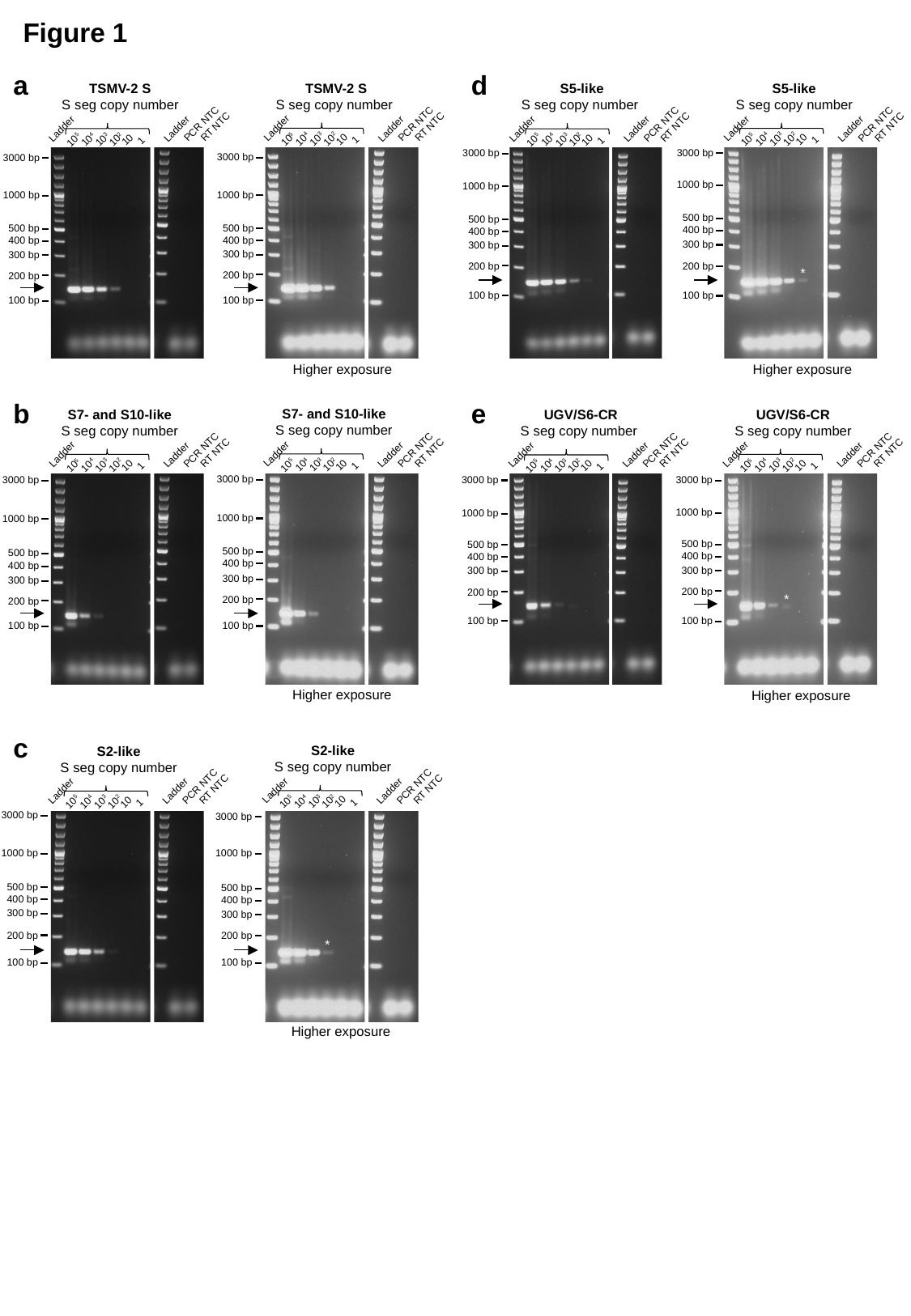

Figure 1
a
d
TSMV-2 S
S seg copy number
S5-like
S seg copy number
TSMV-2 S
S seg copy number
S5-like
S seg copy number
PCR NTC
PCR NTC
PCR NTC
PCR NTC
RT NTC
RT NTC
RT NTC
RT NTC
Ladder
Ladder
Ladder
Ladder
Ladder
Ladder
Ladder
Ladder
102
104
103
102
104
103
105
102
104
10
105
103
102
104
10
103
105
10
105
10
1
1
1
1
3000 bp
3000 bp
3000 bp
3000 bp
1000 bp
1000 bp
1000 bp
1000 bp
500 bp
500 bp
500 bp
500 bp
400 bp
400 bp
400 bp
400 bp
300 bp
300 bp
300 bp
300 bp
200 bp
200 bp
*
200 bp
200 bp
100 bp
100 bp
100 bp
100 bp
Higher exposure
Higher exposure
b
e
S7- and S10-like
S seg copy number
S7- and S10-like
S seg copy number
UGV/S6-CR
S seg copy number
UGV/S6-CR
S seg copy number
PCR NTC
PCR NTC
PCR NTC
PCR NTC
RT NTC
RT NTC
RT NTC
RT NTC
Ladder
Ladder
Ladder
Ladder
Ladder
Ladder
Ladder
Ladder
102
104
103
105
102
104
102
10
103
104
103
105
105
102
10
104
10
103
1
105
10
1
1
1
3000 bp
3000 bp
3000 bp
3000 bp
1000 bp
1000 bp
1000 bp
1000 bp
500 bp
500 bp
500 bp
500 bp
400 bp
400 bp
400 bp
400 bp
300 bp
300 bp
300 bp
300 bp
200 bp
200 bp
*
200 bp
200 bp
100 bp
100 bp
100 bp
100 bp
Higher exposure
Higher exposure
c
S2-like
S seg copy number
S2-like
S seg copy number
PCR NTC
PCR NTC
RT NTC
RT NTC
Ladder
Ladder
Ladder
Ladder
102
104
103
105
102
104
10
103
105
10
1
1
3000 bp
3000 bp
1000 bp
1000 bp
500 bp
500 bp
400 bp
400 bp
300 bp
300 bp
200 bp
200 bp
*
100 bp
100 bp
Higher exposure

## Slide 2
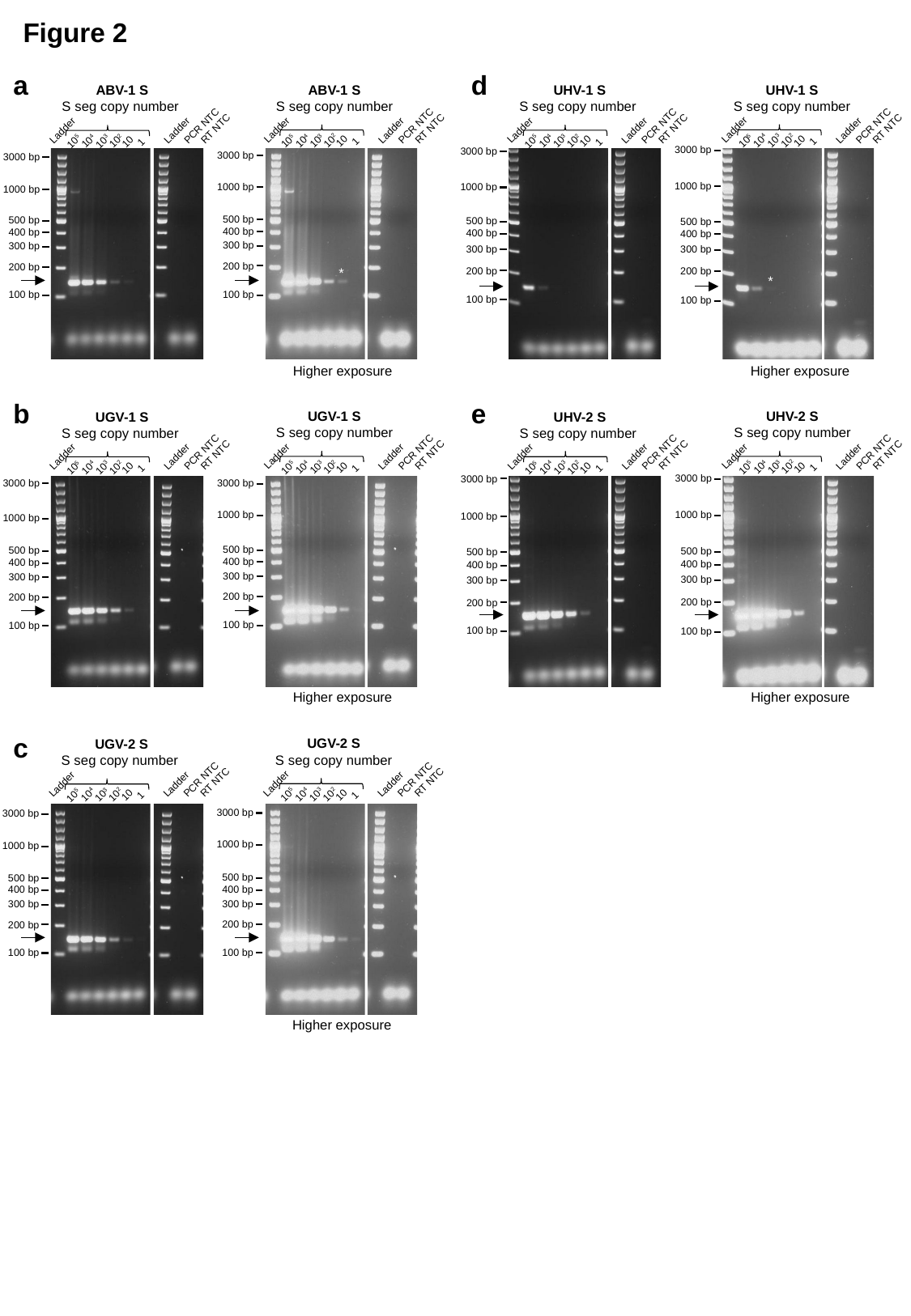

Figure 2
a
d
UHV-1 S
S seg copy number
ABV-1 S
S seg copy number
UHV-1 S
S seg copy number
ABV-1 S
S seg copy number
PCR NTC
PCR NTC
PCR NTC
PCR NTC
RT NTC
RT NTC
RT NTC
RT NTC
Ladder
Ladder
Ladder
Ladder
Ladder
Ladder
Ladder
Ladder
102
104
103
102
104
103
105
102
104
10
103
105
102
104
10
103
105
10
105
10
1
1
1
1
3000 bp
3000 bp
3000 bp
3000 bp
1000 bp
1000 bp
1000 bp
1000 bp
500 bp
500 bp
500 bp
500 bp
400 bp
400 bp
400 bp
400 bp
300 bp
300 bp
300 bp
300 bp
200 bp
200 bp
200 bp
*
200 bp
*
100 bp
100 bp
100 bp
100 bp
Higher exposure
Higher exposure
b
e
UHV-2 S
S seg copy number
UGV-1 S
S seg copy number
UHV-2 S
S seg copy number
UGV-1 S
S seg copy number
PCR NTC
PCR NTC
PCR NTC
PCR NTC
RT NTC
RT NTC
RT NTC
RT NTC
Ladder
Ladder
Ladder
Ladder
Ladder
Ladder
Ladder
Ladder
102
104
102
103
104
103
105
102
105
104
10
102
103
104
10
103
105
105
10
10
1
1
1
1
3000 bp
3000 bp
3000 bp
3000 bp
1000 bp
1000 bp
1000 bp
1000 bp
500 bp
500 bp
500 bp
500 bp
400 bp
400 bp
400 bp
400 bp
300 bp
300 bp
300 bp
300 bp
200 bp
200 bp
200 bp
200 bp
100 bp
100 bp
100 bp
100 bp
Higher exposure
Higher exposure
c
UGV-2 S
S seg copy number
UGV-2 S
S seg copy number
PCR NTC
PCR NTC
RT NTC
RT NTC
Ladder
Ladder
Ladder
Ladder
102
104
103
105
102
104
10
103
105
10
1
1
3000 bp
3000 bp
1000 bp
1000 bp
500 bp
500 bp
400 bp
400 bp
300 bp
300 bp
200 bp
200 bp
100 bp
100 bp
Higher exposure

## Slide 3
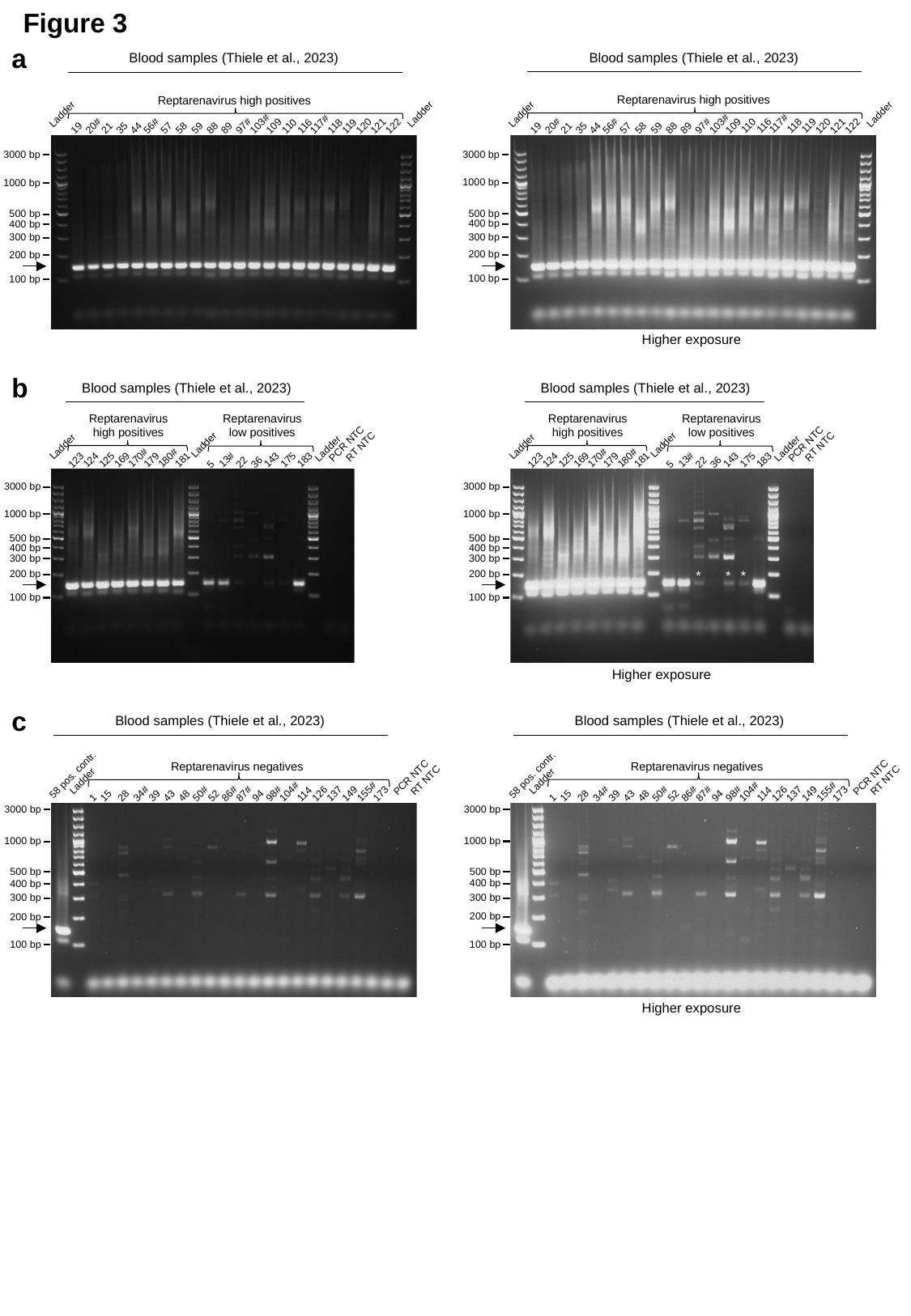

Figure 3
a
Blood samples (Thiele et al., 2023)
Blood samples (Thiele et al., 2023)
Reptarenavirus high positives
Reptarenavirus high positives
Ladder
Ladder
Ladder
Ladder
103#
117#
103#
117#
20#
56#
97#
109
110
116
118
119
120
121
122
20#
56#
97#
109
110
116
118
119
120
121
122
19
21
35
44
57
58
59
88
89
19
21
35
44
57
58
59
88
89
3000 bp
3000 bp
1000 bp
1000 bp
500 bp
500 bp
400 bp
400 bp
300 bp
300 bp
200 bp
200 bp
100 bp
100 bp
Higher exposure
b
Blood samples (Thiele et al., 2023)
Blood samples (Thiele et al., 2023)
Reptarenavirus high positives
Reptarenavirus low positives
Reptarenavirus high positives
Reptarenavirus low positives
PCR NTC
PCR NTC
Ladder
Ladder
RT NTC
RT NTC
Ladder
Ladder
Ladder
Ladder
170#
180#
170#
180#
124
125
169
179
181
143
175
183
124
125
169
179
181
13#
143
175
183
13#
123
123
22
36
22
36
5
5
3000 bp
3000 bp
1000 bp
1000 bp
500 bp
500 bp
400 bp
400 bp
300 bp
300 bp
200 bp
*
*
*
200 bp
100 bp
100 bp
Higher exposure
c
Blood samples (Thiele et al., 2023)
Blood samples (Thiele et al., 2023)
Reptarenavirus negatives
Reptarenavirus negatives
58 pos. contr.
58 pos. contr.
PCR NTC
PCR NTC
RT NTC
RT NTC
Ladder
Ladder
104#
155#
104#
155#
114
126
137
149
173
34#
50#
86#
87#
98#
114
126
137
149
173
34#
50#
86#
87#
98#
15
28
39
43
48
52
94
15
28
39
43
48
52
94
1
1
3000 bp
3000 bp
1000 bp
1000 bp
500 bp
500 bp
400 bp
400 bp
300 bp
300 bp
200 bp
200 bp
100 bp
100 bp
Higher exposure

## Slide 4
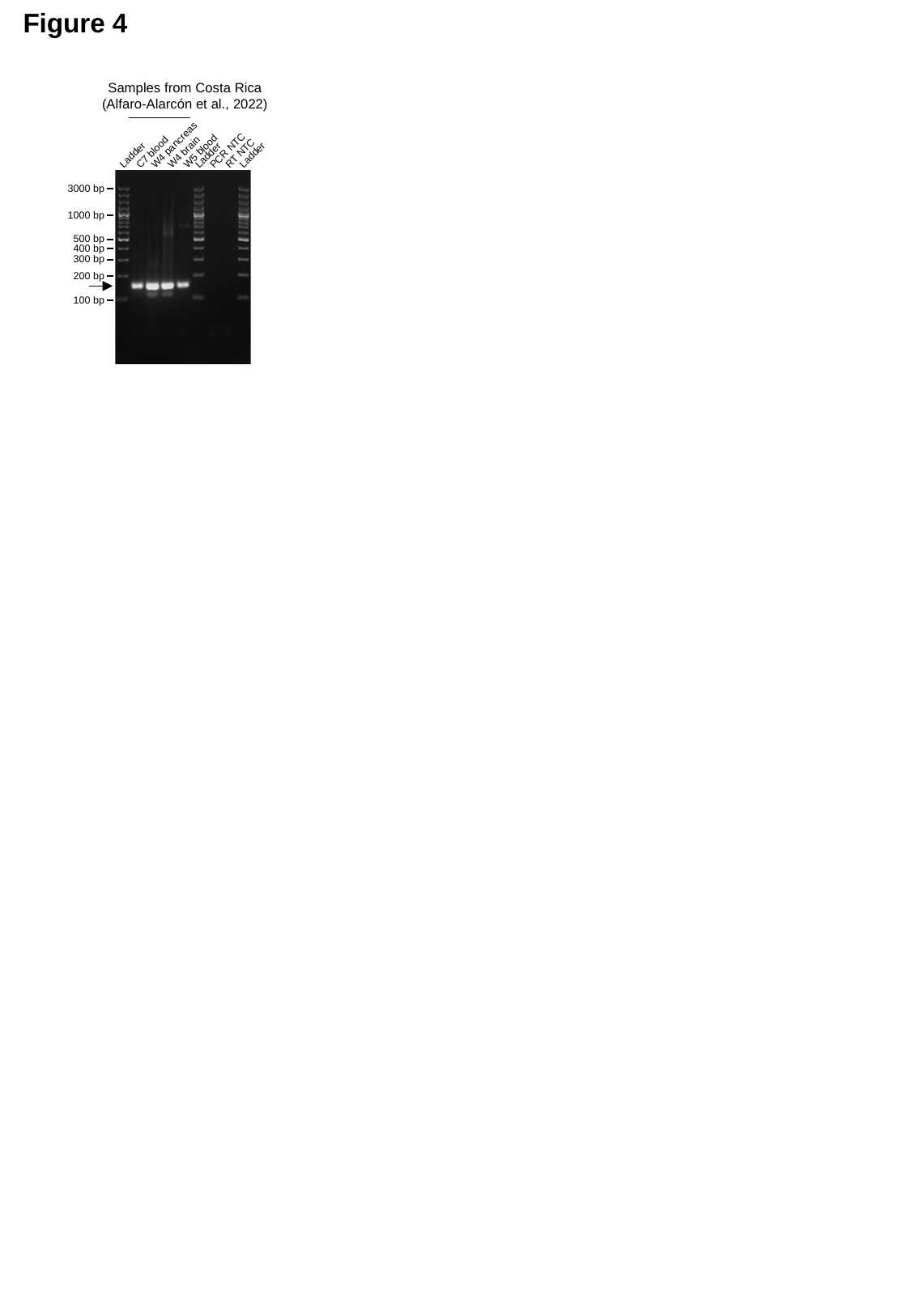

Figure 4
Samples from Costa Rica (Alfaro-Alarcón et al., 2022)
W4 pancreas
PCR NTC
W5 blood
C7 blood
W4 brain
RT NTC
Ladder
Ladder
Ladder
3000 bp
1000 bp
500 bp
400 bp
300 bp
200 bp
100 bp

## Slide 5
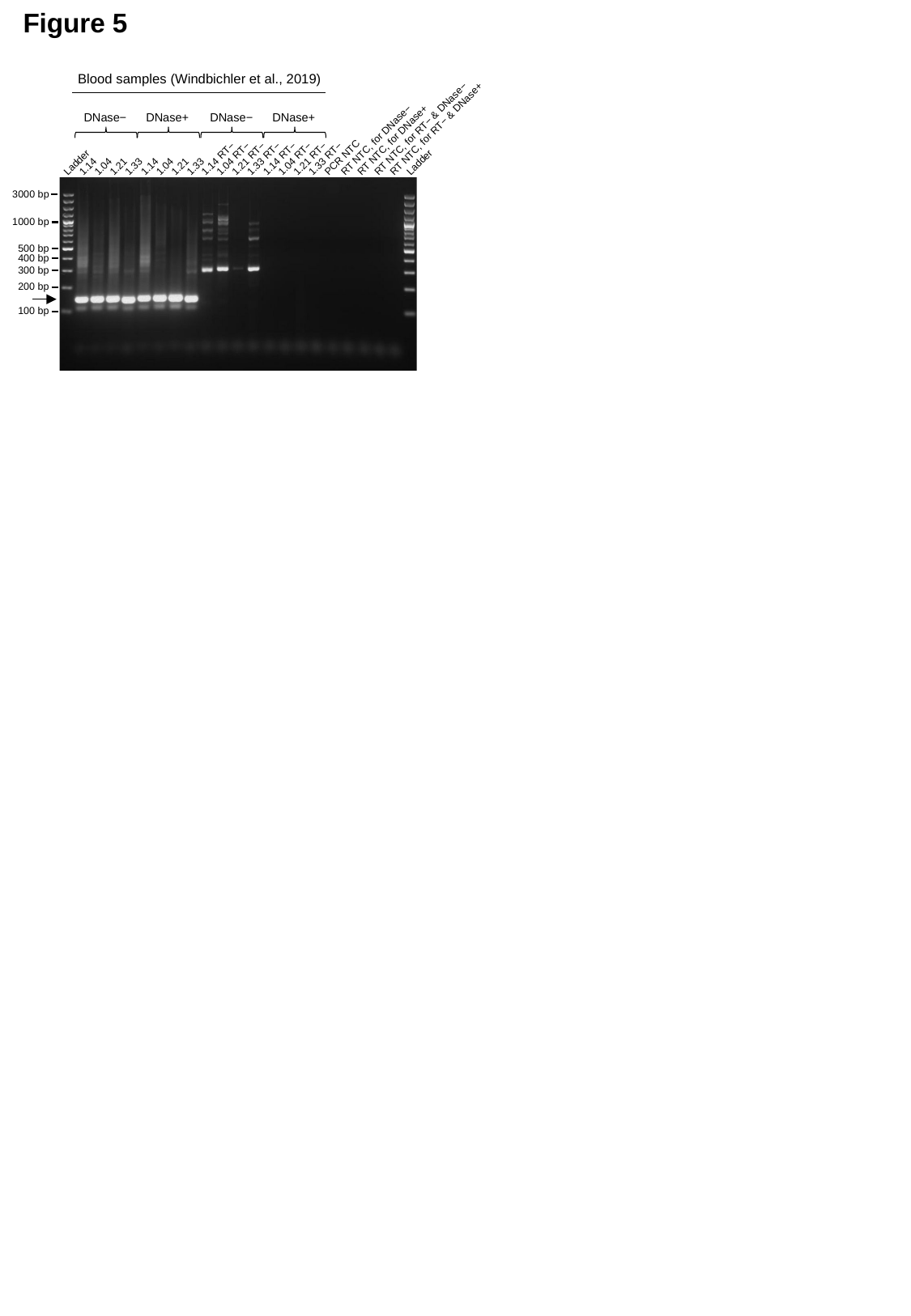

Figure 5
Blood samples (Windbichler et al., 2019)
DNase−
DNase+
DNase−
DNase+
RT NTC, for RT− & DNase−
RT NTC, for RT− & DNase+
RT NTC, for DNase−
RT NTC, for DNase+
PCR NTC
1.14 RT−
1.04 RT−
1.21 RT−
1.33 RT−
1.14 RT−
1.04 RT−
1.21 RT−
1.33 RT−
Ladder
Ladder
1.14
1.04
1.21
1.33
1.14
1.04
1.21
1.33
3000 bp
1000 bp
500 bp
400 bp
300 bp
200 bp
100 bp

## Slide 6
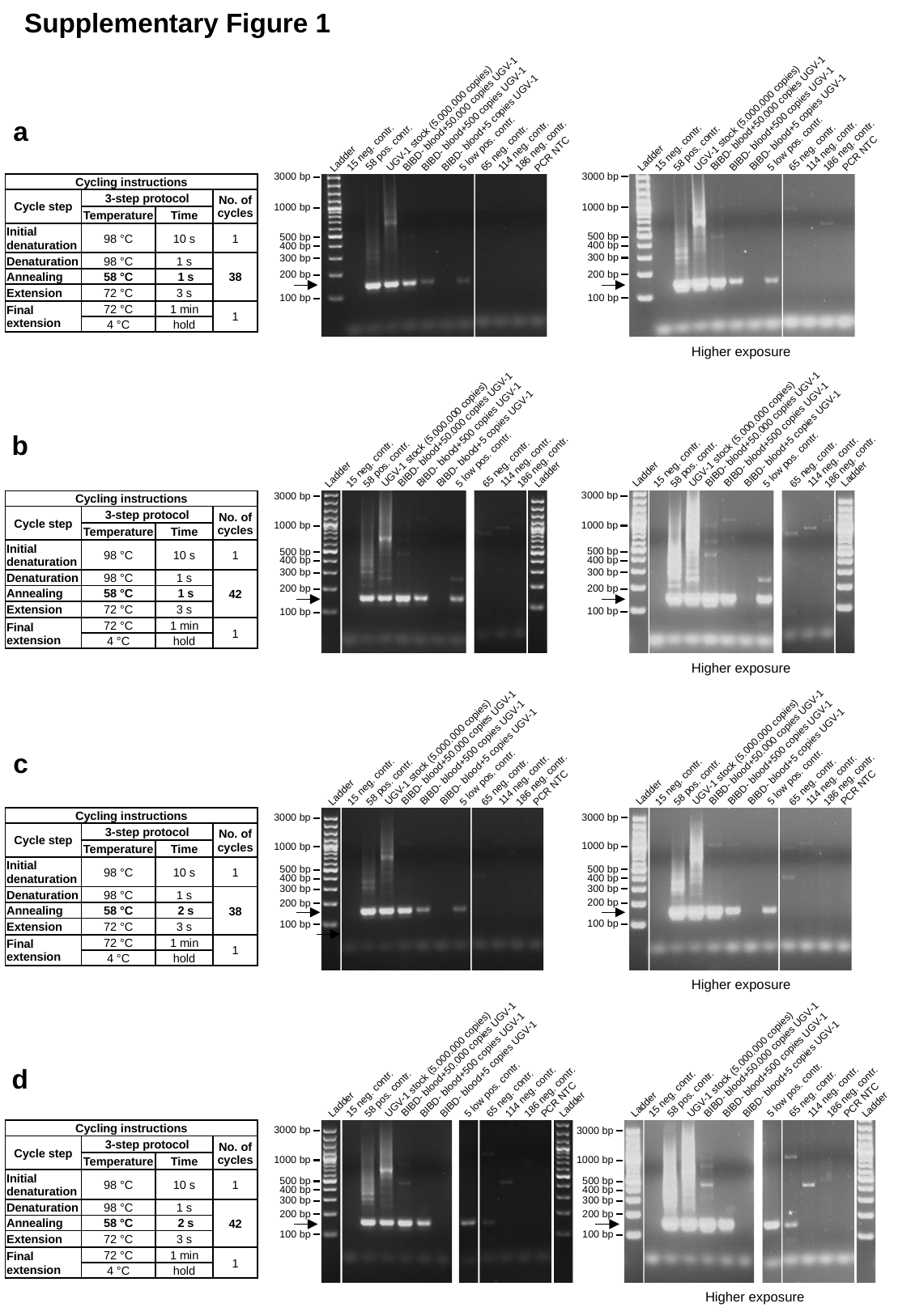

Supplementary Figure 1
BIBD- blood+50.000 copies UGV-1
BIBD- blood+50.000 copies UGV-1
BIBD- blood+500 copies UGV-1
BIBD- blood+500 copies UGV-1
UGV-1 stock (5.000.000 copies)
UGV-1 stock (5.000.000 copies)
a
BIBD- blood+5 copies UGV-1
BIBD- blood+5 copies UGV-1
5 low pos. contr.
5 low pos. contr.
114 neg. contr.
186 neg. contr.
114 neg. contr.
186 neg. contr.
58 pos. contr.
15 neg. contr.
65 neg. contr.
15 neg. contr.
65 neg. contr.
58 pos. contr.
PCR NTC
PCR NTC
Ladder
Ladder
3000 bp
3000 bp
| Cycling instructions | | | |
| --- | --- | --- | --- |
| Cycle step | 3-step protocol | | No. of cycles |
| | Temperature | Time | |
| Initial denaturation | 98 °C | 10 s | 1 |
| Denaturation | 98 °C | 1 s | 38 |
| Annealing | 58 °C | 1 s | |
| Extension | 72 °C | 3 s | |
| Final extension | 72 °C | 1 min | 1 |
| | 4 °C | hold | |
1000 bp
1000 bp
500 bp
500 bp
400 bp
400 bp
300 bp
300 bp
200 bp
200 bp
100 bp
100 bp
Higher exposure
BIBD- blood+50.000 copies UGV-1
BIBD- blood+50.000 copies UGV-1
BIBD- blood+500 copies UGV-1
BIBD- blood+500 copies UGV-1
UGV-1 stock (5.000.000 copies)
UGV-1 stock (5.000.000 copies)
b
BIBD- blood+5 copies UGV-1
BIBD- blood+5 copies UGV-1
5 low pos. contr.
5 low pos. contr.
114 neg. contr.
186 neg. contr.
114 neg. contr.
186 neg. contr.
15 neg. contr.
65 neg. contr.
15 neg. contr.
65 neg. contr.
58 pos. contr.
58 pos. contr.
Ladder
Ladder
Ladder
Ladder
3000 bp
3000 bp
| Cycling instructions | | | |
| --- | --- | --- | --- |
| Cycle step | 3-step protocol | | No. of cycles |
| | Temperature | Time | |
| Initial denaturation | 98 °C | 10 s | 1 |
| Denaturation | 98 °C | 1 s | 42 |
| Annealing | 58 °C | 1 s | |
| Extension | 72 °C | 3 s | |
| Final extension | 72 °C | 1 min | 1 |
| | 4 °C | hold | |
1000 bp
1000 bp
500 bp
500 bp
400 bp
400 bp
300 bp
300 bp
200 bp
200 bp
100 bp
100 bp
Higher exposure
BIBD- blood+50.000 copies UGV-1
BIBD- blood+50.000 copies UGV-1
BIBD- blood+500 copies UGV-1
BIBD- blood+500 copies UGV-1
UGV-1 stock (5.000.000 copies)
UGV-1 stock (5.000.000 copies)
c
BIBD- blood+5 copies UGV-1
BIBD- blood+5 copies UGV-1
5 low pos. contr.
5 low pos. contr.
114 neg. contr.
186 neg. contr.
114 neg. contr.
186 neg. contr.
15 neg. contr.
65 neg. contr.
15 neg. contr.
65 neg. contr.
58 pos. contr.
58 pos. contr.
PCR NTC
PCR NTC
Ladder
Ladder
3000 bp
3000 bp
| Cycling instructions | | | |
| --- | --- | --- | --- |
| Cycle step | 3-step protocol | | No. of cycles |
| | Temperature | Time | |
| Initial denaturation | 98 °C | 10 s | 1 |
| Denaturation | 98 °C | 1 s | 38 |
| Annealing | 58 °C | 2 s | |
| Extension | 72 °C | 3 s | |
| Final extension | 72 °C | 1 min | 1 |
| | 4 °C | hold | |
1000 bp
1000 bp
500 bp
500 bp
400 bp
400 bp
300 bp
300 bp
200 bp
200 bp
100 bp
100 bp
Higher exposure
BIBD- blood+50.000 copies UGV-1
BIBD- blood+50.000 copies UGV-1
BIBD- blood+500 copies UGV-1
BIBD- blood+500 copies UGV-1
UGV-1 stock (5.000.000 copies)
UGV-1 stock (5.000.000 copies)
BIBD- blood+5 copies UGV-1
BIBD- blood+5 copies UGV-1
d
5 low pos. contr.
5 low pos. contr.
114 neg. contr.
186 neg. contr.
114 neg. contr.
186 neg. contr.
15 neg. contr.
65 neg. contr.
15 neg. contr.
65 neg. contr.
58 pos. contr.
58 pos. contr.
PCR NTC
PCR NTC
Ladder
Ladder
Ladder
Ladder
3000 bp
3000 bp
| Cycling instructions | | | |
| --- | --- | --- | --- |
| Cycle step | 3-step protocol | | No. of cycles |
| | Temperature | Time | |
| Initial denaturation | 98 °C | 10 s | 1 |
| Denaturation | 98 °C | 1 s | 42 |
| Annealing | 58 °C | 2 s | |
| Extension | 72 °C | 3 s | |
| Final extension | 72 °C | 1 min | 1 |
| | 4 °C | hold | |
1000 bp
1000 bp
500 bp
500 bp
400 bp
400 bp
300 bp
300 bp
*
200 bp
200 bp
6
100 bp
100 bp
Higher exposure

## Slide 7
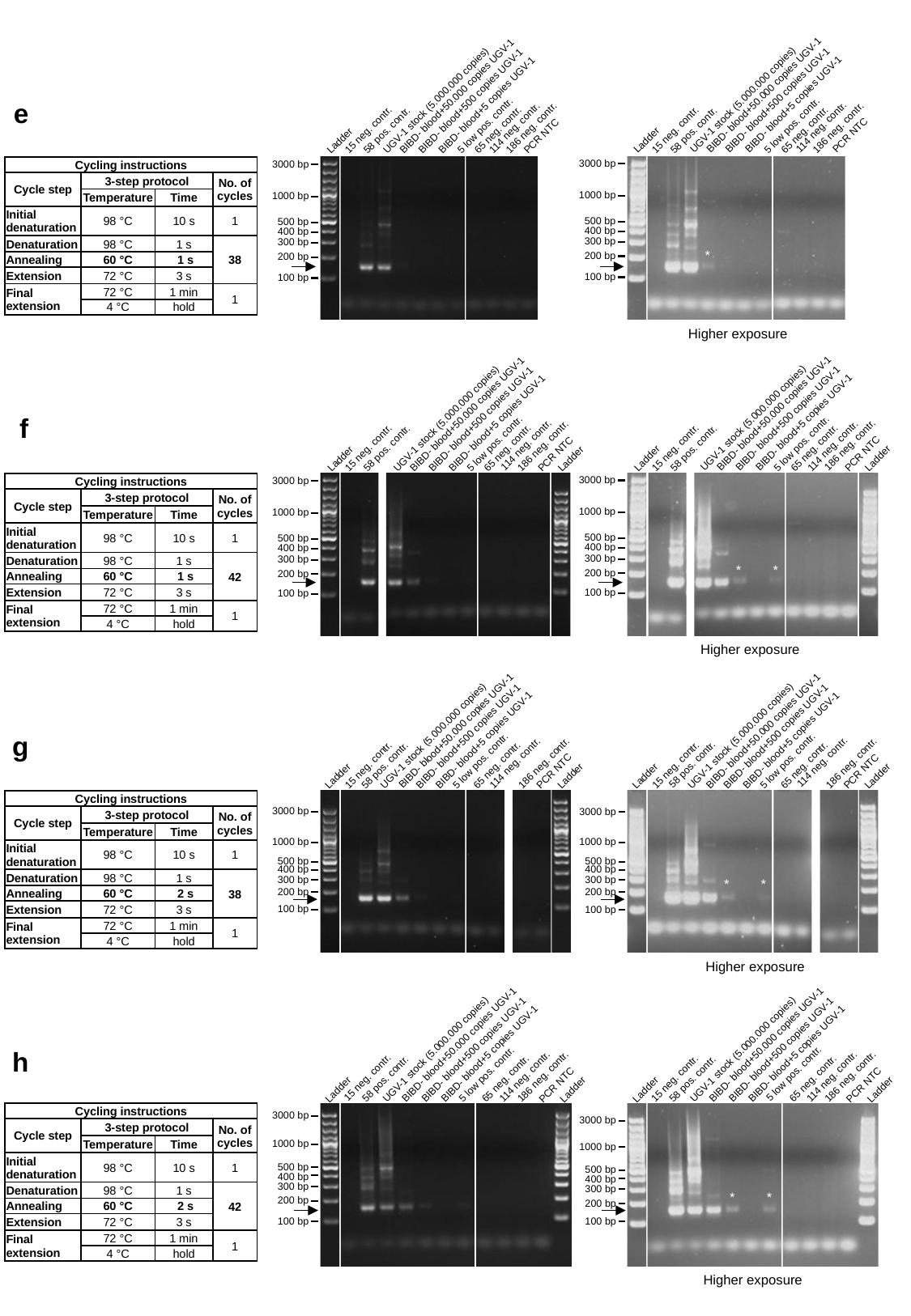

BIBD- blood+50.000 copies UGV-1
BIBD- blood+50.000 copies UGV-1
BIBD- blood+500 copies UGV-1
UGV-1 stock (5.000.000 copies)
BIBD- blood+500 copies UGV-1
UGV-1 stock (5.000.000 copies)
BIBD- blood+5 copies UGV-1
e
BIBD- blood+5 copies UGV-1
5 low pos. contr.
5 low pos. contr.
114 neg. contr.
114 neg. contr.
186 neg. contr.
186 neg. contr.
15 neg. contr.
65 neg. contr.
15 neg. contr.
65 neg. contr.
58 pos. contr.
58 pos. contr.
PCR NTC
PCR NTC
Ladder
Ladder
3000 bp
3000 bp
| Cycling instructions | | | |
| --- | --- | --- | --- |
| Cycle step | 3-step protocol | | No. of cycles |
| | Temperature | Time | |
| Initial denaturation | 98 °C | 10 s | 1 |
| Denaturation | 98 °C | 1 s | 38 |
| Annealing | 60 °C | 1 s | |
| Extension | 72 °C | 3 s | |
| Final extension | 72 °C | 1 min | 1 |
| | 4 °C | hold | |
1000 bp
1000 bp
500 bp
500 bp
400 bp
400 bp
300 bp
300 bp
*
200 bp
200 bp
100 bp
100 bp
Higher exposure
BIBD- blood+50.000 copies UGV-1
BIBD- blood+50.000 copies UGV-1
BIBD- blood+500 copies UGV-1
BIBD- blood+500 copies UGV-1
f
UGV-1 stock (5.000.000 copies)
UGV-1 stock (5.000.000 copies)
BIBD- blood+5 copies UGV-1
BIBD- blood+5 copies UGV-1
5 low pos. contr.
5 low pos. contr.
114 neg. contr.
186 neg. contr.
186 neg. contr.
114 neg. contr.
15 neg. contr.
65 neg. contr.
15 neg. contr.
65 neg. contr.
58 pos. contr.
58 pos. contr.
PCR NTC
PCR NTC
Ladder
Ladder
Ladder
Ladder
3000 bp
3000 bp
| Cycling instructions | | | |
| --- | --- | --- | --- |
| Cycle step | 3-step protocol | | No. of cycles |
| | Temperature | Time | |
| Initial denaturation | 98 °C | 10 s | 1 |
| Denaturation | 98 °C | 1 s | 42 |
| Annealing | 60 °C | 1 s | |
| Extension | 72 °C | 3 s | |
| Final extension | 72 °C | 1 min | 1 |
| | 4 °C | hold | |
1000 bp
1000 bp
500 bp
500 bp
400 bp
400 bp
300 bp
300 bp
*
*
200 bp
200 bp
100 bp
100 bp
Higher exposure
BIBD- blood+50.000 copies UGV-1
BIBD- blood+50.000 copies UGV-1
BIBD- blood+500 copies UGV-1
BIBD- blood+500 copies UGV-1
UGV-1 stock (5.000.000 copies)
UGV-1 stock (5.000.000 copies)
g
BIBD- blood+5 copies UGV-1
BIBD- blood+5 copies UGV-1
5 low pos. contr.
5 low pos. contr.
114 neg. contr.
114 neg. contr.
186 neg. contr.
186 neg. contr.
58 pos. contr.
15 neg. contr.
65 neg. contr.
15 neg. contr.
65 neg. contr.
58 pos. contr.
PCR NTC
PCR NTC
Ladder
Ladder
Ladder
Ladder
| Cycling instructions | | | |
| --- | --- | --- | --- |
| Cycle step | 3-step protocol | | No. of cycles |
| | Temperature | Time | |
| Initial denaturation | 98 °C | 10 s | 1 |
| Denaturation | 98 °C | 1 s | 38 |
| Annealing | 60 °C | 2 s | |
| Extension | 72 °C | 3 s | |
| Final extension | 72 °C | 1 min | 1 |
| | 4 °C | hold | |
3000 bp
3000 bp
1000 bp
1000 bp
500 bp
500 bp
400 bp
400 bp
300 bp
300 bp
*
*
200 bp
200 bp
100 bp
100 bp
Higher exposure
BIBD- blood+50.000 copies UGV-1
BIBD- blood+50.000 copies UGV-1
BIBD- blood+500 copies UGV-1
BIBD- blood+500 copies UGV-1
UGV-1 stock (5.000.000 copies)
UGV-1 stock (5.000.000 copies)
h
BIBD- blood+5 copies UGV-1
BIBD- blood+5 copies UGV-1
5 low pos. contr.
5 low pos. contr.
114 neg. contr.
186 neg. contr.
114 neg. contr.
186 neg. contr.
15 neg. contr.
65 neg. contr.
15 neg. contr.
65 neg. contr.
58 pos. contr.
58 pos. contr.
PCR NTC
PCR NTC
Ladder
Ladder
Ladder
Ladder
3000 bp
| Cycling instructions | | | |
| --- | --- | --- | --- |
| Cycle step | 3-step protocol | | No. of cycles |
| | Temperature | Time | |
| Initial denaturation | 98 °C | 10 s | 1 |
| Denaturation | 98 °C | 1 s | 42 |
| Annealing | 60 °C | 2 s | |
| Extension | 72 °C | 3 s | |
| Final extension | 72 °C | 1 min | 1 |
| | 4 °C | hold | |
3000 bp
1000 bp
1000 bp
500 bp
500 bp
400 bp
400 bp
300 bp
300 bp
*
*
200 bp
200 bp
100 bp
100 bp
7
Higher exposure

## Slide 8
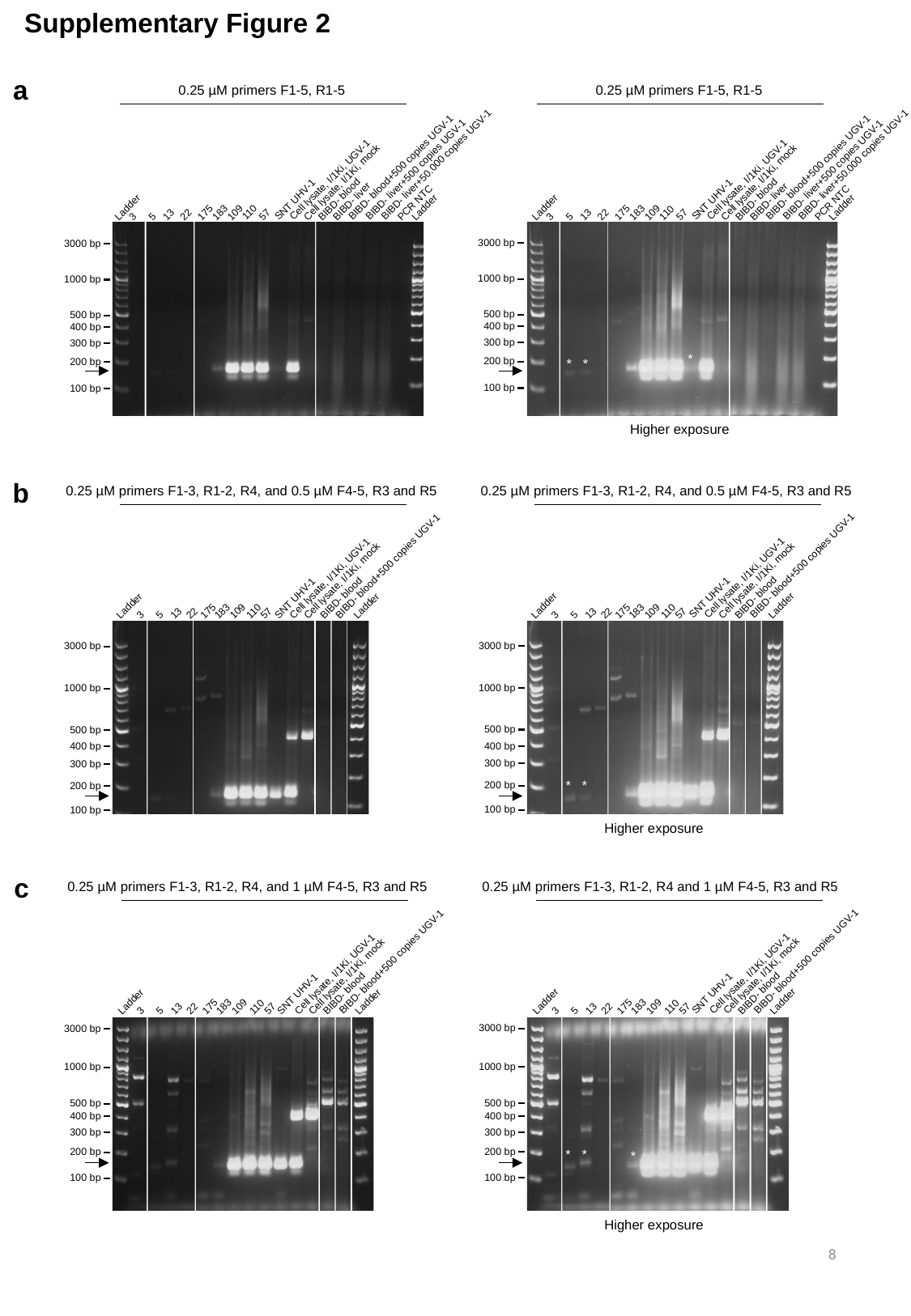

Supplementary Figure 2
a
0.25 µM primers F1-5, R1-5
0.25 µM primers F1-5, R1-5
BIBD- liver+50.000 copies UGV-1
BIBD- liver+50.000 copies UGV-1
BIBD- blood+500 copies UGV-1
BIBD- blood+500 copies UGV-1
BIBD- liver+500 copies UGV-1
BIBD- liver+500 copies UGV-1
Cell lysate, I/1Ki, UGV-1
Cell lysate, I/1Ki, UGV-1
Cell lysate, I/1Ki, mock
Cell lysate, I/1Ki, mock
BIBD- blood
BIBD- blood
SNT UHV-1
SNT UHV-1
BIBD- liver
BIBD- liver
PCR NTC
PCR NTC
Ladder
Ladder
Ladder
Ladder
175
183
109
110
175
183
109
110
13
22
57
13
22
57
3
5
3
5
3000 bp
3000 bp
1000 bp
1000 bp
500 bp
500 bp
400 bp
400 bp
300 bp
300 bp
*
200 bp
200 bp
*
*
100 bp
100 bp
Higher exposure
b
0.25 µM primers F1-3, R1-2, R4, and 0.5 µM F4-5, R3 and R5
0.25 µM primers F1-3, R1-2, R4, and 0.5 µM F4-5, R3 and R5
BIBD- blood+500 copies UGV-1
BIBD- blood+500 copies UGV-1
Cell lysate, I/1Ki, UGV-1
Cell lysate, I/1Ki, UGV-1
Cell lysate, I/1Ki, mock
Cell lysate, I/1Ki, mock
BIBD- blood
SNT UHV-1
BIBD- blood
SNT UHV-1
Ladder
Ladder
Ladder
Ladder
175
183
109
110
175
183
109
110
13
22
57
13
22
57
3
5
3
5
3000 bp
3000 bp
1000 bp
1000 bp
500 bp
500 bp
400 bp
400 bp
300 bp
300 bp
*
*
200 bp
200 bp
100 bp
100 bp
Higher exposure
c
0.25 µM primers F1-3, R1-2, R4 and 1 µM F4-5, R3 and R5
0.25 µM primers F1-3, R1-2, R4, and 1 µM F4-5, R3 and R5
BIBD- blood+500 copies UGV-1
BIBD- blood+500 copies UGV-1
Cell lysate, I/1Ki, UGV-1
Cell lysate, I/1Ki, UGV-1
Cell lysate, I/1Ki, mock
Cell lysate, I/1Ki, mock
BIBD- blood
SNT UHV-1
BIBD- blood
SNT UHV-1
Ladder
Ladder
Ladder
Ladder
175
183
109
110
175
183
109
110
13
22
57
13
22
57
3
5
3
5
3000 bp
3000 bp
1000 bp
1000 bp
500 bp
500 bp
400 bp
400 bp
300 bp
300 bp
200 bp
200 bp
*
*
*
100 bp
100 bp
Higher exposure
8

## Slide 9
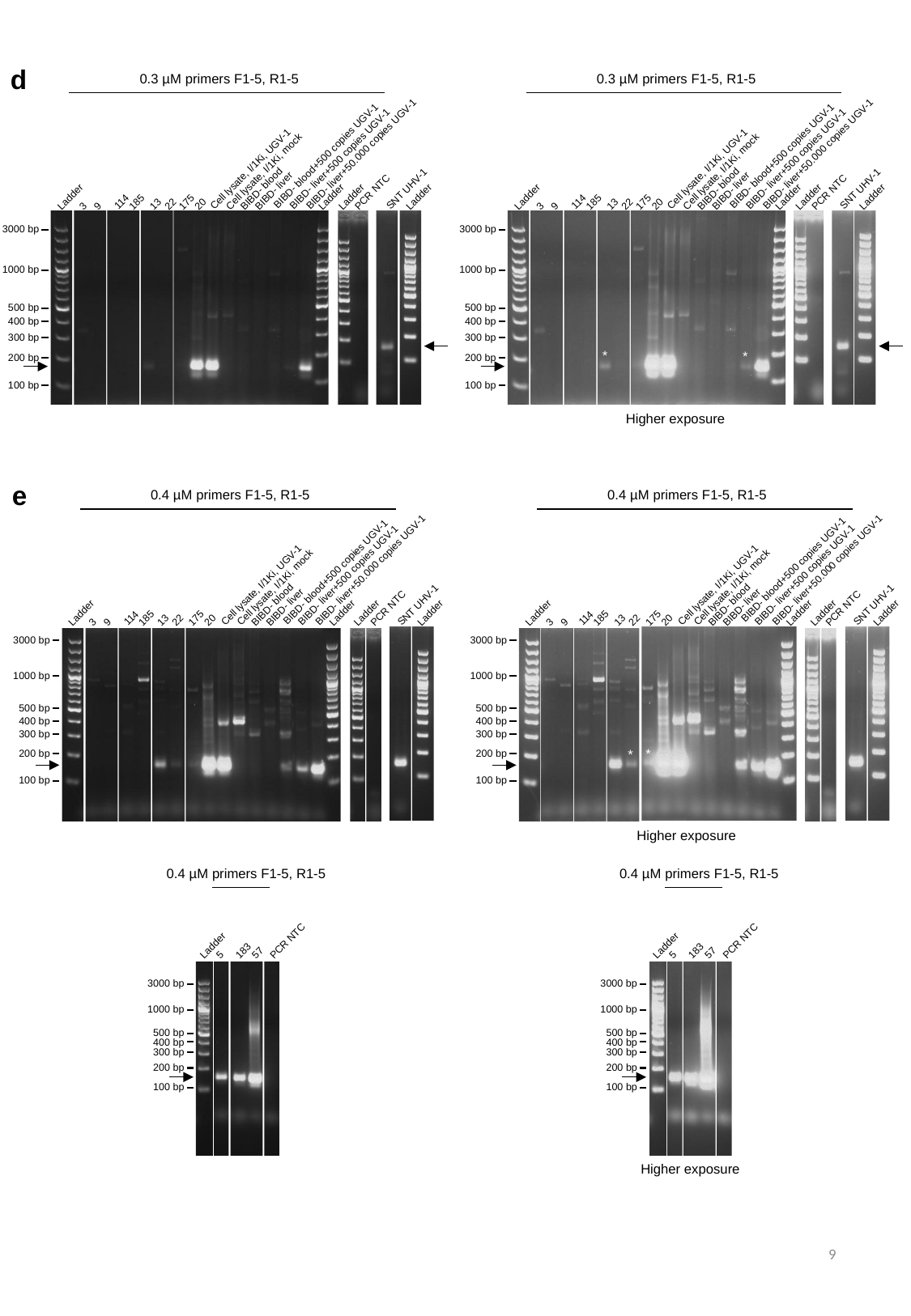

d
0.3 µM primers F1-5, R1-5
0.3 µM primers F1-5, R1-5
BIBD- liver+50.000 copies UGV-1
BIBD- liver+50.000 copies UGV-1
BIBD- blood+500 copies UGV-1
BIBD- blood+500 copies UGV-1
BIBD- liver+500 copies UGV-1
BIBD- liver+500 copies UGV-1
Cell lysate, I/1Ki, UGV-1
Cell lysate, I/1Ki, UGV-1
Cell lysate, I/1Ki, mock
Cell lysate, I/1Ki, mock
BIBD- blood
BIBD- blood
SNT UHV-1
SNT UHV-1
BIBD- liver
BIBD- liver
PCR NTC
PCR NTC
Ladder
Ladder
Ladder
Ladder
Ladder
Ladder
Ladder
Ladder
114
185
175
114
185
175
13
22
20
13
22
20
3
9
3
9
3000 bp
3000 bp
1000 bp
1000 bp
500 bp
500 bp
400 bp
400 bp
300 bp
300 bp
*
*
200 bp
200 bp
100 bp
100 bp
Higher exposure
e
0.4 µM primers F1-5, R1-5
0.4 µM primers F1-5, R1-5
BIBD- blood+500 copies UGV-1
BIBD- liver+50.000 copies UGV-1
BIBD- liver+50.000 copies UGV-1
BIBD- blood+500 copies UGV-1
BIBD- liver+500 copies UGV-1
BIBD- liver+500 copies UGV-1
Cell lysate, I/1Ki, UGV-1
Cell lysate, I/1Ki, UGV-1
Cell lysate, I/1Ki, mock
Cell lysate, I/1Ki, mock
BIBD- blood
BIBD- blood
SNT UHV-1
SNT UHV-1
BIBD- liver
BIBD- liver
PCR NTC
PCR NTC
Ladder
Ladder
Ladder
Ladder
Ladder
Ladder
Ladder
Ladder
114
185
175
114
185
175
13
22
20
13
22
20
3
9
3
9
3000 bp
3000 bp
1000 bp
1000 bp
500 bp
500 bp
400 bp
400 bp
300 bp
300 bp
*
*
200 bp
200 bp
100 bp
100 bp
Higher exposure
0.4 µM primers F1-5, R1-5
0.4 µM primers F1-5, R1-5
PCR NTC
PCR NTC
Ladder
Ladder
183
183
57
57
5
5
3000 bp
3000 bp
1000 bp
1000 bp
500 bp
500 bp
400 bp
400 bp
300 bp
300 bp
200 bp
200 bp
100 bp
100 bp
Higher exposure
9

## Slide 10
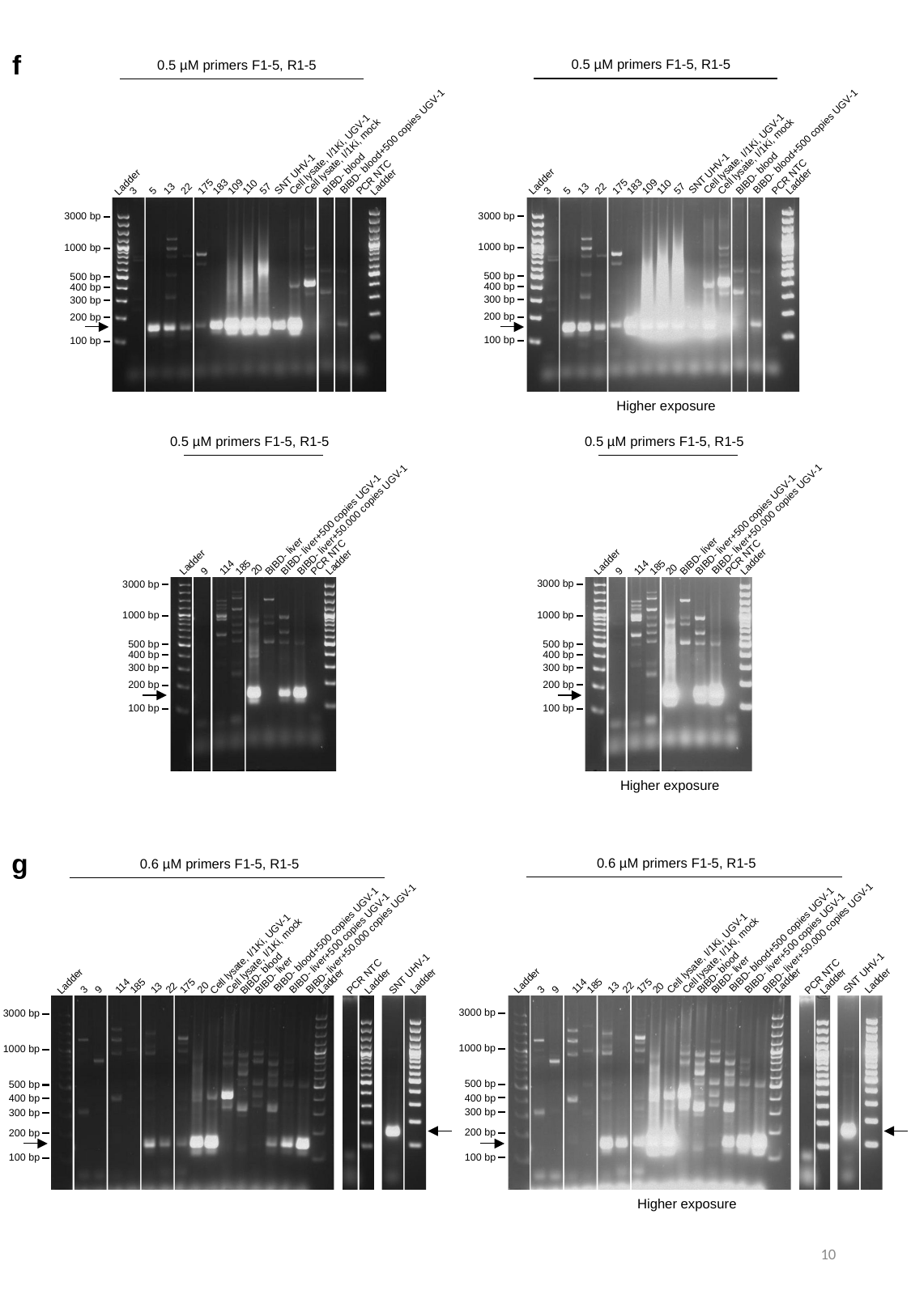

f
0.5 µM primers F1-5, R1-5
0.5 µM primers F1-5, R1-5
BIBD- blood+500 copies UGV-1
BIBD- blood+500 copies UGV-1
Cell lysate, I/1Ki, UGV-1
Cell lysate, I/1Ki, UGV-1
Cell lysate, I/1Ki, mock
Cell lysate, I/1Ki, mock
BIBD- blood
SNT UHV-1
BIBD- blood
SNT UHV-1
PCR NTC
PCR NTC
Ladder
Ladder
Ladder
Ladder
175
183
109
110
175
183
109
110
13
22
57
13
22
57
3
5
3
5
3000 bp
3000 bp
1000 bp
1000 bp
500 bp
500 bp
400 bp
400 bp
300 bp
300 bp
200 bp
200 bp
100 bp
100 bp
Higher exposure
0.5 µM primers F1-5, R1-5
0.5 µM primers F1-5, R1-5
BIBD- liver+50.000 copies UGV-1
BIBD- liver+50.000 copies UGV-1
BIBD- liver+500 copies UGV-1
BIBD- liver+500 copies UGV-1
BIBD- liver
BIBD- liver
PCR NTC
PCR NTC
Ladder
Ladder
Ladder
Ladder
114
185
114
185
20
20
9
9
3000 bp
3000 bp
1000 bp
1000 bp
500 bp
500 bp
400 bp
400 bp
300 bp
300 bp
200 bp
200 bp
100 bp
100 bp
Higher exposure
g
0.6 µM primers F1-5, R1-5
0.6 µM primers F1-5, R1-5
BIBD- liver+50.000 copies UGV-1
BIBD- liver+50.000 copies UGV-1
BIBD- blood+500 copies UGV-1
BIBD- blood+500 copies UGV-1
BIBD- liver+500 copies UGV-1
BIBD- liver+500 copies UGV-1
Cell lysate, I/1Ki, UGV-1
Cell lysate, I/1Ki, UGV-1
Cell lysate, I/1Ki, mock
Cell lysate, I/1Ki, mock
BIBD- blood
SNT UHV-1
BIBD- blood
SNT UHV-1
BIBD- liver
BIBD- liver
PCR NTC
PCR NTC
Ladder
Ladder
Ladder
Ladder
Ladder
Ladder
Ladder
Ladder
114
185
175
114
185
175
13
22
20
13
22
20
3
9
3
9
3000 bp
3000 bp
1000 bp
1000 bp
500 bp
500 bp
400 bp
400 bp
300 bp
300 bp
200 bp
200 bp
100 bp
100 bp
Higher exposure
10

## Slide 11
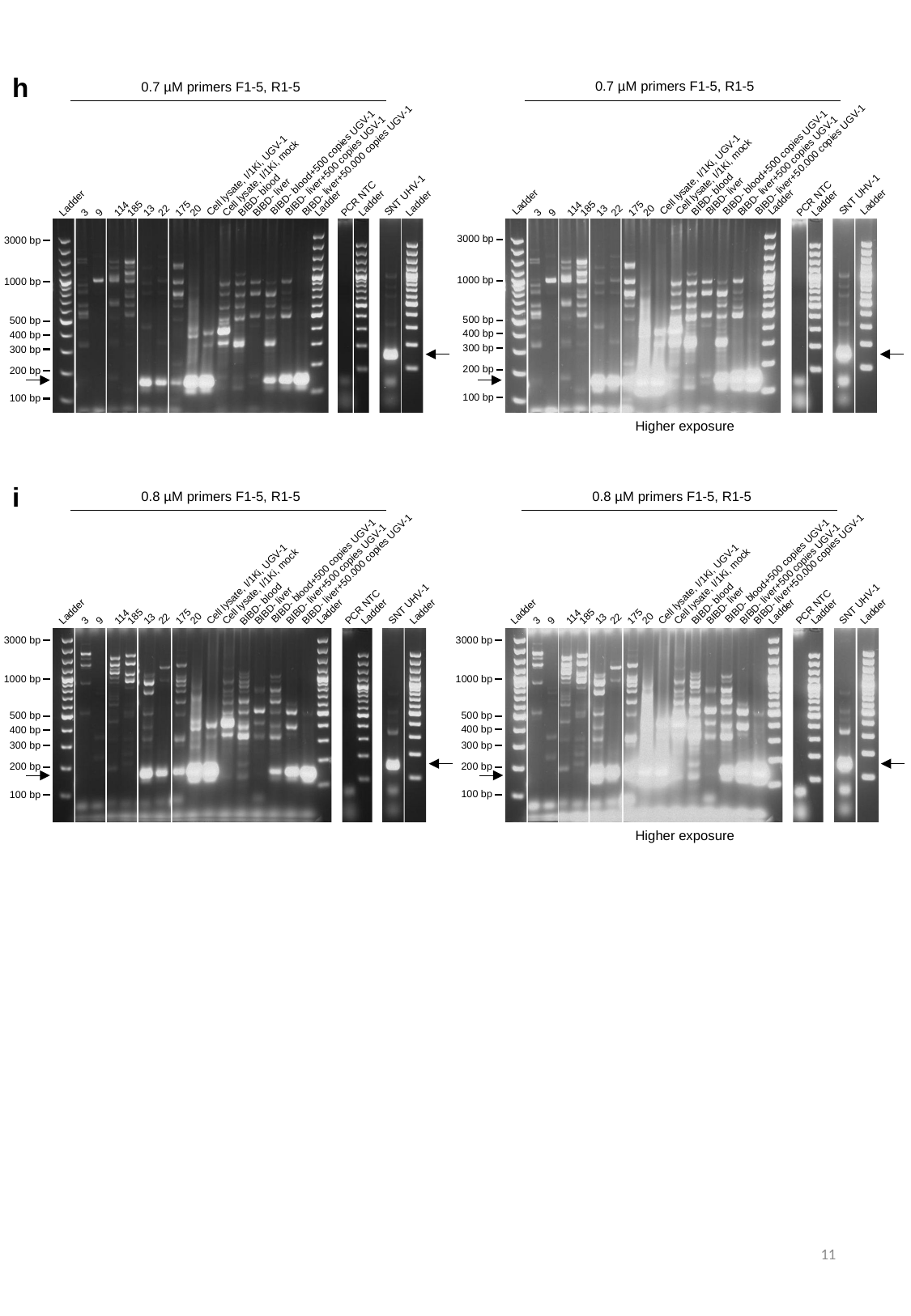

h
0.7 µM primers F1-5, R1-5
0.7 µM primers F1-5, R1-5
BIBD- liver+50.000 copies UGV-1
BIBD- liver+50.000 copies UGV-1
BIBD- blood+500 copies UGV-1
BIBD- blood+500 copies UGV-1
BIBD- liver+500 copies UGV-1
BIBD- liver+500 copies UGV-1
Cell lysate, I/1Ki, UGV-1
Cell lysate, I/1Ki, UGV-1
Cell lysate, I/1Ki, mock
Cell lysate, I/1Ki, mock
BIBD- blood
SNT UHV-1
BIBD- blood
SNT UHV-1
BIBD- liver
BIBD- liver
PCR NTC
PCR NTC
Ladder
Ladder
Ladder
Ladder
Ladder
Ladder
Ladder
Ladder
114
185
175
114
185
175
13
22
20
13
22
20
3
9
3
9
3000 bp
3000 bp
1000 bp
1000 bp
500 bp
500 bp
400 bp
400 bp
300 bp
300 bp
200 bp
200 bp
100 bp
100 bp
Higher exposure
i
0.8 µM primers F1-5, R1-5
0.8 µM primers F1-5, R1-5
BIBD- liver+50.000 copies UGV-1
BIBD- liver+50.000 copies UGV-1
BIBD- blood+500 copies UGV-1
BIBD- blood+500 copies UGV-1
BIBD- liver+500 copies UGV-1
BIBD- liver+500 copies UGV-1
Cell lysate, I/1Ki, UGV-1
Cell lysate, I/1Ki, UGV-1
Cell lysate, I/1Ki, mock
Cell lysate, I/1Ki, mock
BIBD- blood
BIBD- blood
SNT UHV-1
SNT UHV-1
BIBD- liver
BIBD- liver
PCR NTC
PCR NTC
Ladder
Ladder
Ladder
Ladder
Ladder
Ladder
Ladder
Ladder
114
185
175
114
185
175
13
22
20
13
22
20
3
9
3
9
3000 bp
3000 bp
1000 bp
1000 bp
500 bp
500 bp
400 bp
400 bp
300 bp
300 bp
200 bp
200 bp
100 bp
100 bp
Higher exposure
11

## Slide 12
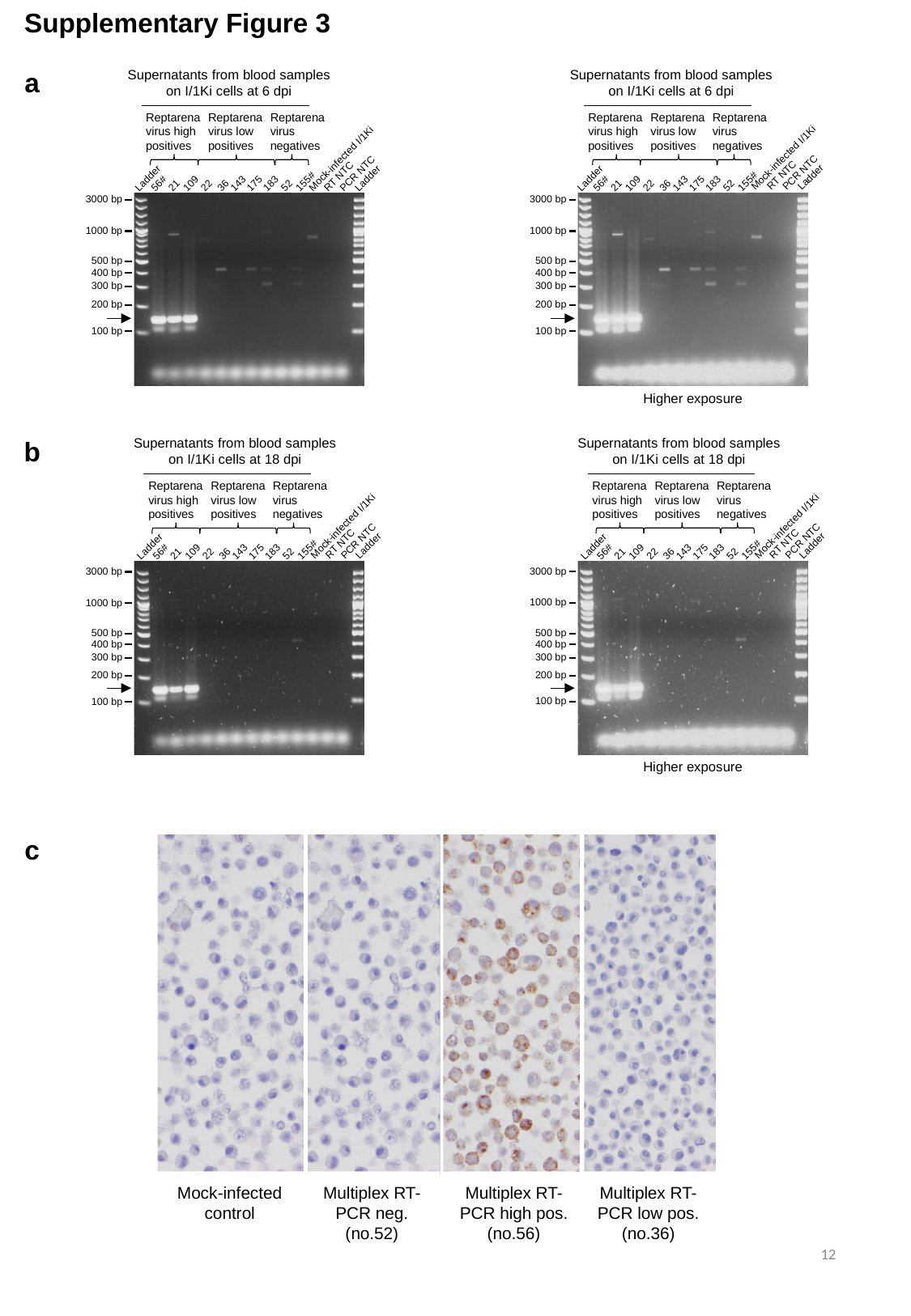

Supplementary Figure 3
a
Supernatants from blood samples on I/1Ki cells at 6 dpi
Supernatants from blood samples on I/1Ki cells at 6 dpi
Reptarenavirus high positives
Reptarenavirus low positives
Reptarenavirus negatives
Reptarenavirus high positives
Reptarenavirus low positives
Reptarenavirus negatives
Mock-infected I/1Ki
Mock-infected I/1Ki
PCR NTC
PCR NTC
RT NTC
RT NTC
Ladder
Ladder
Ladder
Ladder
155#
155#
56#
109
143
175
183
56#
109
143
175
183
21
22
36
52
21
22
36
52
3000 bp
3000 bp
1000 bp
1000 bp
500 bp
500 bp
400 bp
400 bp
300 bp
300 bp
200 bp
200 bp
100 bp
100 bp
Higher exposure
Supernatants from blood samples on I/1Ki cells at 18 dpi
b
Supernatants from blood samples on I/1Ki cells at 18 dpi
Reptarenavirus high positives
Reptarenavirus low positives
Reptarenavirus negatives
Reptarenavirus high positives
Reptarenavirus low positives
Reptarenavirus negatives
Mock-infected I/1Ki
Mock-infected I/1Ki
PCR NTC
PCR NTC
RT NTC
RT NTC
Ladder
Ladder
Ladder
Ladder
155#
155#
56#
56#
109
143
175
183
109
143
175
183
21
22
36
52
21
22
36
52
3000 bp
3000 bp
1000 bp
1000 bp
500 bp
500 bp
400 bp
400 bp
300 bp
300 bp
200 bp
200 bp
100 bp
100 bp
Higher exposure
c
Mock-infected control
Multiplex RT-PCR neg. (no.52)
Multiplex RT-PCR high pos. (no.56)
Multiplex RT-PCR low pos. (no.36)
12
