# Supplementary material for: A Multiplex RT-PCR Method for the Detection of Reptarenavirus Infection
Source: Viruses. 2023 Nov 25;15(12):2313. doi: 10.3390/v15122313 (PMC10747477; doi:10.3390/v15122313)
Supplement: Supplementary file 1 [file viruses-15-02313-s001.zip › Supplementary Material/Supplementary Figures.docx]

**Supplementary Figures**


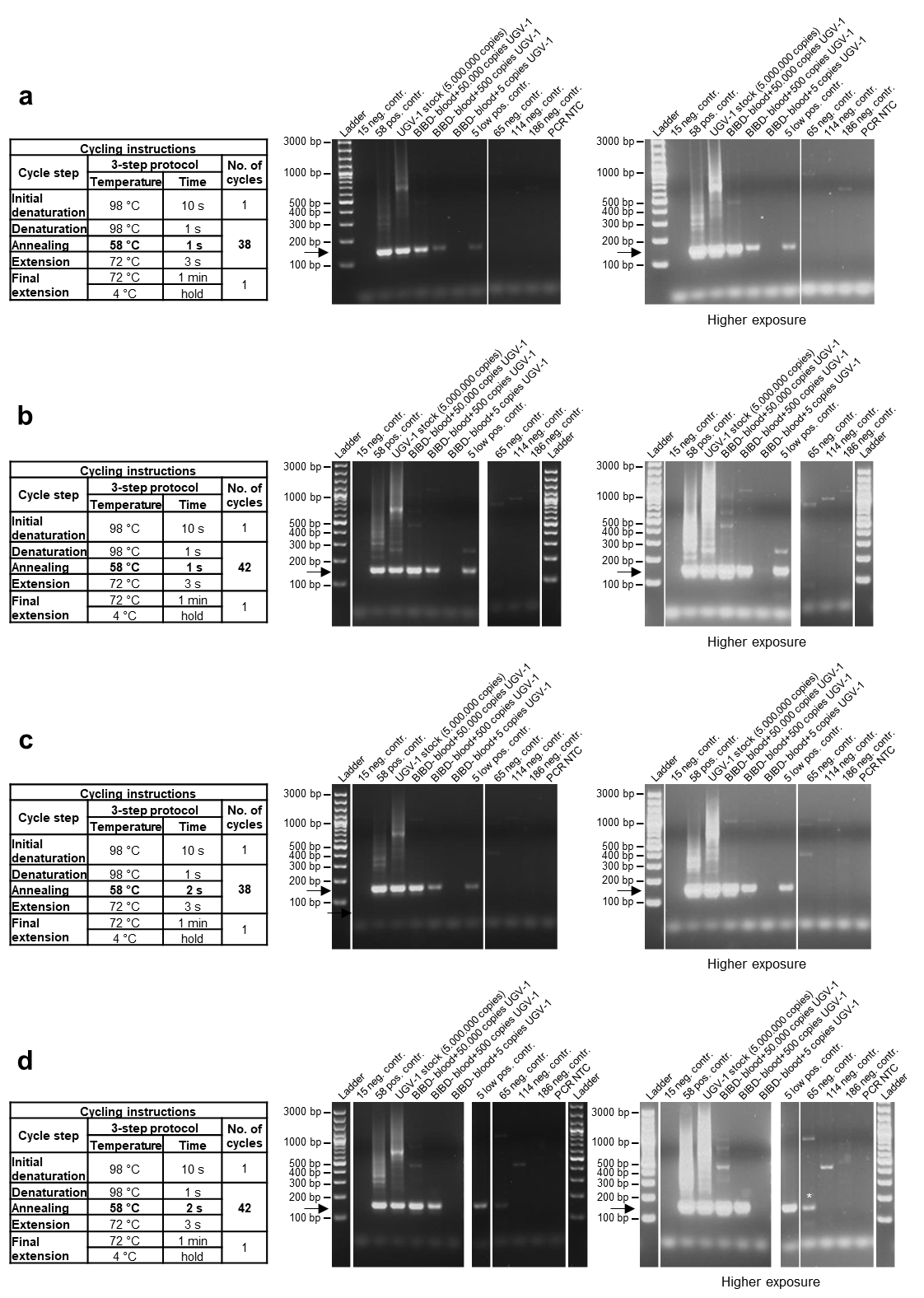
**Figure S1.** **Testing the multiplex RT-PCR protocol with different cycling conditions.**


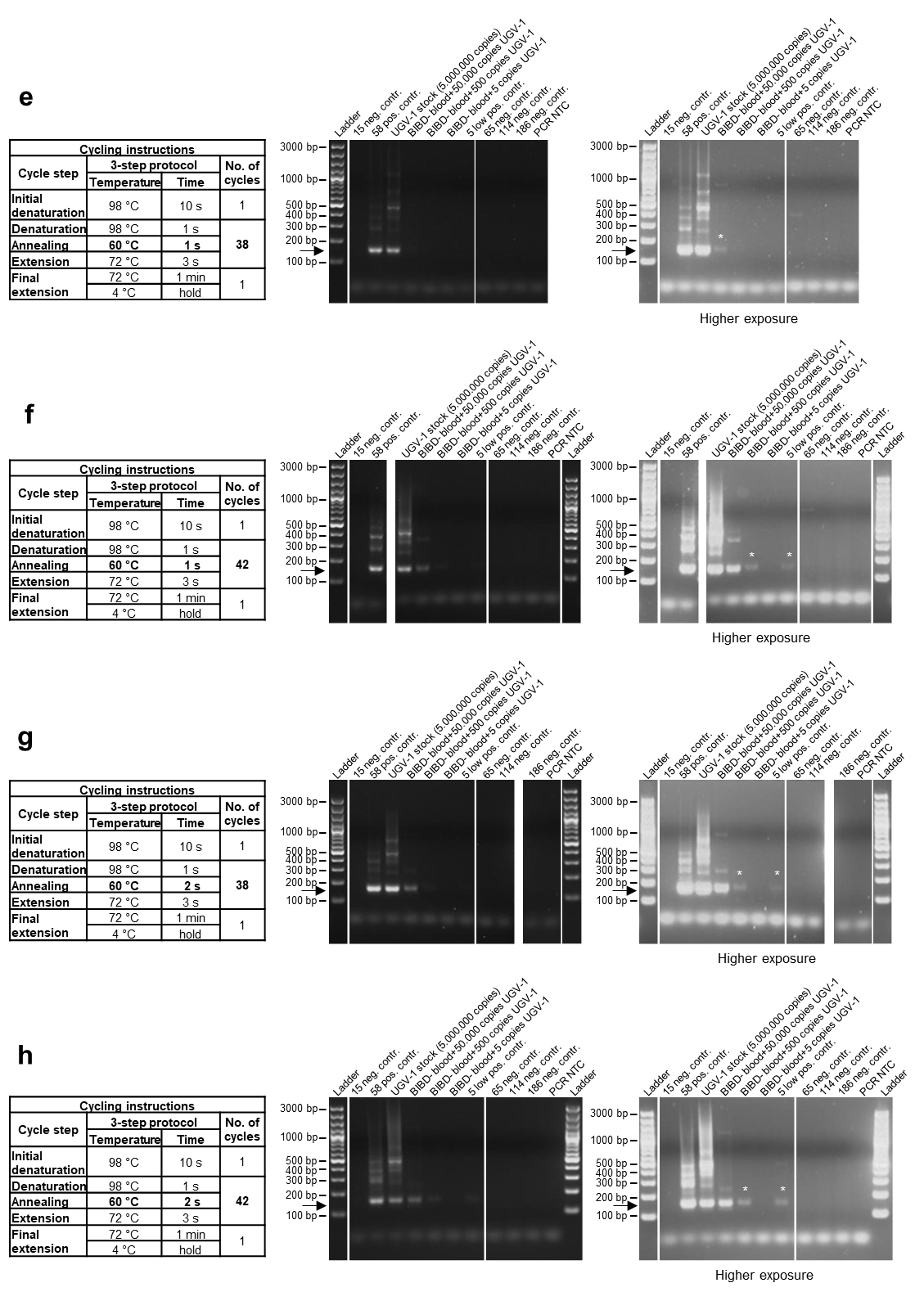


**Legend:** Different cycling conditions of the multiplex RT-PCR protocol were evaluated on cDNAs from: *i*) blood samples of formerly confirmed reptarenavirus-negative (neg.) or positive (pos.) snakes described in Thiele et al., 2023 [9] and used as controls (contr.); *ii*) UGV-1 viral stock (with 5 × 10^6^ S segment copies/lane); *iii*) a BIBD-negative (BIBD−) blood sample mixed with known copy numbers of UGV-1 S segments (5, 500 or 50.000 copies/lane). The following cycling conditions were applied: initial denaturation at 98 °C for 10 s and cycling denaturation at 98 °C for 1 s, annealing temperature of 58 °C (**a-d**) or 60 °C (**e-h**), annealing time of 1 s (**a**,**b** and **e**,**f**) or 2 s (**c**,**d** and **g**,**h**), extension time at 72 °C for 3 s, 38 (**a**,**c** and **e**,**g**) or 42 (**b**,**d** and **f**,**h**) no. of cycles of the denaturation/annealing/extension steps, and final extension at 72 °C for 1 min. The tested primers were F1-5 and R1-5, each one of them at the final concentration of 0.25 µM. The multiplex RT-PCR products were separated by agarose gel electrophoresis and the bands visualized under UV light by GelRed nucleic acid staining (Biotium, Fremont, CA, USA) pre-cast to the gels. Specific amplicons are at approximately 140 bp and are indicated by arrows. In each panel the images on the right represent the gels imaged at higher exposure times than on the left. Bands that became visible after higher exposure times are indicated by an *. 60 °C annealing temperature was excluded, as the sensitivity was generally much weaker than at 58 °C, with barely detectable specific bands of reptarenavirus low positive samples (**a**-**h**). At 58 °C annealing temperature, a no. of 42 cycles was excluded, as the output resulted in a generally more intense non-specific background compared to no. of 38 cycles, without significant improvement on the specificity (**a**-**d**). The annealing time of 2 s was preferred to the one of 1 s because it allowed to obtain stronger intensities of specific bands without affecting the background intensity (**a**,**c**). The extension time of 3 s for sequences of approximately 140 bp was in agreement with the manufacturer’s instructions of the Phusion Flash High-Fidelity PCR Master Mix (Thermo Fisher Scientific, Waltham, MA, USA), reporting an extension time of 15 s per 1 kb. Short spaces between gel lanes represent separate regions in the same gels, whereas longer spaces between gel lanes represent areas from different gels. Ladder: GeneRuler 100 bp plus DNA ladder (Thermo Fisher Scientific, Waltham, MA, USA); RT: reverse transcription; NTC: no template control.

**Figure S2.** **Testing the multiplex RT-PCR protocol with different primer concentrations.**

**
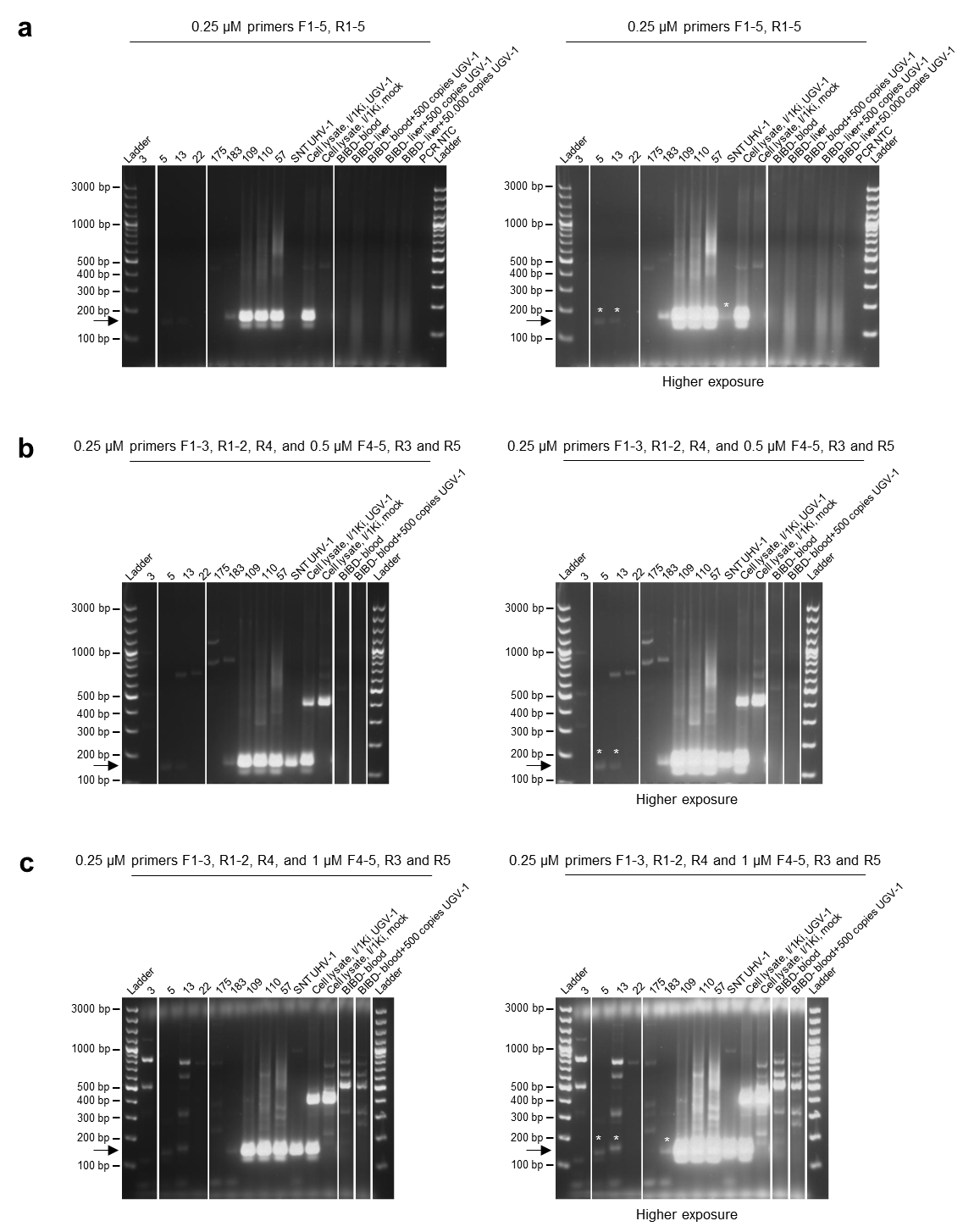
**

**
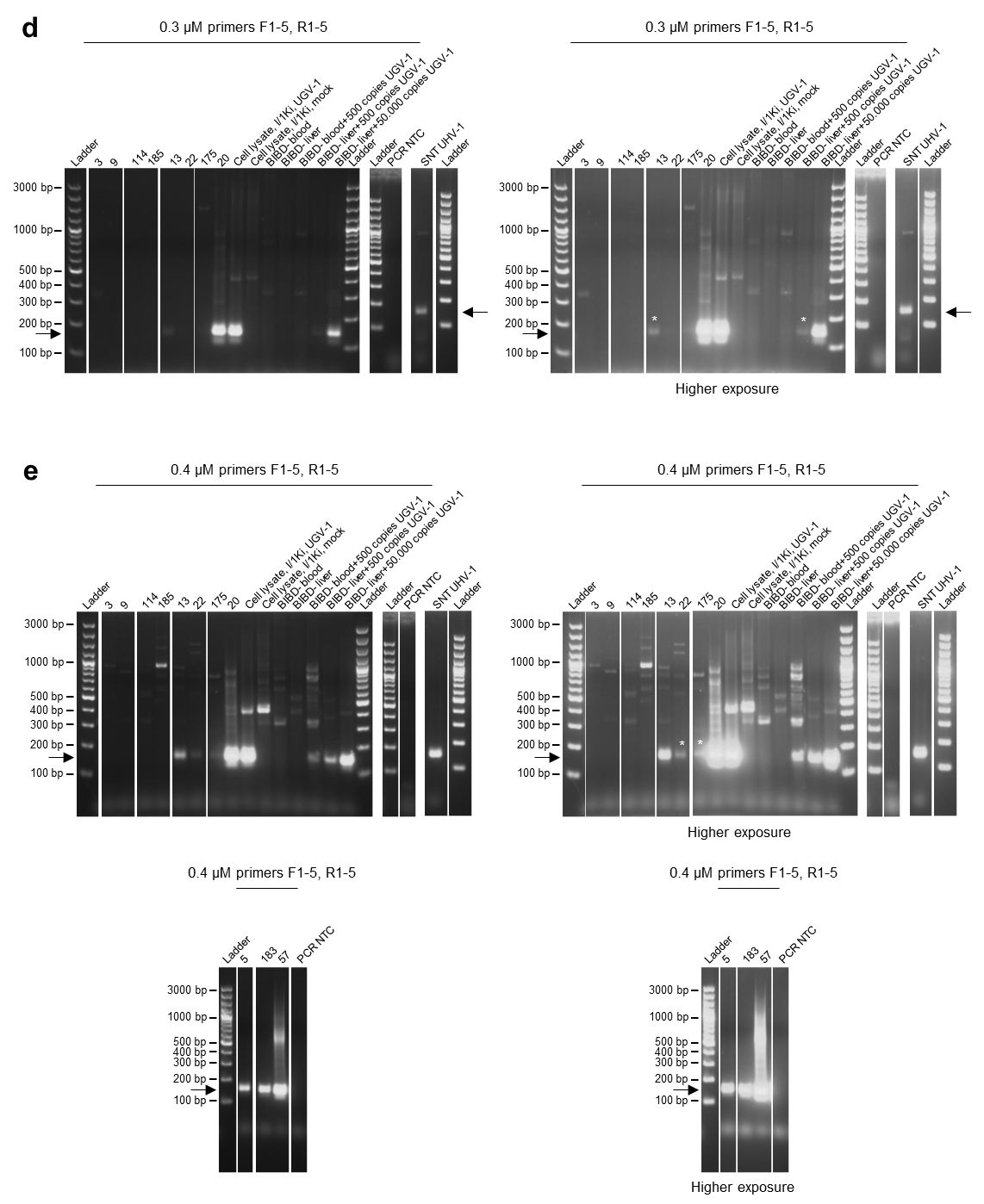
**

**
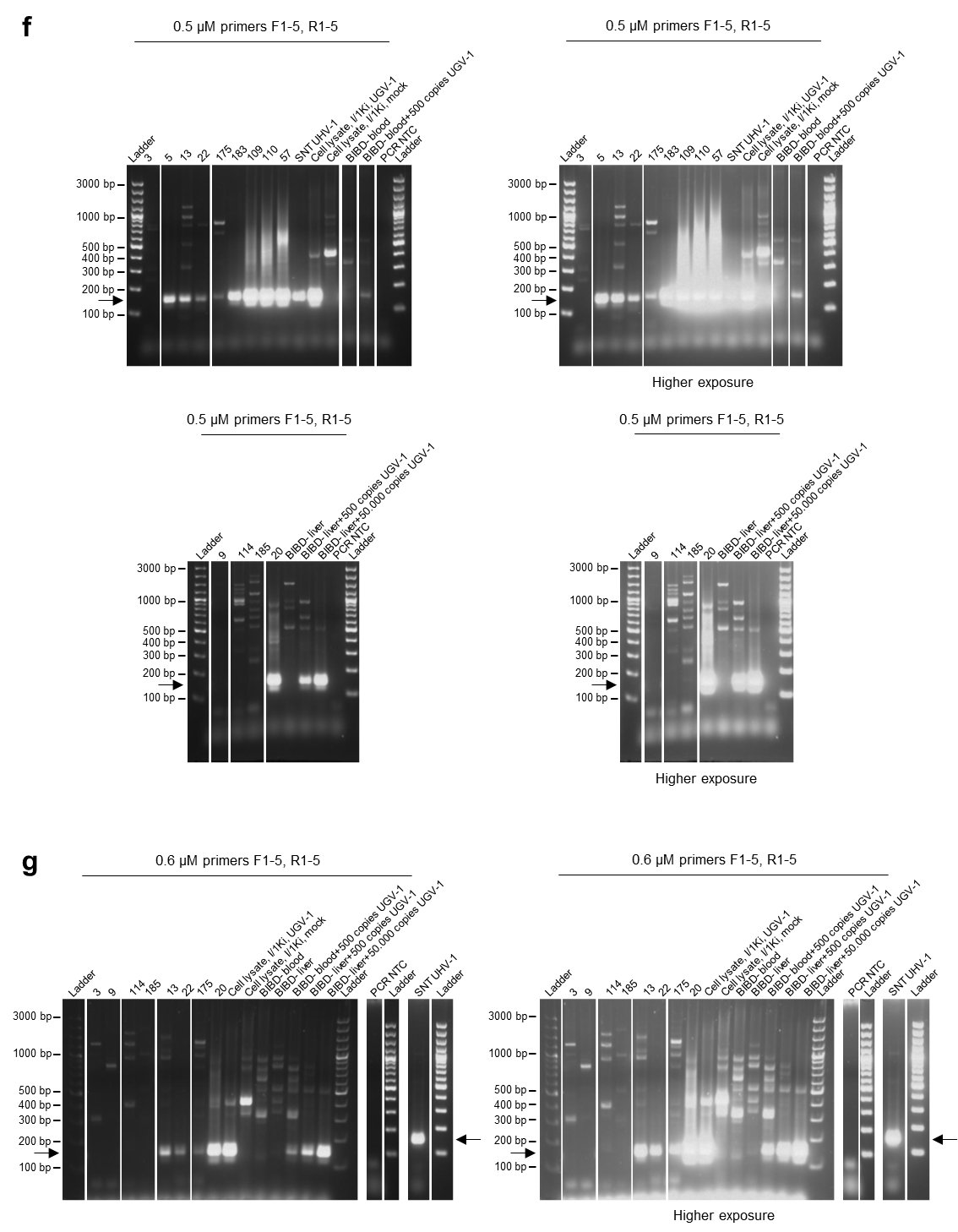
**

**
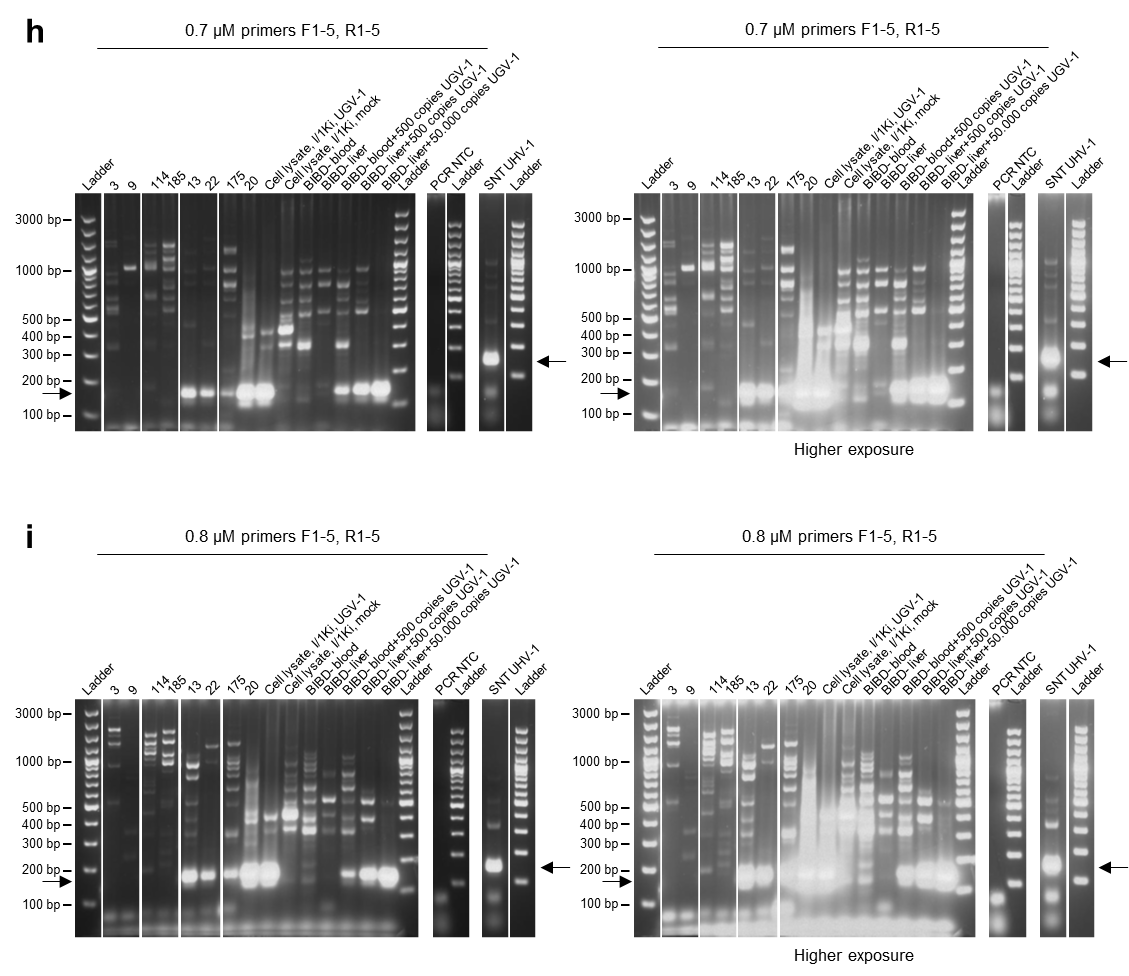
**

**Legend:** The multiplex RT-PCR protocol with reactions at different primer concentrations was tried out on: *i*) previously described diagnostic snake bloods samples [9]; *ii*) reptarenavirus-infected cell culture supernatant (SNT UHV-1); *iii*) UGV-1-infected or mock-infected cell culture lysates; *iv*) BIBD-negative (BIBD−) snake tissues (blood and liver) lysates, also mixed with known copy numbers of UGV-1 S segments (500 or 50.000 copies/lane). The cycling condition was: 1. initial denaturation at 98 °C for 10 s, 2. denaturation at 98 °C for 1 s, 3. annealing at 58 °C for 2 s, 4. extension at 72 °C for 3 s, with steps 2 to 4 repeated 38 times until final extension at 72 °C for 1 min. The tested primers were F1-5 and R1-5, each one of them at the following final concentrations: (**a**) 0.25 µM; (**b**) 0.25 µM F1-3, R1-2 and R4, and 0.5 µM F4-5, R3 and R5; (**c**) 0.25 µM F1-3, R1-2 and R4, and 1 µM F4-5, R3 and R5; (**d**) 0.3 µM; (**e**) 0.4 µM; (**f**) 0.5 µM; (**g**) 0.6 µM; (**h**) 0.7 µM and (**i**) 0.8 µM. The multiplex RT-PCR products were separated by agarose gel electrophoresis and the bands visualized under UV light by GelRed nucleic acid staining (Biotium, Fremont, CA, USA) pre-cast to the gels. Specific amplicons are at approximately 140 bp and are indicated by arrows. In each panel the images on the right represent the gels imaged at higher exposure times than on the left. Bands that became visible after higher exposure times are indicated by an *. Primer dimer formation, as bands at approximately 50 bp, becomes clear when the concentration of each primer is > 0.5 µM. Short spaces between gel lanes represent separate regions in the same gels, whereas longer spaces between gel lanes represent areas from different gels. Ladder: GeneRuler 100 bp plus DNA ladder (Thermo Fisher Scientific, Waltham, MA, USA); RT: reverse transcription; NTC: no template control; SNT: supernatant. The obtained results are also summarized in Table S3.

**Figure S3.** **Checking the multiplex RT-PCR results by transferring the reptarenavirus infection from
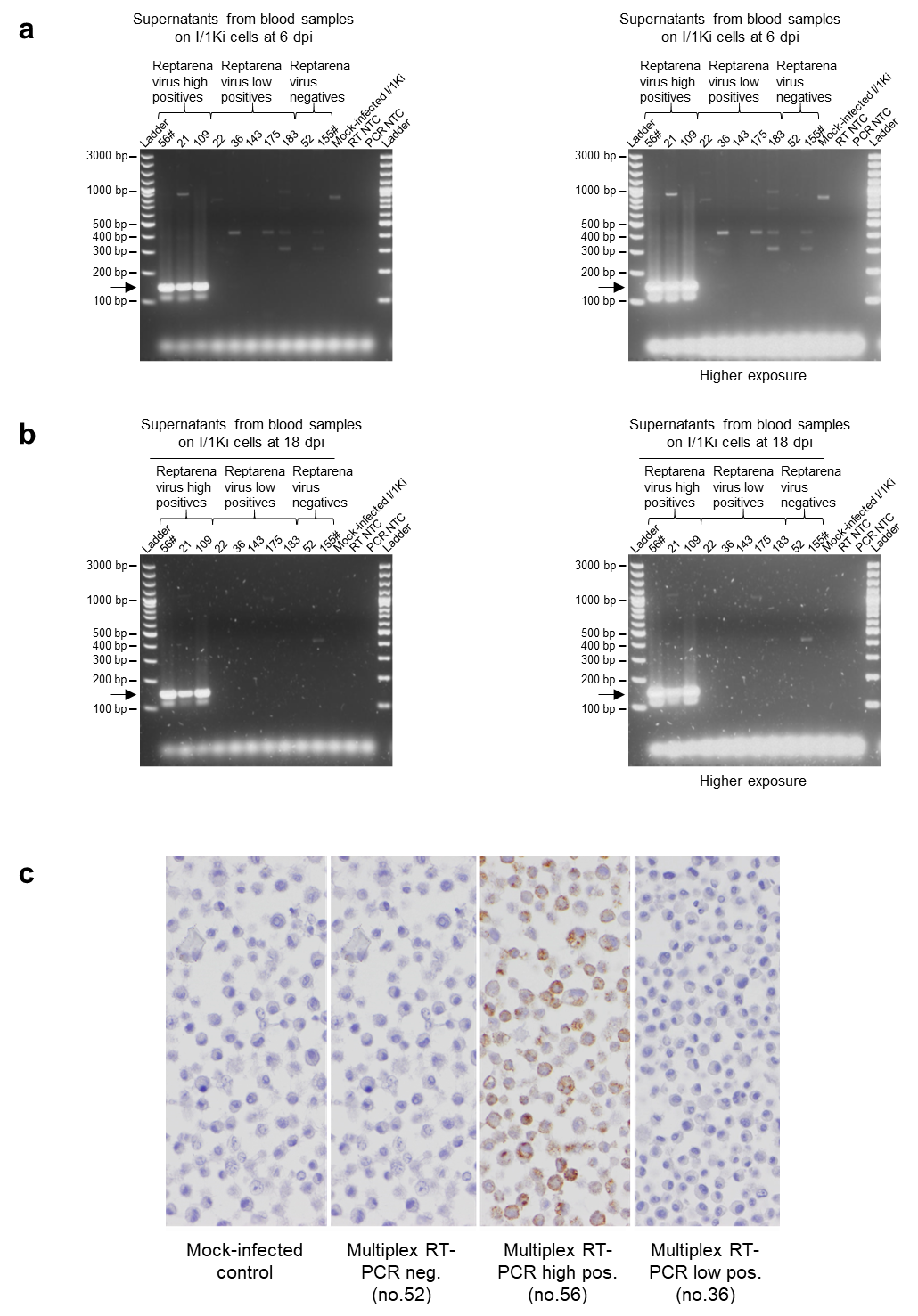
snake blood samples to a cell culture model.**

**Legend:** Aliquots (approximately 200 µl) of a subset of the snake bloods from the collection described in Thiele et. al., 2023 [9] were individually applied on boid I/1Ki cells, permissive for reptarenaviruses. The blood samples were derived from: two BIBD-positive animals (nos. 21 and 56), one of those with the highest viral load (no. 56), one BIBD-negative reptarenavirus carrier with high viral load (no.109), five BIBD-negative reptarenavirus carriers with low viral load (nos. 22, 36, 143, 175 and 183), and two BIBD- and reptarenavirus-negative samples (nos. 52 and 155). Afterwards, RNA isolation, cDNA synthesis and multiplex RT-PCR were performed on cell culture supernatants collected after (**a**) 6 or (**b**) 18 dpi. A cell culture supernatant from cells that were not incubated with any blood sample (Mock-infected I/1Ki) was analyzed as negative control. The multiplex RT-PCR products were separated by agarose gel electrophoresis and the bands visualized under UV light by GelRed nucleic acid staining (Biotium, Fremont, CA, USA) pre-cast to the gels. Specific amplicons are at approximately 140 bp and are indicated by an arrow. In each panel the images on the right represent the gels imaged at higher exposure times than on the left. No new specific band becomes visible in higher exposure times in comparison to lower exposure times. Ladder: GeneRuler 100 bp plus DNA ladder (Thermo Fisher Scientific, Waltham, MA, USA); RT: reverse transcription; NTC: no template control.

(**c**) Immunocytological evaluation of cell pellets generated from cell cultures for the expression of reptarenavirus N protein yielded negative results for the mock-infected control cells, the multiplex RT-PCR negative samples (no. 52) and the multiplex-RT-PCR positive, BIBD-negative carriers with low viral load (no. 36); the BIBD-positive samples with high viral load (no. 56) exhibited viral antigen expression in the majority of cells. Pictures are exemplifying results of 16 dpi inoculations.

#: samples analyzed via next generation sequencing (NGS) and de novo assembly [9].
