# Supplementary material for: A Multiplex RT-PCR Method for the Detection of Reptarenavirus Infection
Source: Viruses. 2023 Nov 25;15(12):2313. doi: 10.3390/v15122313 (PMC10747477; doi:10.3390/v15122313)
Supplement: Supplementary file 1 [file viruses-15-02313-s001.zip › Supplementary Material/Supporting Information_Original gel images.pdf]

Supporting Information: original images for gels

Original gel images are here provided for the cropped gel data of Figure 1 a-e, Figure 2 a-e, Figure 3 a,b, Figure S1 a-h and Figure S2 a-i. Gel data of Figure 3 c, Figure 4, Figure 5 and Figure S3 already represent original, uncropped and unadjusted images in the main text and in the supplementary material and, therefore, are not reported in this section except higher exposure images of Figures 4 and 5.

Original gel images of Figure 1 a, b and c

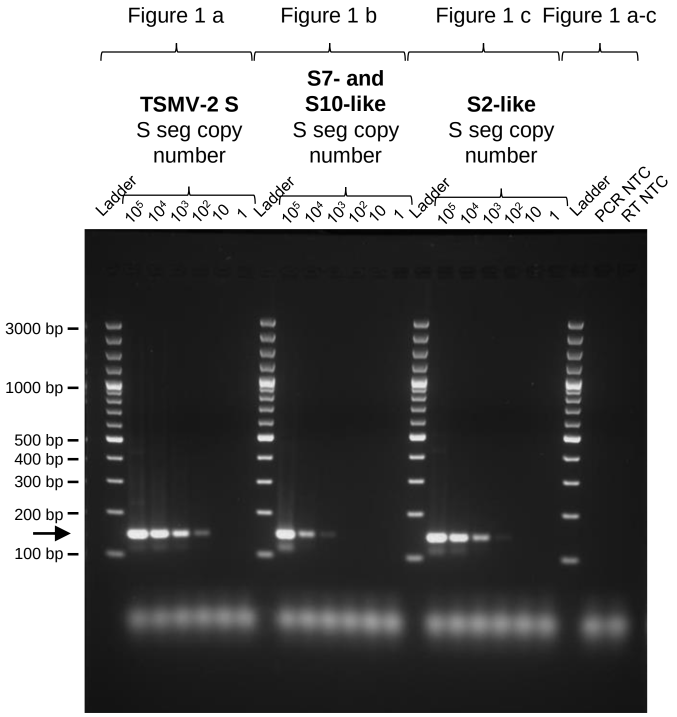

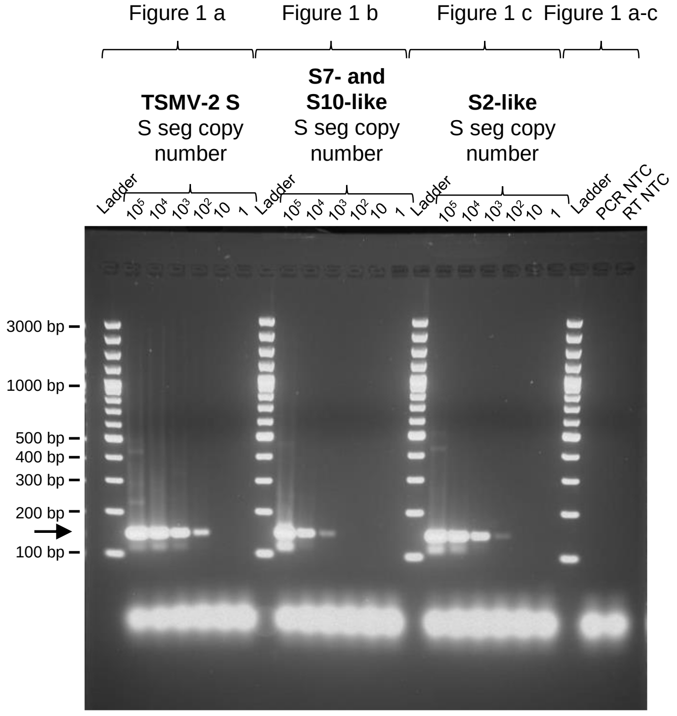

Higher exposure

Original gel images of Figure 1 d and e, Figure 2 a

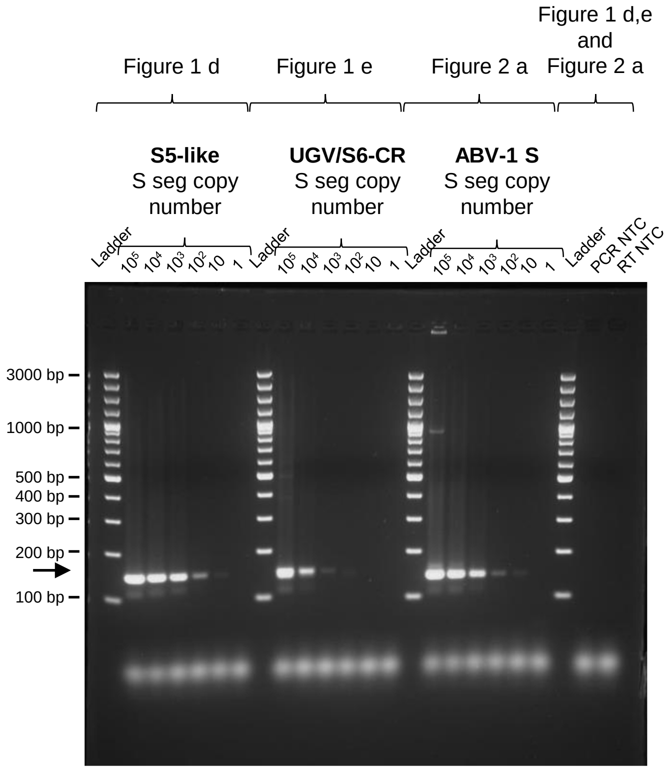

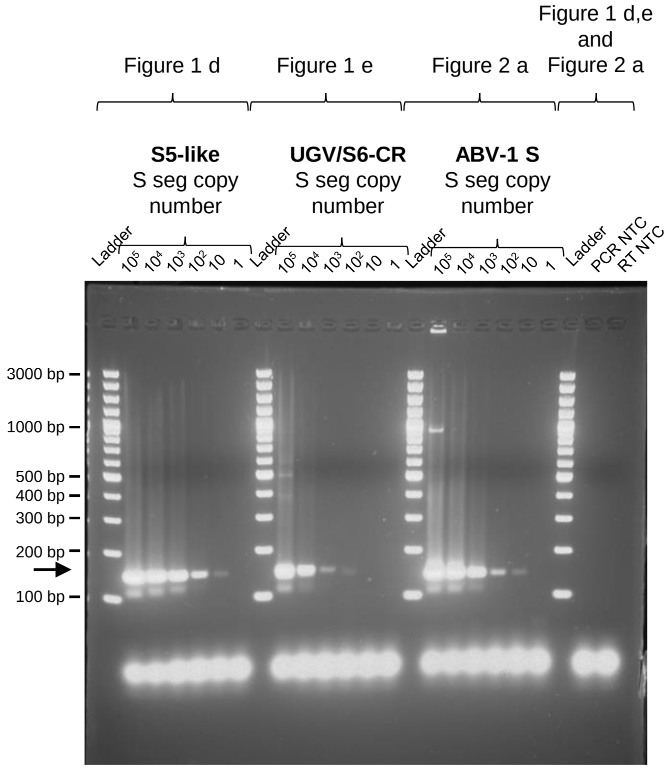

Higher exposure

Original gel images of Figure 2 b and c

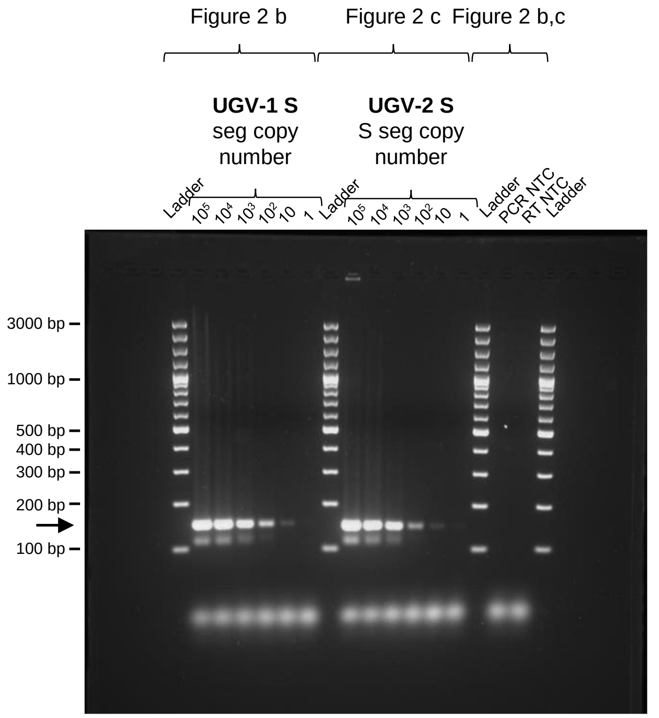

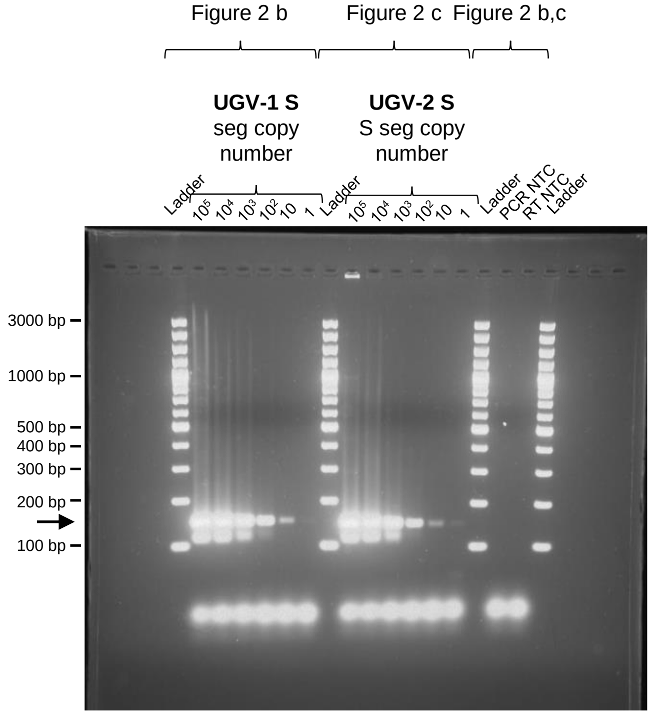

Higher exposure

Original gel images of Figure 2 d and e

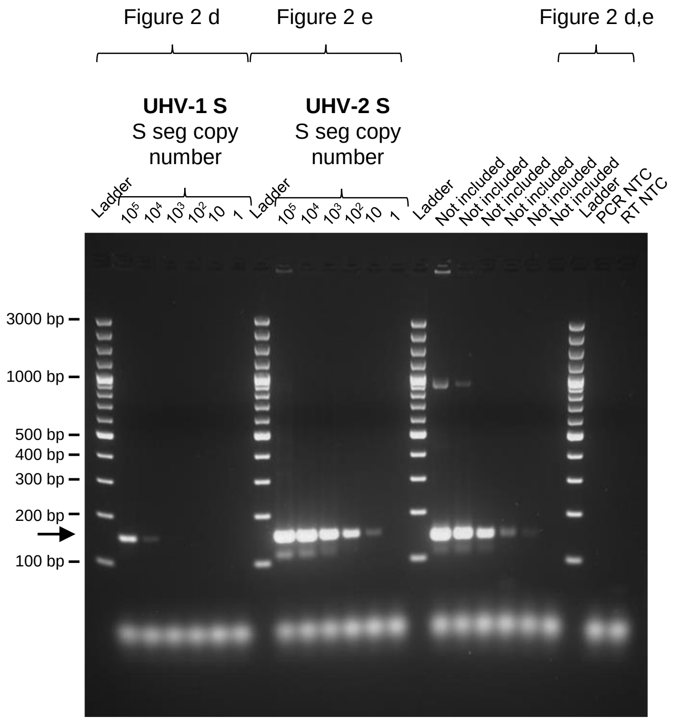

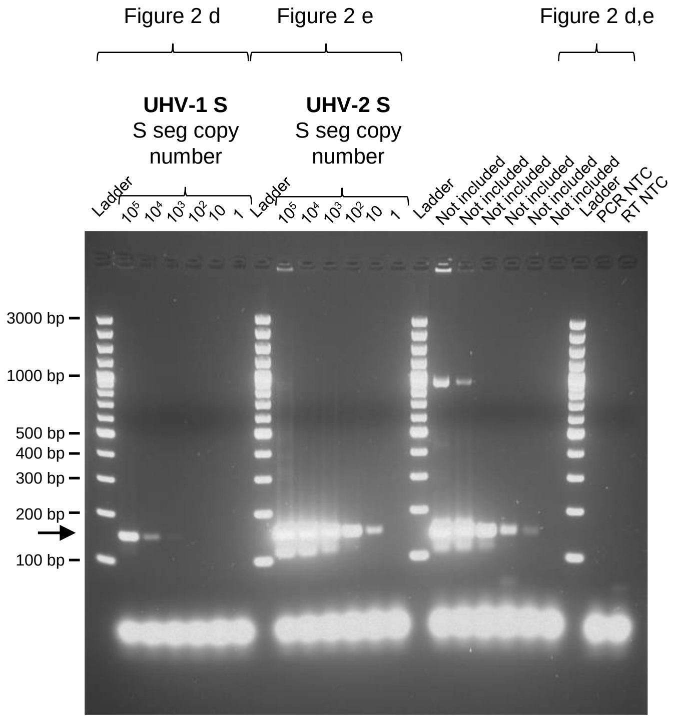

Higher exposure

Original gel images of Figure 3 a and b

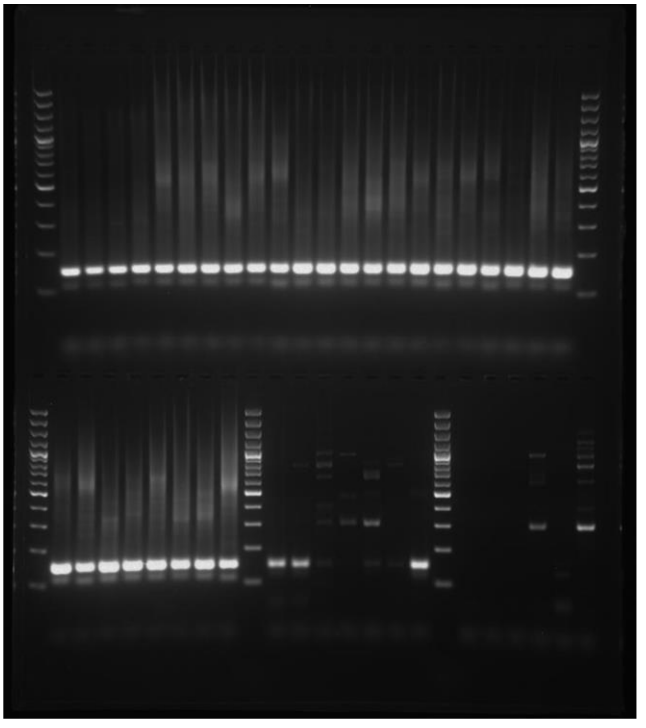

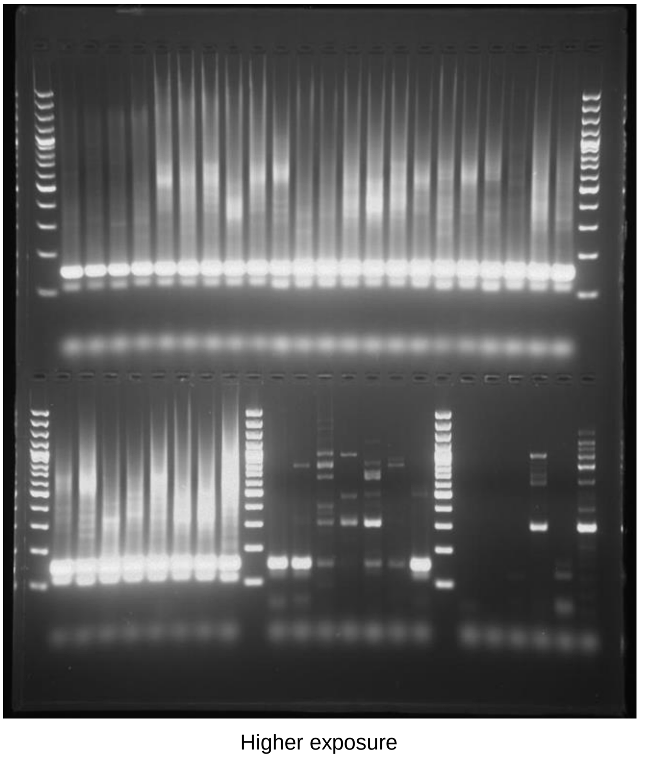

Higher exposure

Gel description provided in the next two pages

Original gel images of Figure 3 a

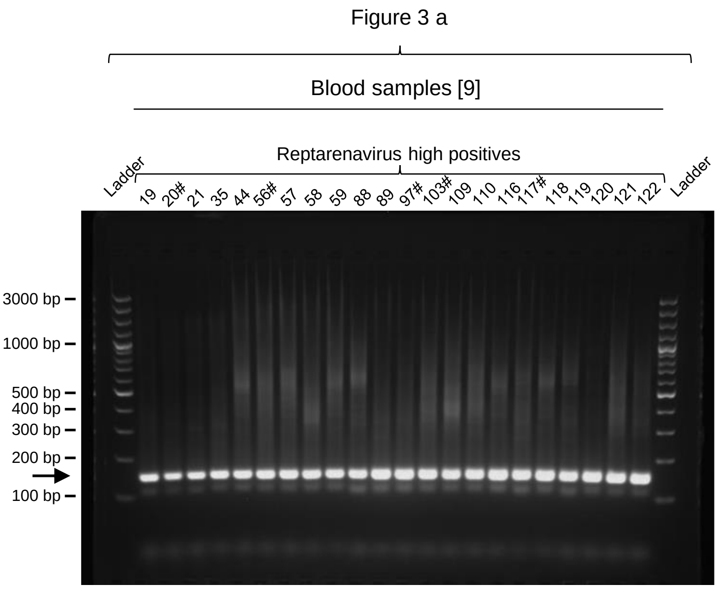

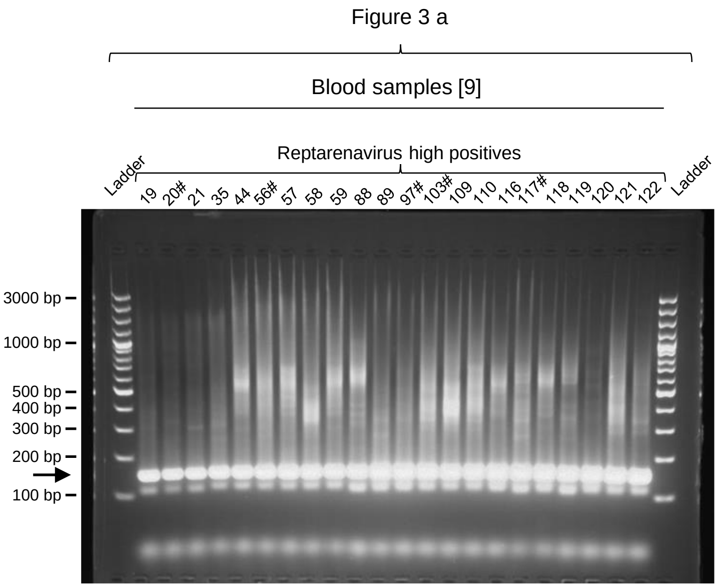

Higher exposure

Original gel images of Figure 3 b

Figure 3 b

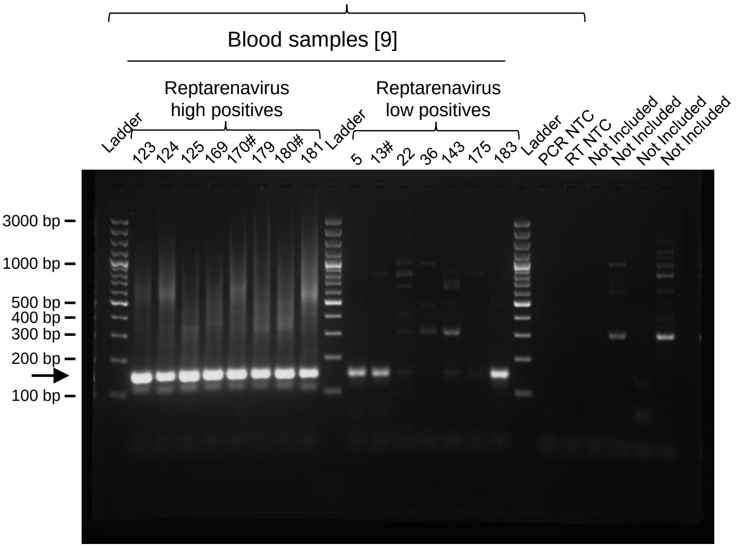

Figure 3 b

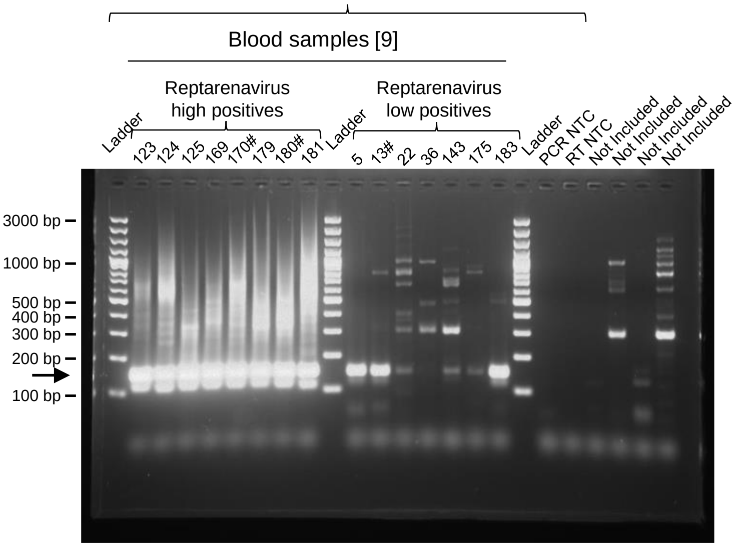

Higher exposure

Original higher exposure image of Figure 4

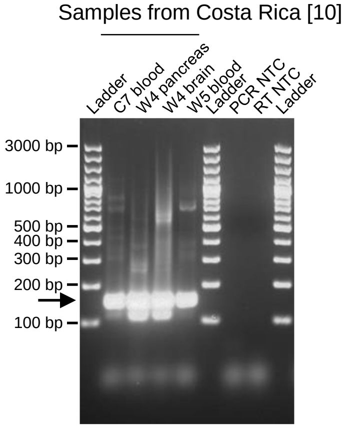

Higher exposure

Original higher exposure image of Figure 5

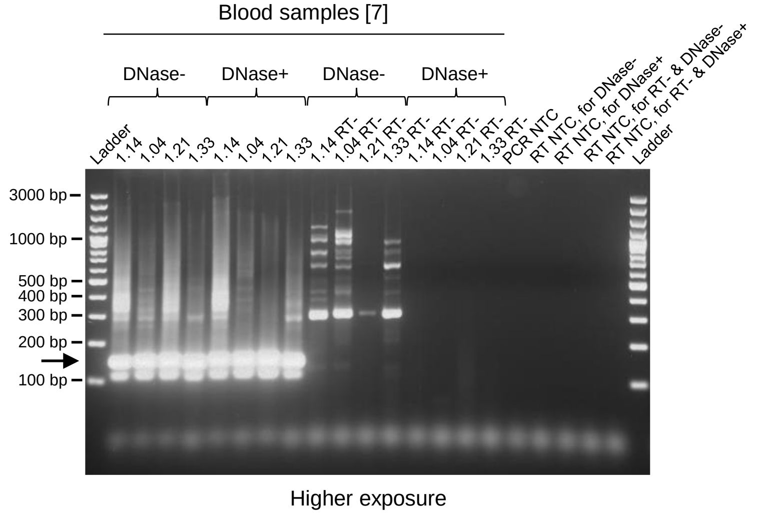

Higher exposure

Original gel images of Figure S1 a-d

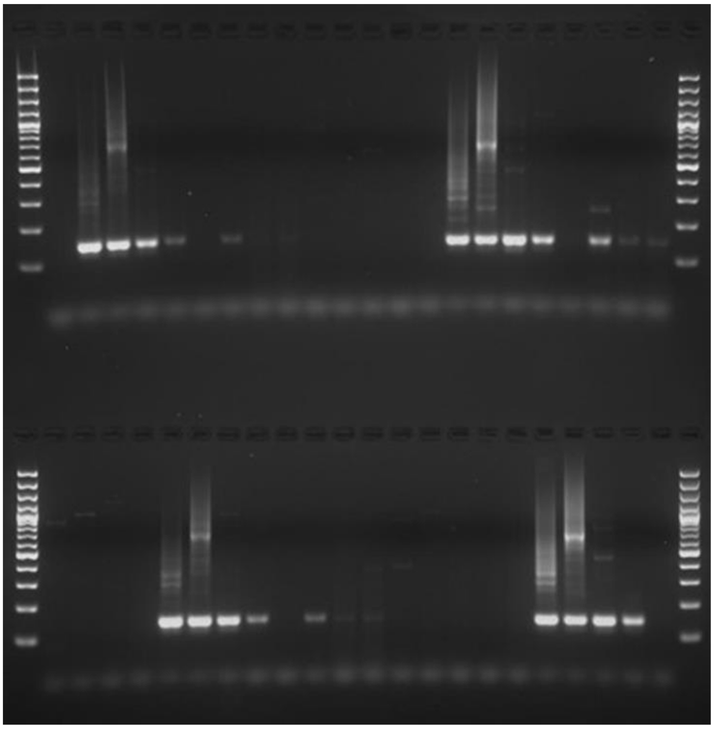

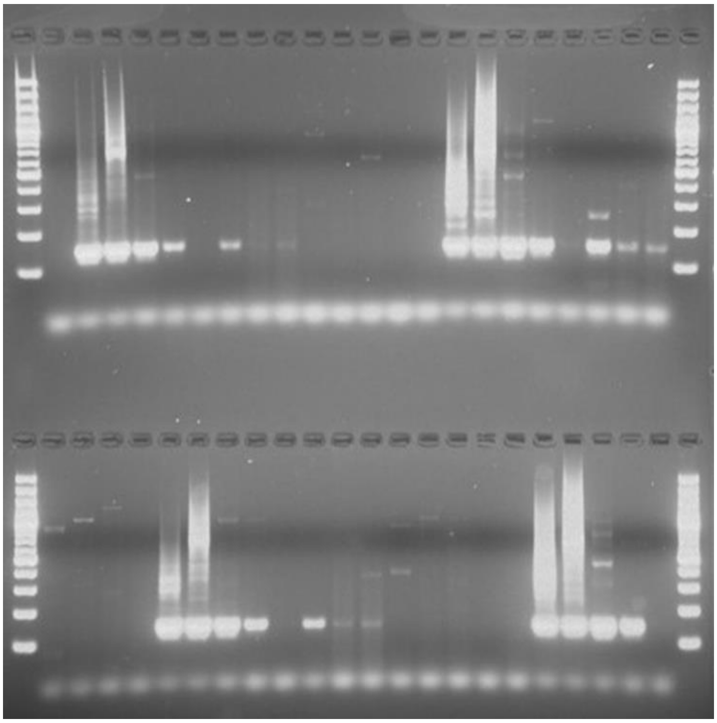

Higher exposure

Gel description provided in the next two pages

Original gel images of Figure S1 a and b

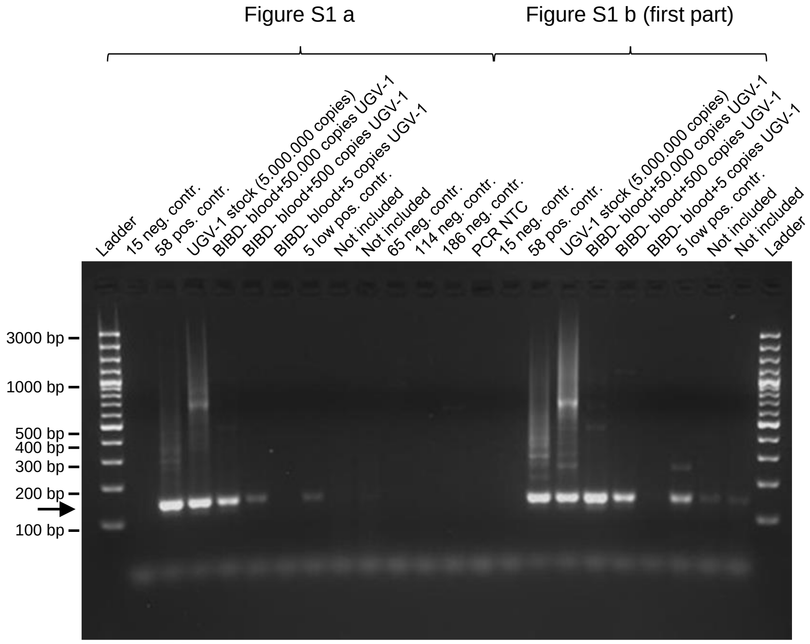

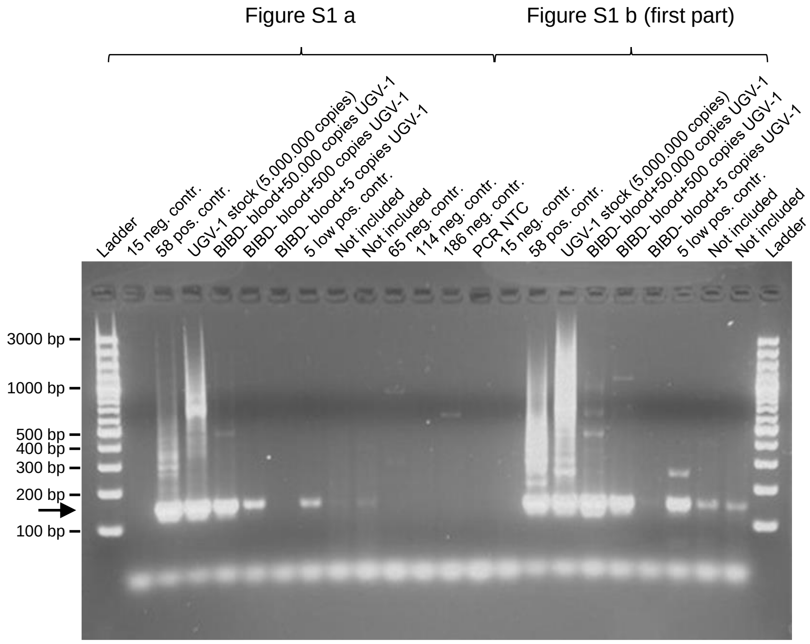

Higher exposure

Original gel images of Figure S1 b, c and d

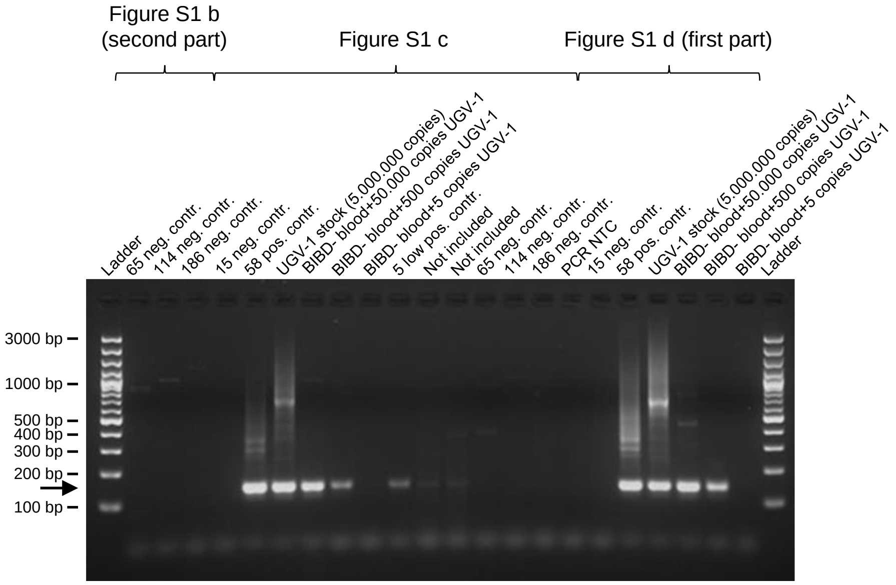

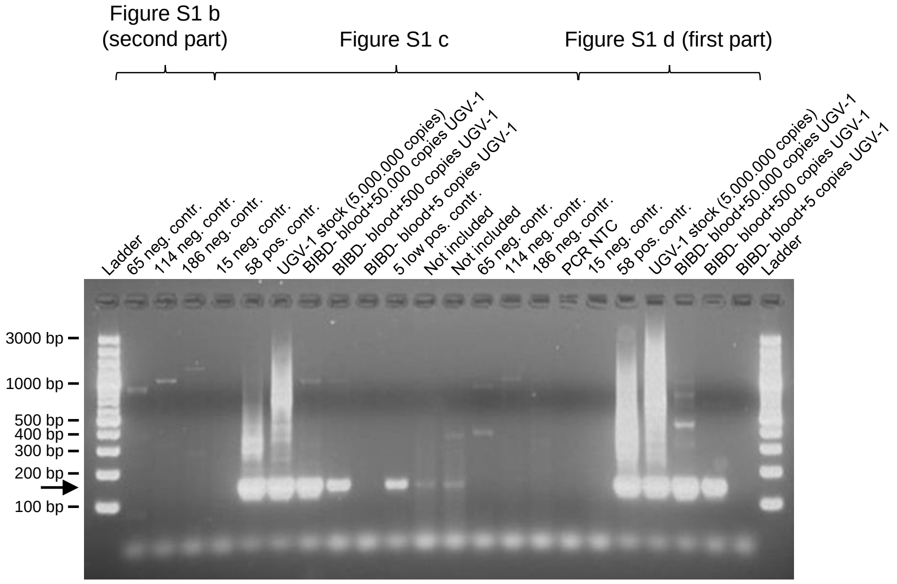

Higher exposure

Original gel images of Figure S1 d-g

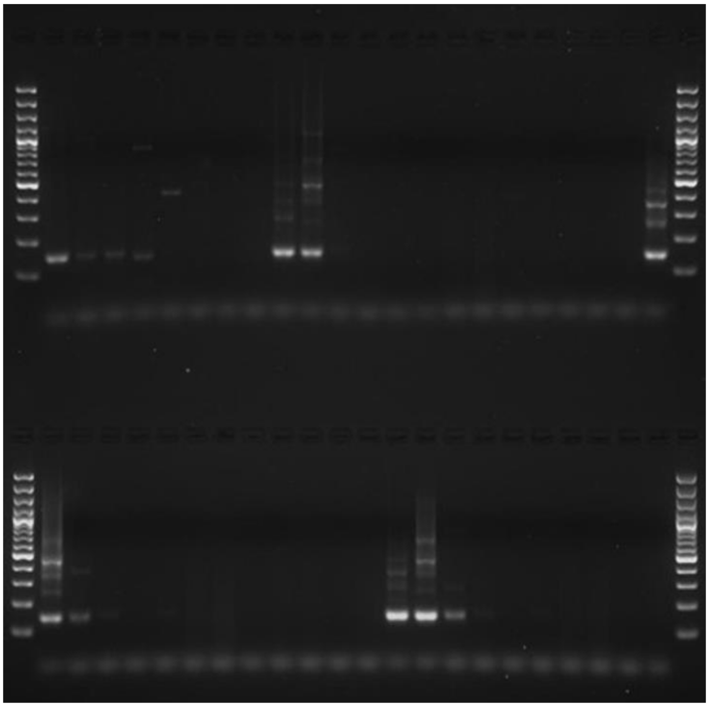

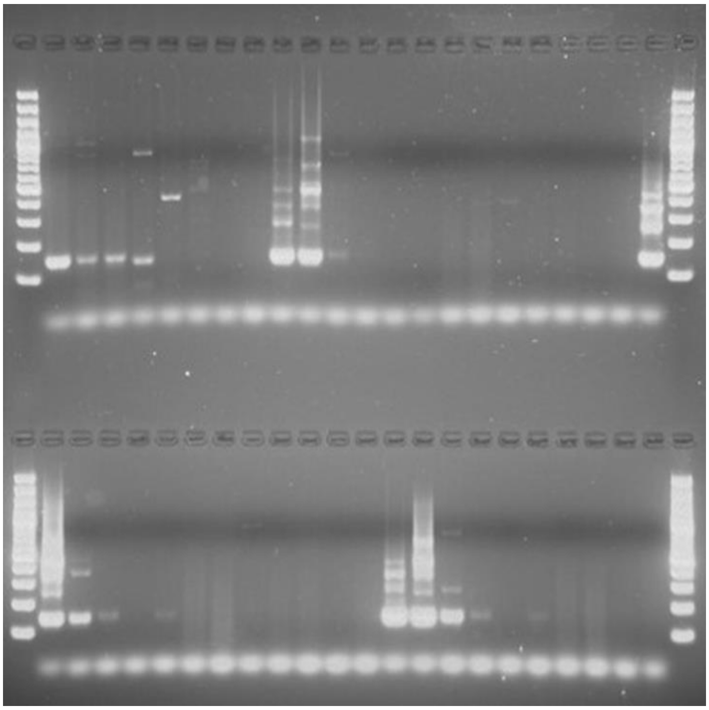

Higher exposure

Gel description provided in the next two pages

Original gel images of Figure S1 d, e and f

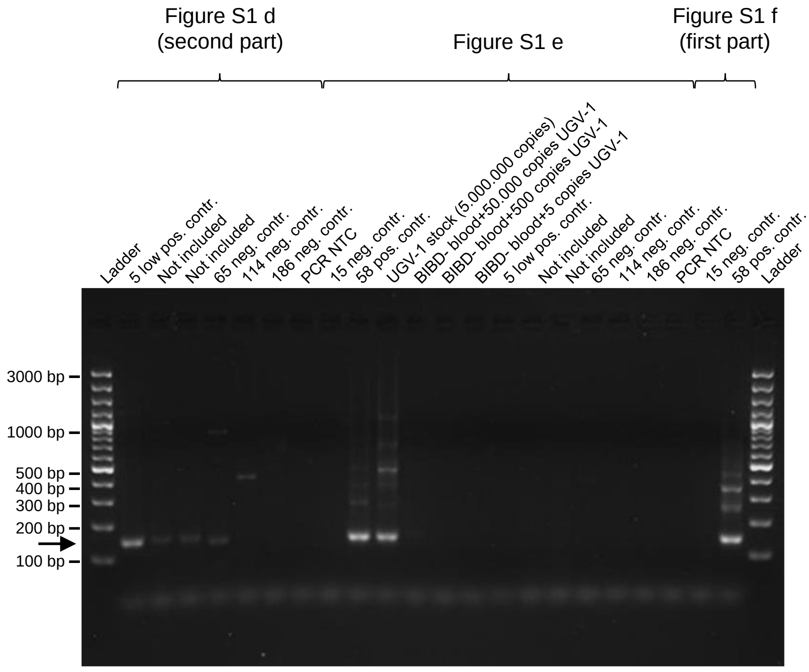

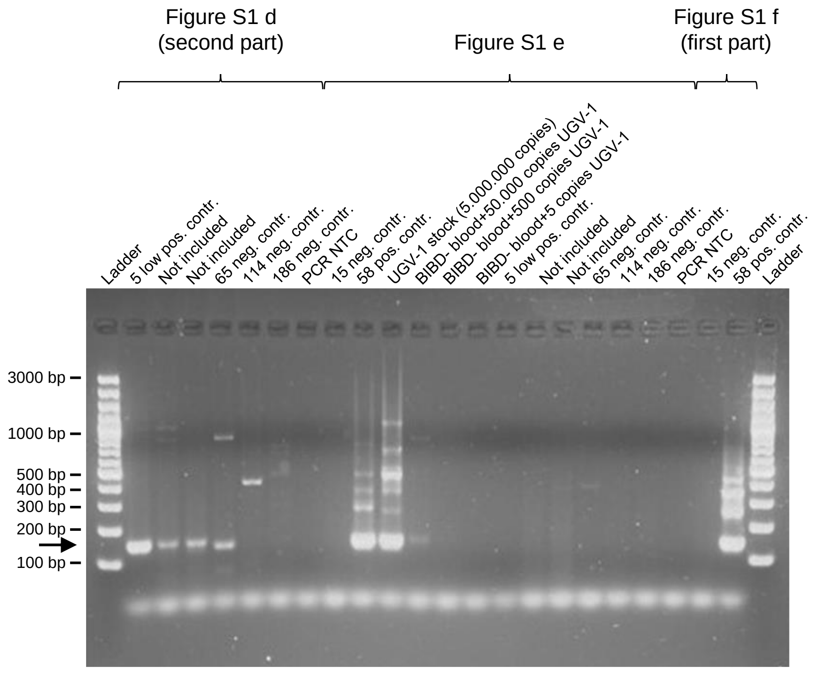

Higher exposure

Original gel images of Figure S1 f and g

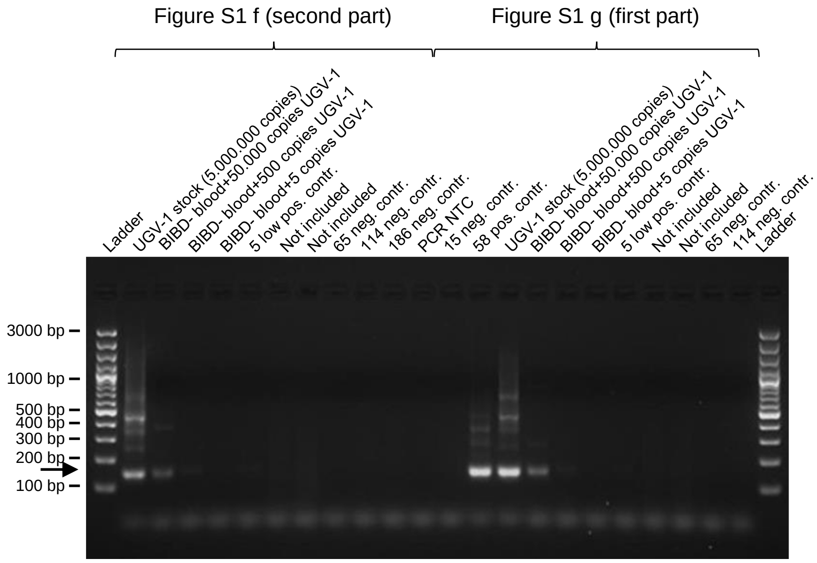

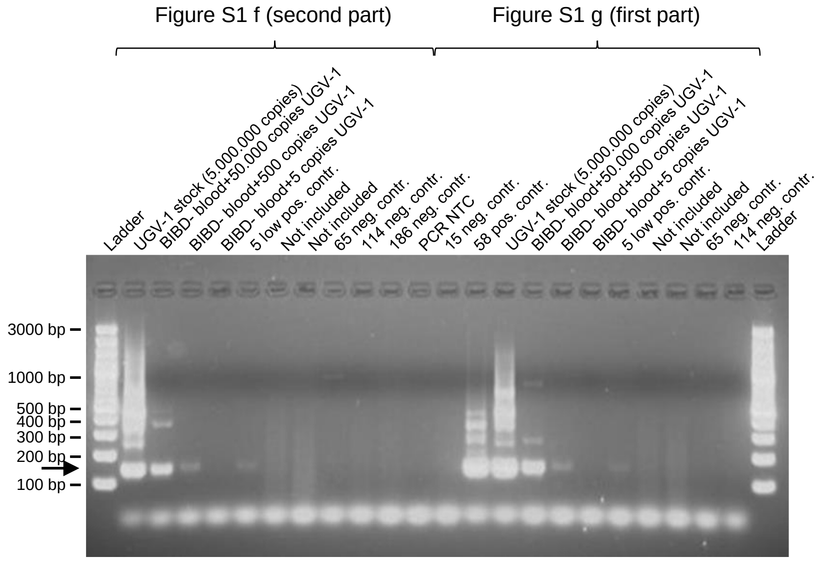

Higher exposure

Original gel images of Figure S1 g and h

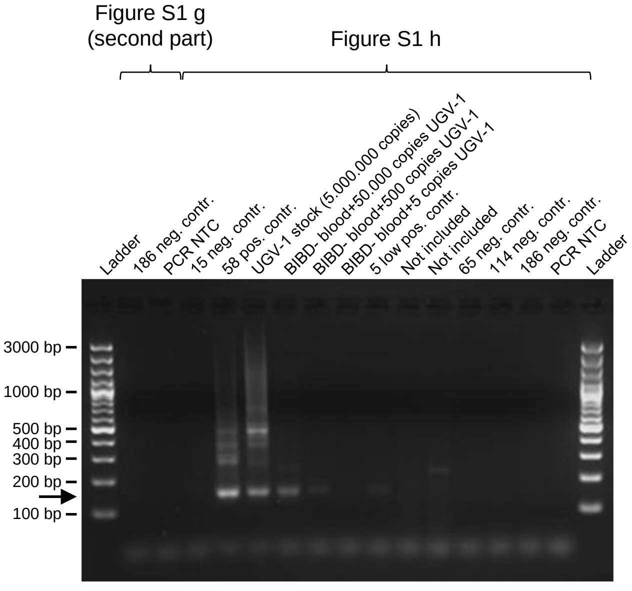

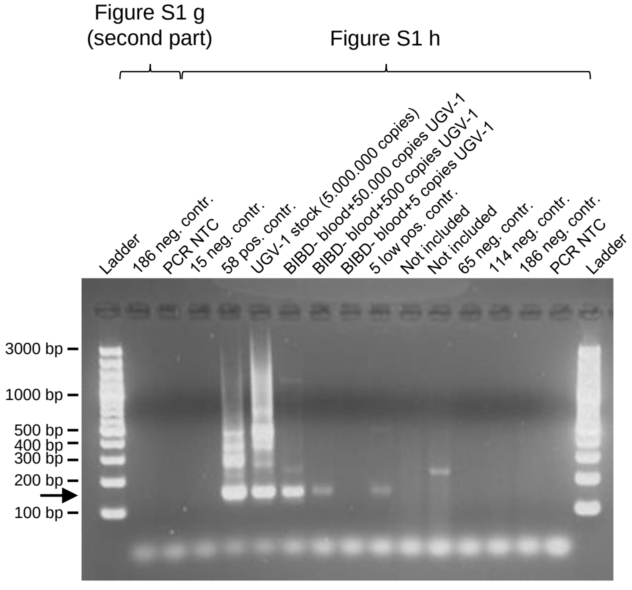

Higher exposure

Original gel images of Figure S2 a and f

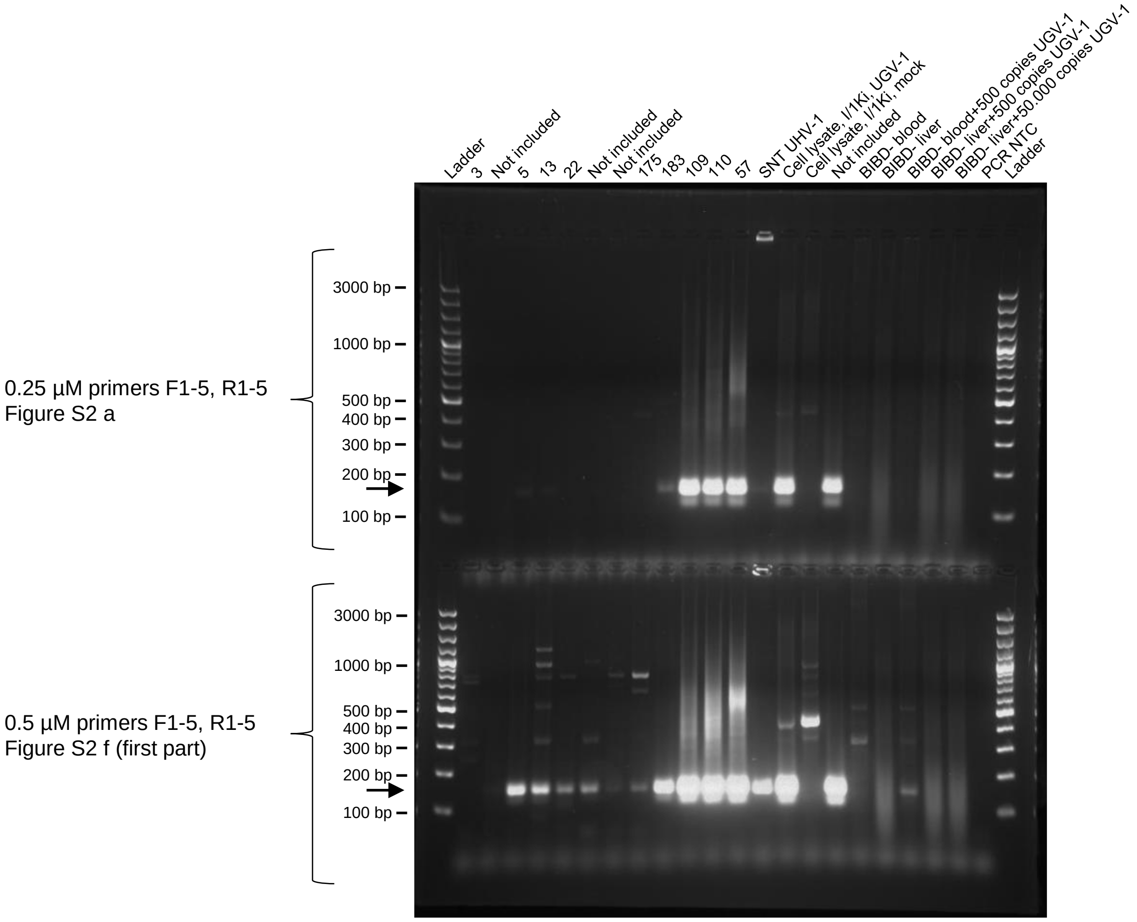

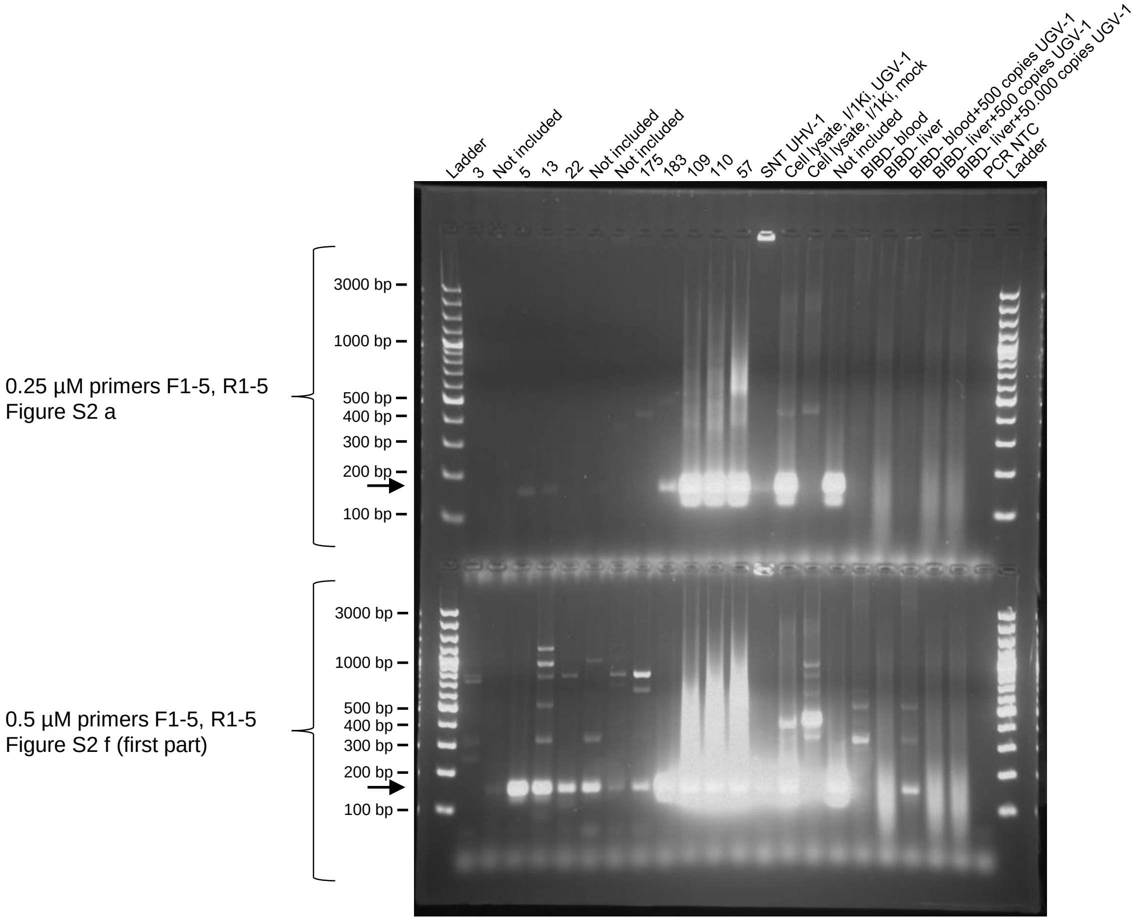

Higher exposure

Original gel images of Figure S2 b and c

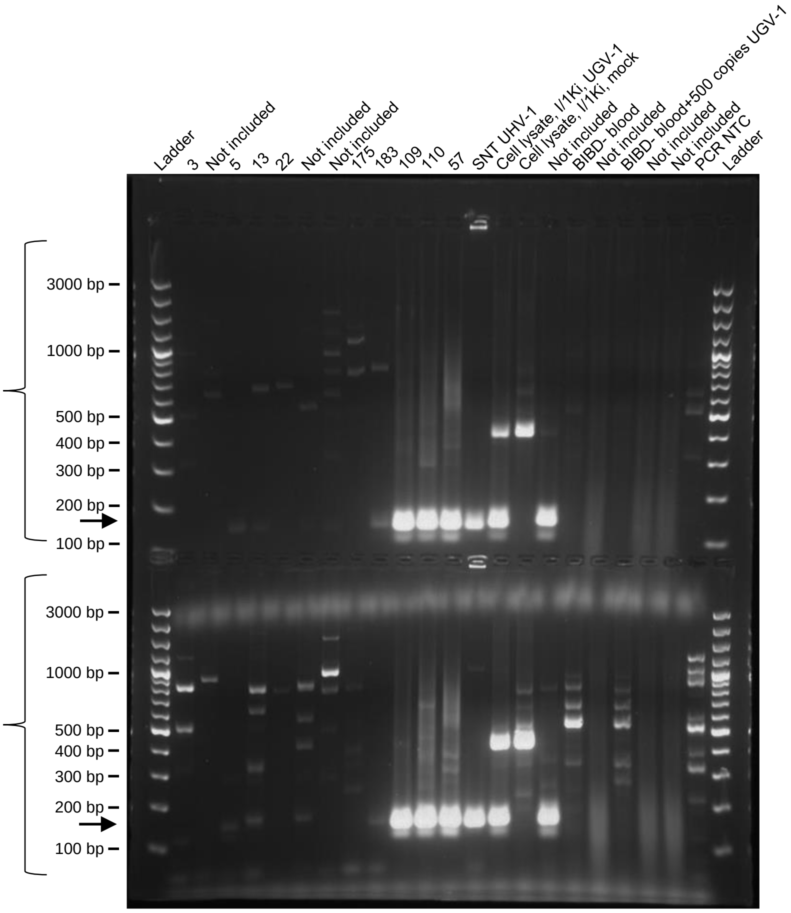

0.25 µM primers F1-3, R1-2, R4, and 0.5 µM F4-5, R3 and R5
Figure S2 b

0.25 µM primers F1-3, R1-2, R4, and 0.5 µM F4-5, R3 and R5
Figure S2 c

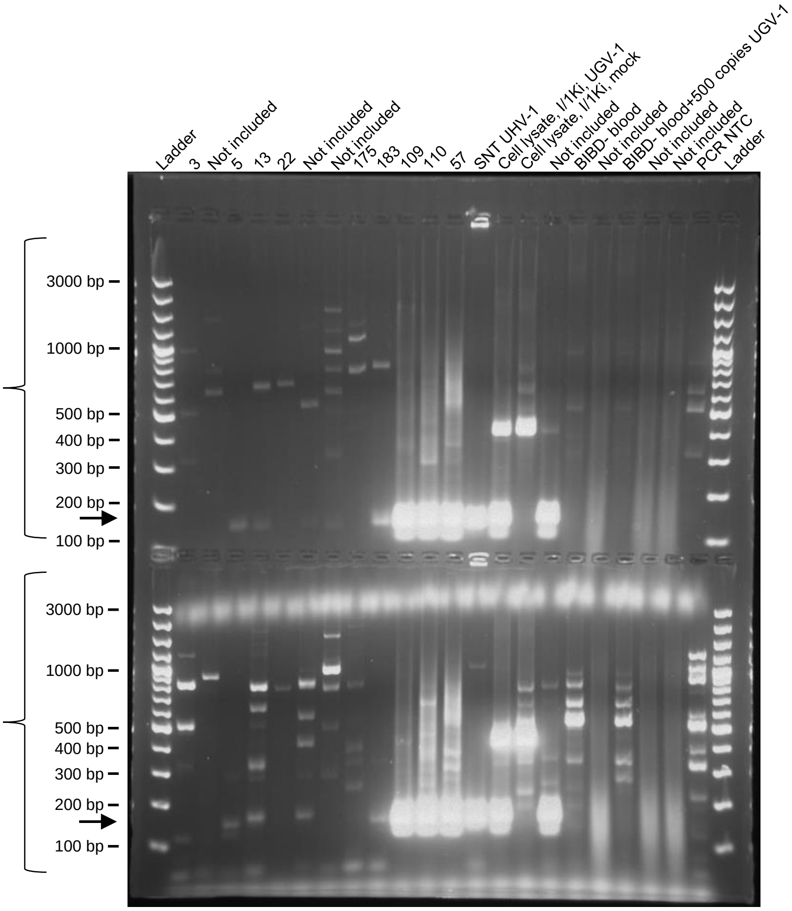

0.25 µM primers F1-3, R1-2, R4, and 0.5 µM F4-5, R3 and R5
Figure S2 b

0.25 µM primers F1-3, R1-2, R4, and 0.5 µM F4-5, R3 and R5
Figure S2 c

Higher exposure

Original gel images of Figure S2 d and e

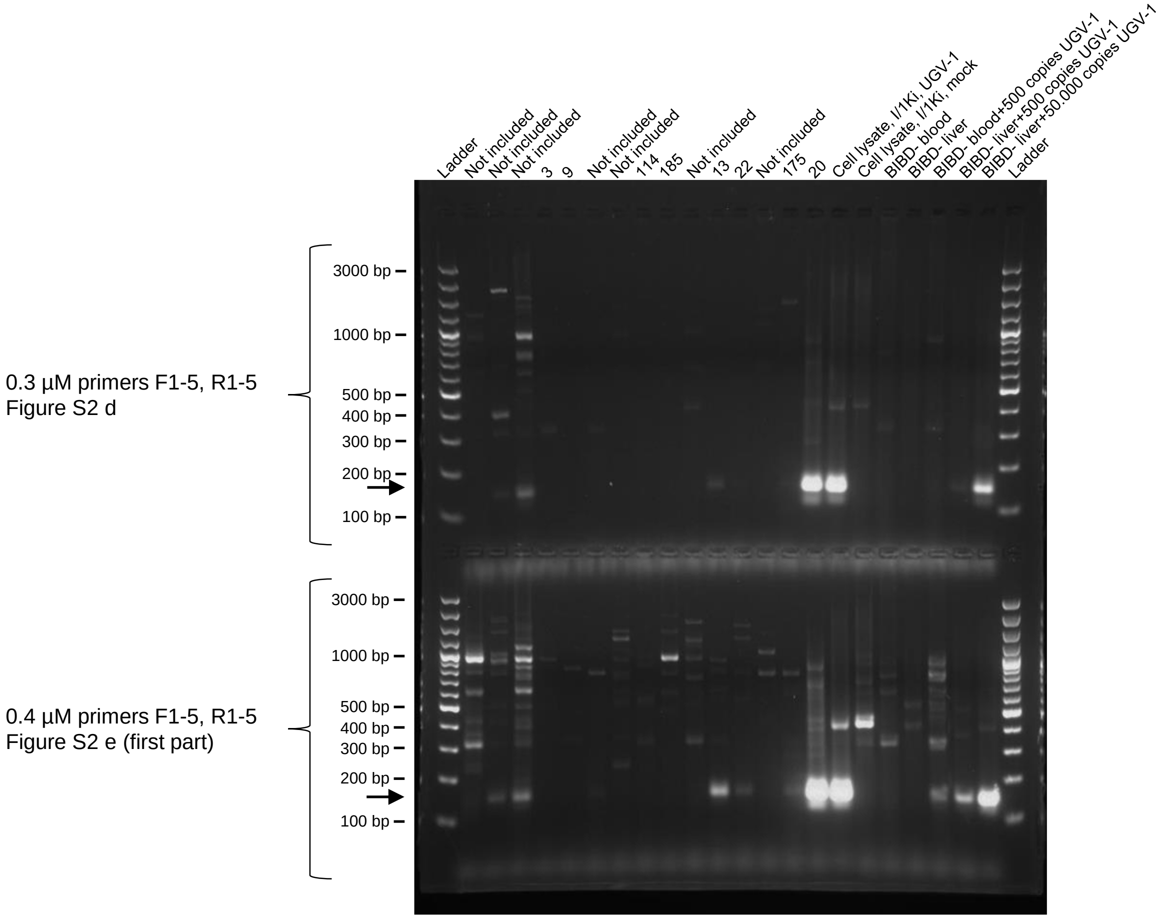

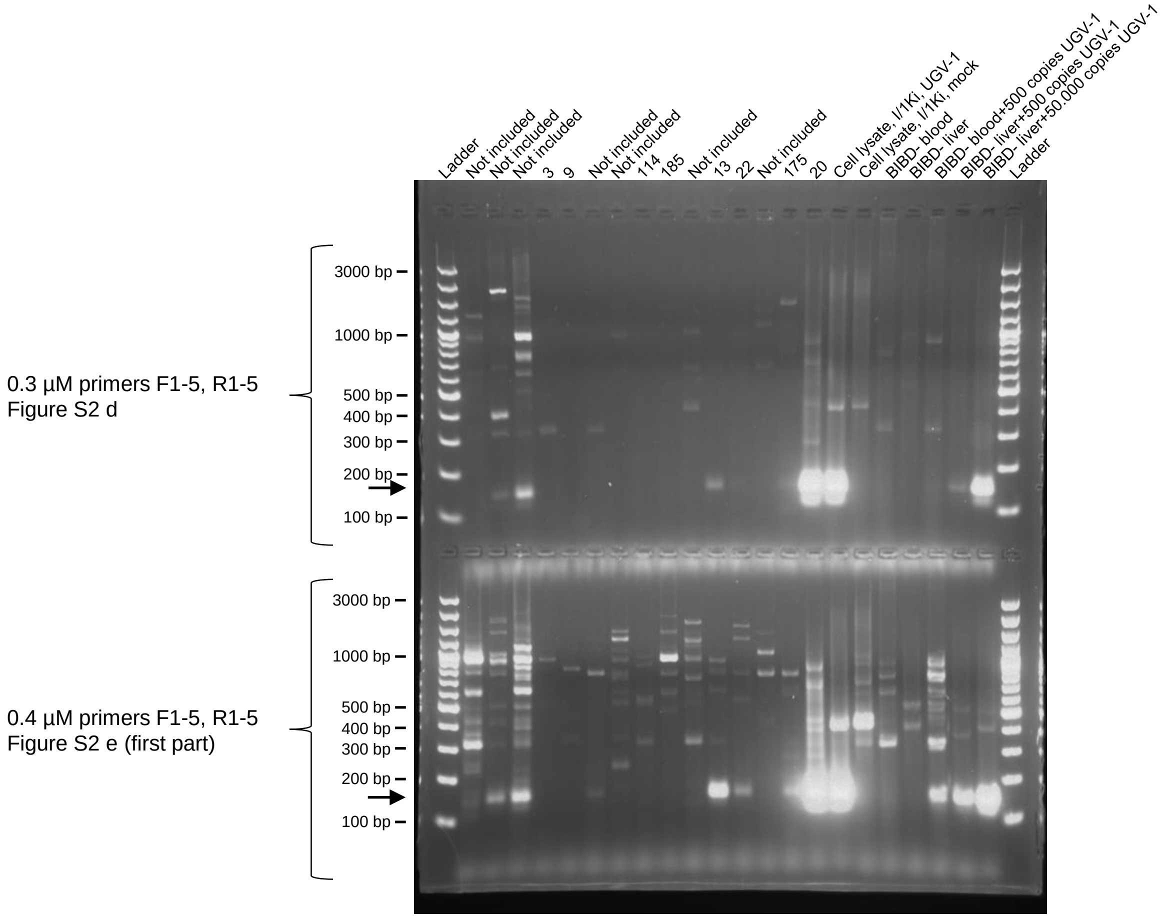

Higher exposure

Original gel images of Figure S2 f, and of PCR NTC of Figure S2 d, e, g, h and i

0.5 µM primers F1-5, R1-5
Figure S2 f (second part)
and PCR NTC of Figure S2
d, e and g-i

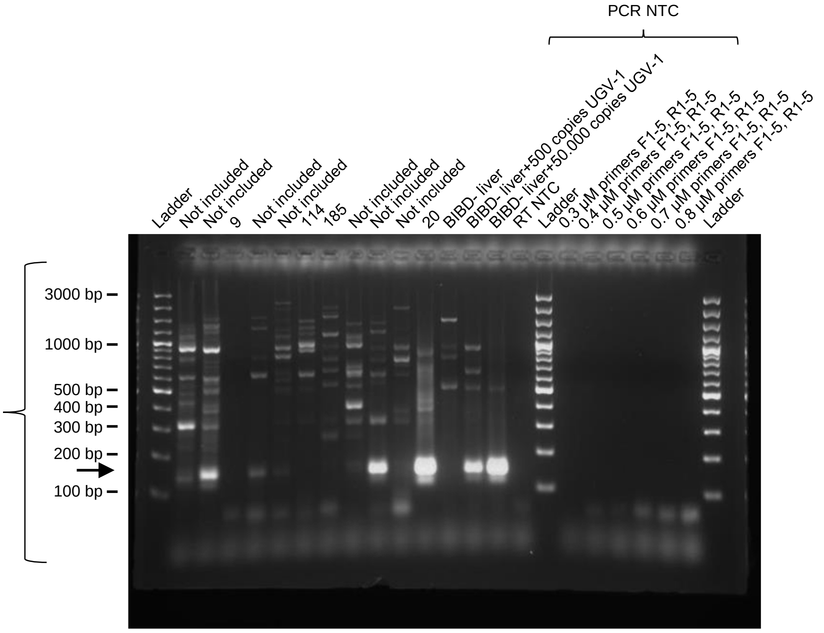

0.5 µM primers F1-5, R1-5
Figure S2 f (second part)
and PCR NTC of Figure S2
d, e and g-i

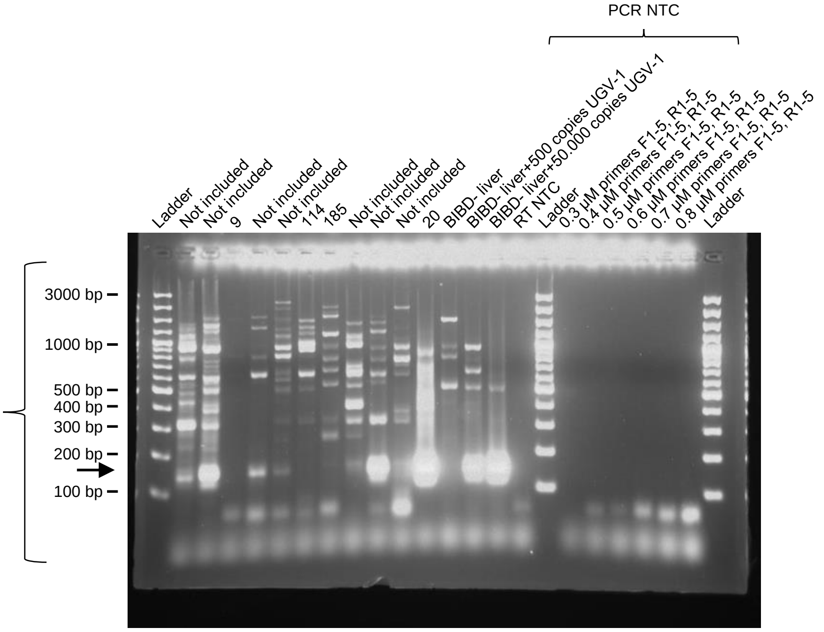

Higher exposure

Original gel images of Figure S2 g

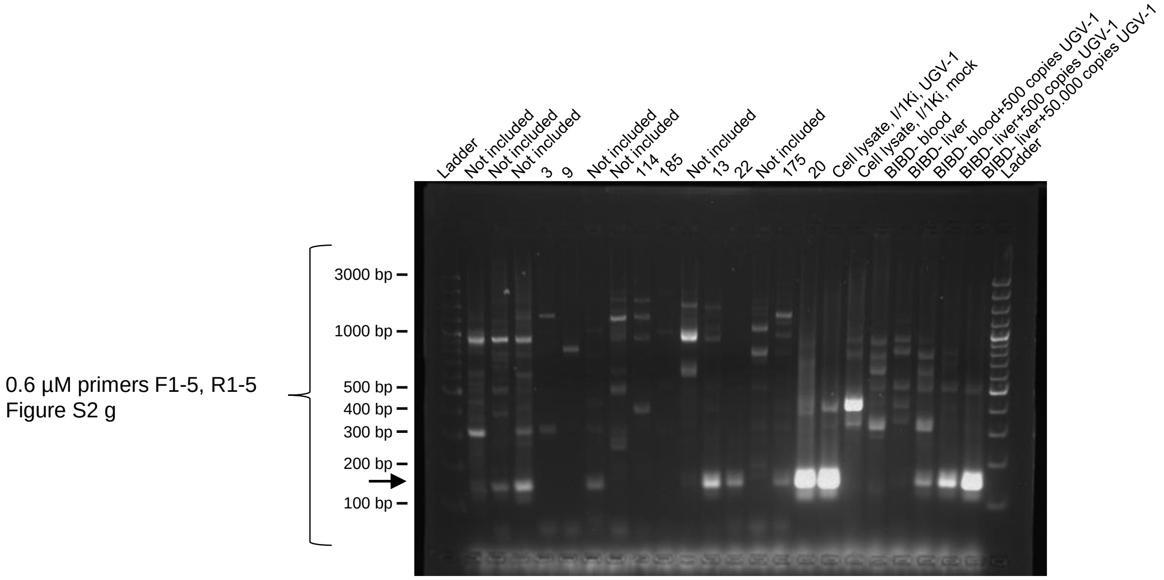

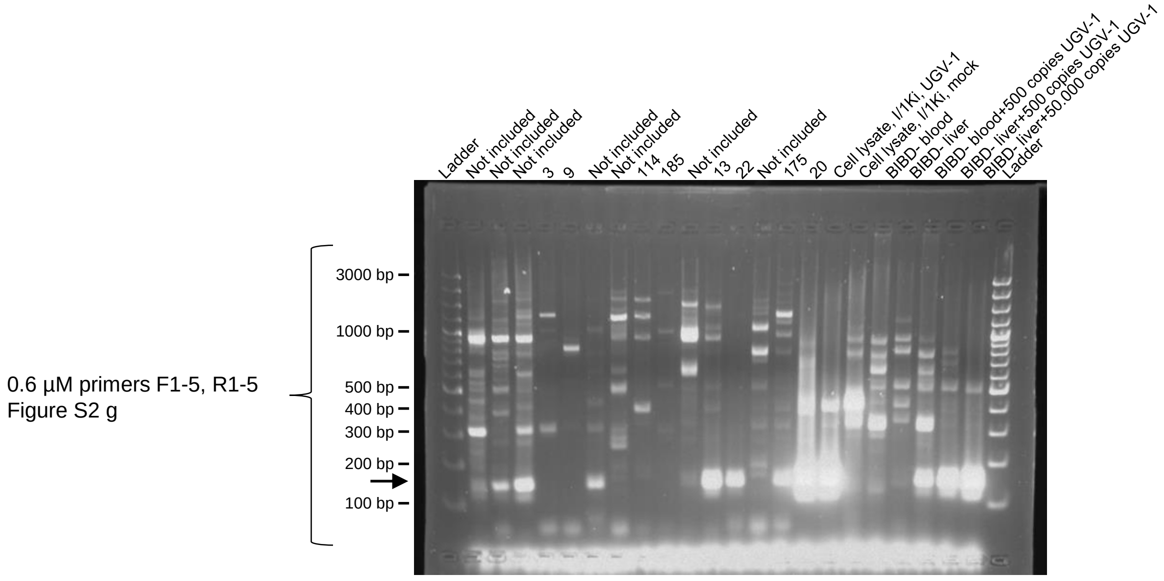

Higher exposure

Original gel images of Figure S2 h and i

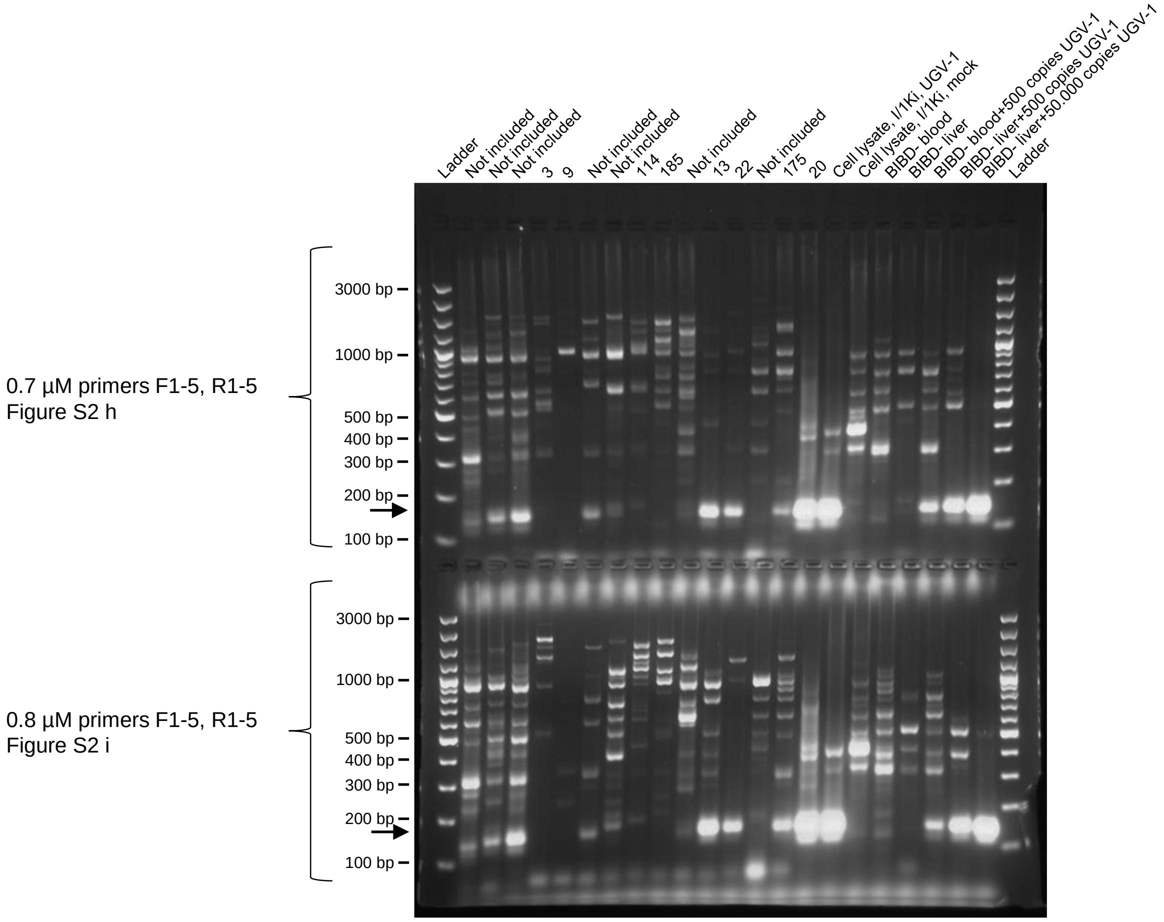

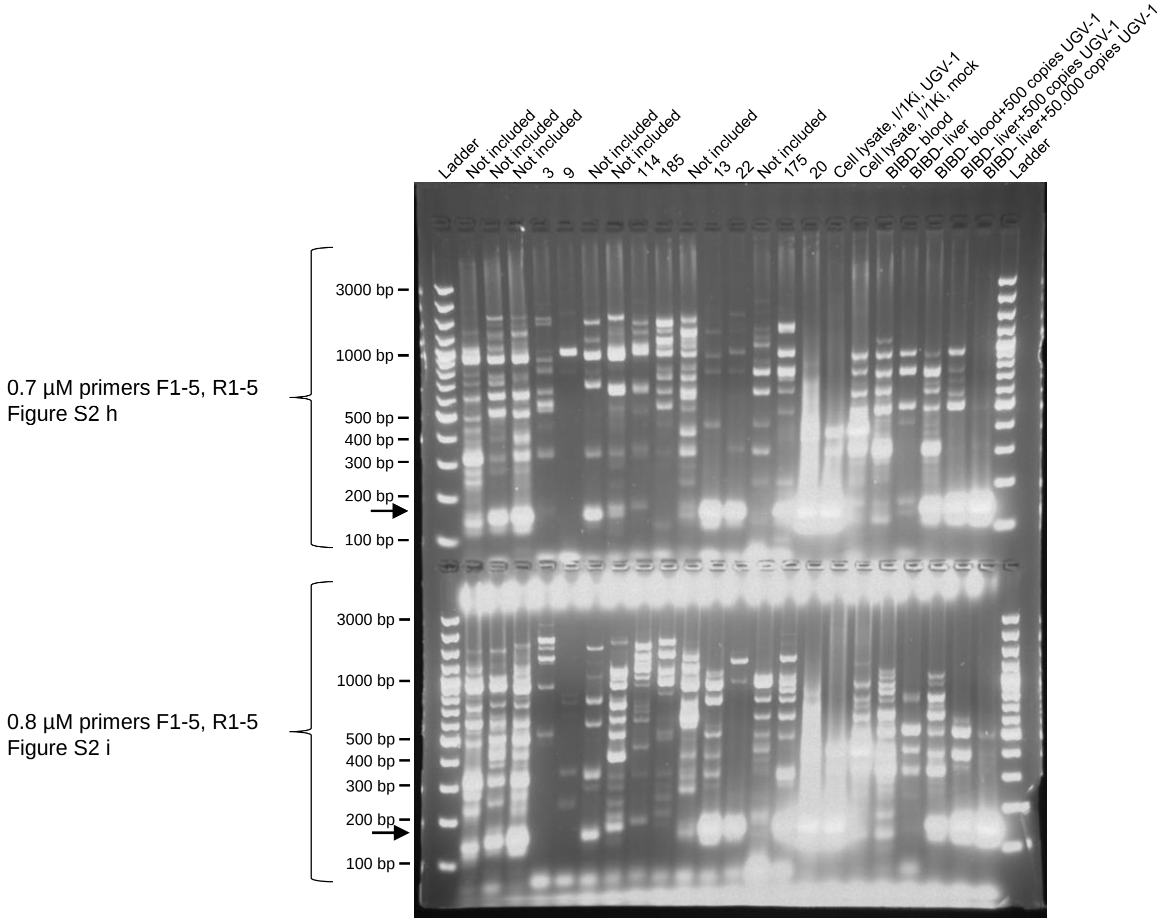

Higher exposure

Original gel images of Figure S2 e

0.4 µM primers F1-5, R1-5
Figure S2 e (second part)

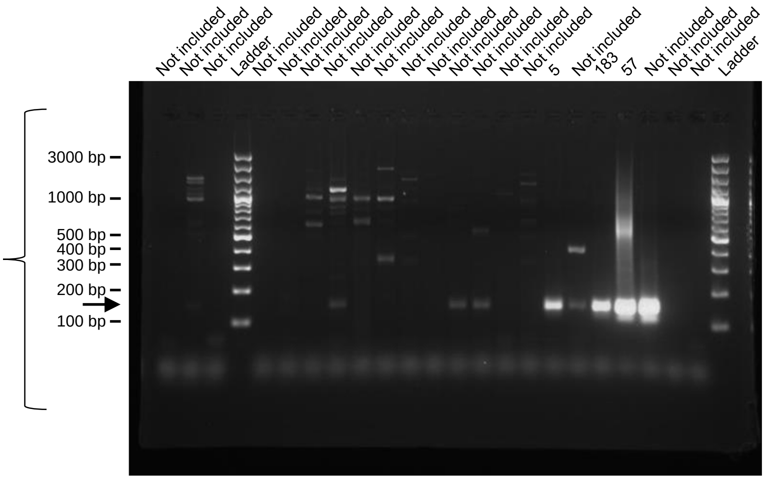

0.4 µM primers F1-5, R1-5
Figure S2 e (second part)

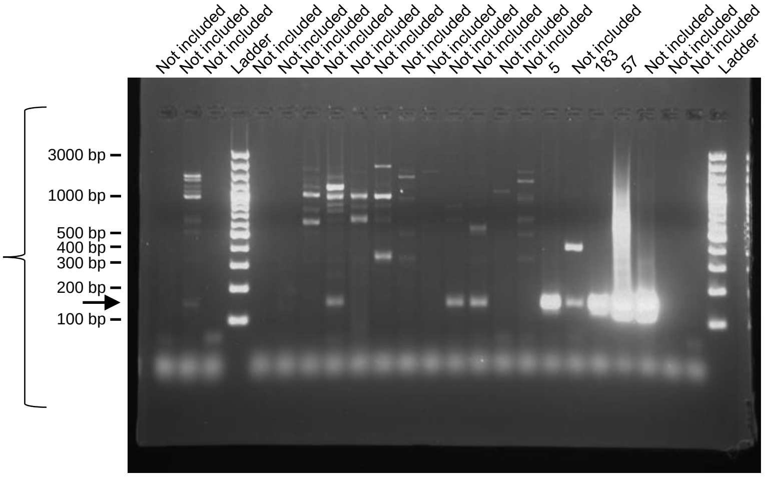

Higher exposure

Original gel images of Figure S2 d, e, g, h and i

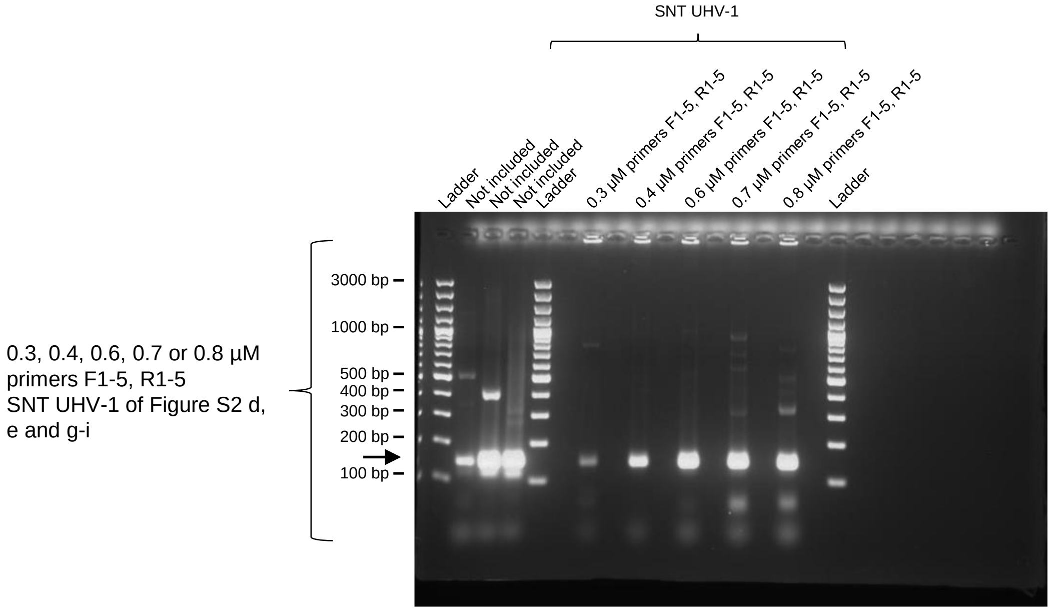

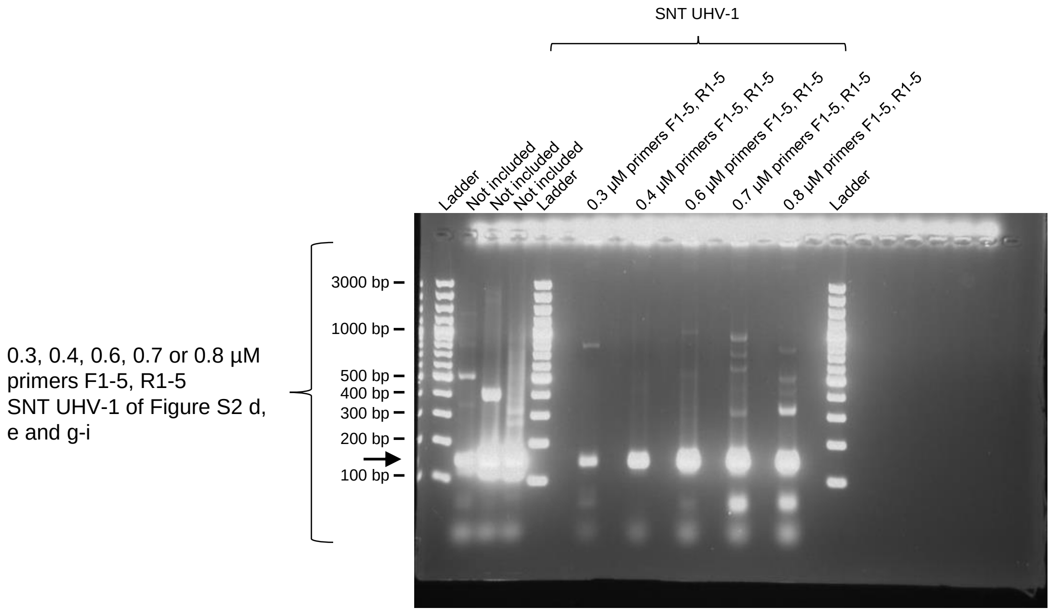

Higher exposure
