# Supplementary material for: A Multiplex RT-PCR Method for the Detection of Reptarenavirus Infection
Source: Viruses. 2023 Nov 25;15(12):2313. doi: 10.3390/v15122313 (PMC10747477; doi:10.3390/v15122313)
Supplement: Supplementary file 1 [file viruses-15-02313-s001.zip › Supplementary Material/Table S7a-c Stats.docx]

**Table S7**. Agreement between tests and sensitivity and specificity analysis for the multiplex RT-PCR.

**a.** Agreement between the different tests used in all the studies including [9], expressed as Cohen’s κ (95%CI)

|  | BIBD | NGS | qRT-PCR | S4 | S9 | S10 |
| --- | --- | --- | --- | --- | --- | --- |
| NGS (n) Cohen's κ, (95%CI) | 1.000, (15) (1.000 - 1.000) |  |  |  |  |  |
| RAV RT/qPPCR (n)  Cohen's κ, (95%CI) | 0.642, (253) (0.548 - 0.736) | 0.867, (15) (0.618 - 1.000) |  |  |  |  |
| S4 (n)  Cohen's κ, (95%CI) | 0.6878, (70) (0.524 - 0.852) | Not tested | 0.176, (70)  (0.020 - 0.332) |  |  |  |
| S9 (n)  Cohen's κ, (95%CI) | 0.1327, (70)  (-0.018 - 0.283) | Not tested | 0.582, (70) (0.262 - 0.903) | 0.105, (70)  (-0.088 - 0.299) |  |  |
| S10 (n)  Cohen's κ, (95%CI) | -0.125, (70)  (-0.319 - 0.068) | Not tested | 0.340, (70) (0.084 - 0.595) | -0.076, (70)  (-0.289 - 0.136) | 0.378, (70)  (0.113 - 0.642) |  |
| Multiplex RT-PCR (n)  Cohen's κ, (95%CI) | 0.740, (347) (0.665 - 0.814) | 0.867, (15) (0.618 - 1.000) | 0.991, (245) (0.975 - 1.000) | 0.212, (64) (0.029 - 0.395) | 0.636, (64) (0.315 - 0.958) | 0.358, (64) (0.093 - 0.622) |

κ: ≤0: poor agreement, 0.1 to 0.2: slight agreement, 0.21 to 0.4: fair agreement, 0.41 to 0.6: moderate agreement, 0.61 to 0.8: substantial agreement, 0.81 to 1.0: almost perfect agreement

**b.** Sensitivity and specificity analysis of UGV/S6 qRT-PCR and multiplex RT-PCR using the detection of cytoplasmic inclusion bodies in cytological specimens of blood smears (“BIBD”) as gold standard. Positive (PPV) and Negative (NPV) predictive values were calculated for BIBD prevalence on all the examined samples. Contingency tables include the numbers used in each test and their results.

| PCR test | | BIBD | | | Sensitivity % (95%CI) | Specificity %  (95%CI) | Prevalence %  (95%CI) | PPV %  (95%CI) | NPV %  (95%CI) |
| --- | --- | --- | --- | --- | --- | --- | --- | --- | --- |
|  |  | +ve | -ve | Total |  |  |  |  |  |
| UGV/S6  qRT-PCR [9] | +ve | 62 | 41 | 103 | 100.00  (100.00 -100.00) | 78.53  (73.47 - 83.59) | 24.51  (19.21 - 29.81) | 60.19  (54.16 - 66.2) | 100.00  (100.00 - 100.00) |
|  | -ve | 0 | 150 | 150 |  |  |  |  |  |
|  | Total | 62 | 191 | 253 |  |  |  |  |  |
| Multiplex  RT-PCR | +ve | 87 | 39 | 126 | 100.00  (100.00 - 100.00) | 85.00  (81.24 - 88.76) | 25.07  (20.51 - 29.63) | 69.05  (64.18 - 73.91) | 100.00  (100.00 - 100.00) |
|  | -ve | 0 | 221 | 221 |  |  |  |  |  |
|  | Total | 87 | 260 | 347 |  |  |  |  |  |

**c.** Sensitivity and specificity analysis of the multiplex RT-PCR using the UGV/S6 qRT-PCR as gold standard. Positive (PPV) and Negative (NPV) predictive values were calculated for the prevalence of reptarenavirus infection on all the examined samples. Contingency tables include the numbers used in each test and their results.

|  | | UGV/S6 qRT-PCR [9] | | | Sensitivity % (95%CI) | Specificity %  (95%CI) | Prevalence %  (95%CI) | PPV %  (95%CI) | NPV %  (95%CI) |
| --- | --- | --- | --- | --- | --- | --- | --- | --- | --- |
|  |  | +ve | -ve | Total |  |  |  |  |  |
| Multiplex RT-PCR | +ve | 96 | 1 | 97 | 100.00  (100.00 - 100.00) | 99.33  (98.31 - 100.35) | 39.18  (33.07 - 45.30) | 98.97  (97.70 - 100.23) | 100.00  (100.00 - 100.00) |
|  | -ve | 0 | 148 | 148 |  |  |  |  |  |
|  | Total | 96 | 149 | 245 |  |  |  |  |  |
